# The Dark Side of Isocyanides: Visible-Light Photocatalytic Activity in the Oxidative Functionalization of C(sp^3^)–H Bonds

**DOI:** 10.1021/acs.joc.1c02378

**Published:** 2021-12-01

**Authors:** Camilla Russo, Jussara Amato, Gian Cesare Tron, Mariateresa Giustiniano

**Affiliations:** † Department of Pharmacy, University of Naples Federico II, Via D. Montesano 49, 80131 Napoli, Italy; ‡ Department of Drug Science, University of Piemonte Orientale, Largo Donegani 2, 28100 Novara, Italy

## Abstract

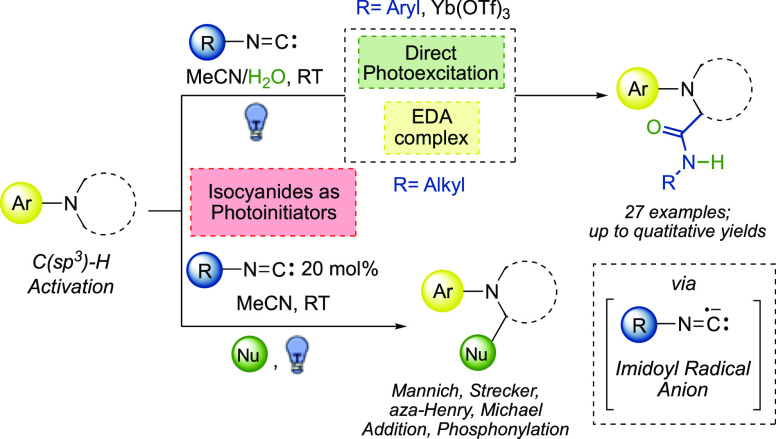

The possibility to harness aromatic isocyanides as visible-light photocatalysts in the α-amino C(sp^3^)–H functionalization is herein presented. Actually, the three-component cross-dehydrogenative coupling of aromatic tertiary amines with isocyanides and water leads to amide products under very mild conditions in high yields and with a good substrate scope. While the reaction with aromatic isocyanides proceeds upon direct photoexcitation, aliphatic isocyanides are able to form a photoactive electron–donor–acceptor complex with aromatic amines. Moreover, the use of a catalytic loading of an aromatic isocyanide promotes the oxidative coupling of *N*-phenyl-1,2,3,4-tetrahydroisoquinoline with an array of different (pro)nucleophiles in good to excellent yields, thus providing the proof-of-concept for the development of a new highly tunable class of organic visible-light photocatalysts.

## Introduction

**Figure 1:**
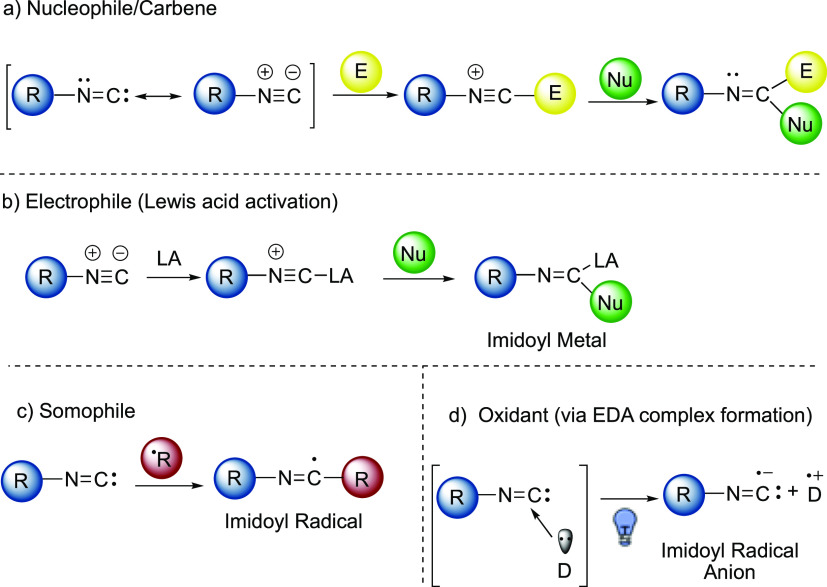
Reactivity modes of isocyanides.

Isocyanides represent a class of very complex and fascinating compounds, thanks to their chameleonic electronic properties, which have been enabling, in the last decades, the development of well-defined and stimulating research areas.^1^ Accordingly, their unique reactivity features have been widely exploited in isocyanide-based multicomponent reactions^2−4^ (nucleophile/carbene reactivity, Figure 1a) and in Lewis acid-catalyzed migratory insertions into nucleophiles^5−8^ (electrophile reactivity, Figure 1b). Furthermore, their ability to form complexes with π-electron-releasing transition metals has been shown as key to promote imidoylative cross couplings, with isocyanides undergoing 1,1-migratory insertions into either σ- or π-bonds.^9−14^ On the other hand, the presence of a lone pair on the (carbenic) isocyanide carbon atom makes them excellent geminal radical acceptors in reactions involving the formation of open-shell species (somophile reactivity, Figure 1c).^15−18^

**Figure 2:**
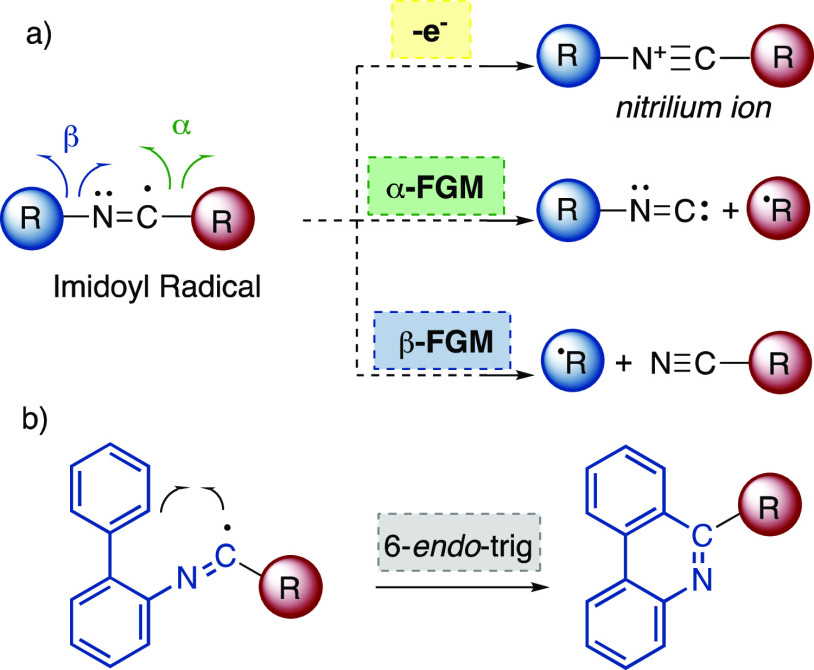
Possible reaction pathways of imidoyl radicals.

Recently, with the advent of the visible-light photocatalysis era,^19−22^ the somophile reactivity has been investigated in a plethora of transformations involving intramolecular cyclization of 2-isocyanobiphenyls and related analogues,^16,17,23^ as well as in two- or multicomponent reactions leading to amides, keto-amides, and other interesting molecular scaffolds.^24−26^ More in detail, these processes involve the formation of an imidoyl radical intermediate upon the addition of a radical species to isocyanide (Figure 1c). The imidoyl radical, depending on the reaction conditions and the species involved, can meet different fates such as oxidation to nitrilium ions, α-fragmentation (α-FGM) to give back isocyanide and the radical species, β-fragmentation (β-FGM) to nitrile (Figure 2a), and intramolecular interception of a radical acceptor (e.g., phenyl ring in the *ortho*-position of 2-isocyanobiphenyl, Figure 2b).

While most of the current literature is based on these reactivity profiles, mainly triggered by either metal-based or organic visible-light photocatalysts as well as by thermal initiators (e.g., di*tert*-butyl peroxide, DTBP; *tert*-butyl hydroperoxide, TBHP; and so forth), processes involving the formation of imidoyl radical anions have been marginally reported (Figure 1d). The latter are indeed considered quite unstable,^27^ albeit a recent involvement of their generation has been accounted upon photoinduced single-electron transfer (SET) from arylsulfinate anions following the formation of an EDA complex (EDA: electron donor–acceptor).^28^

**Scheme 1:**
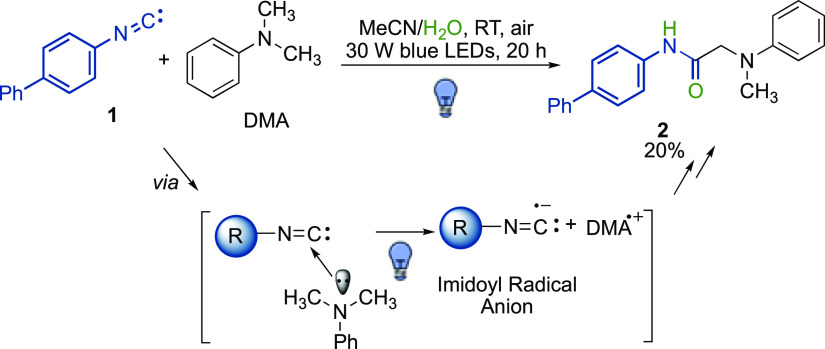
Test Reaction between Isocyanide **1** and DMA

Following our interest in isocyanide-involving photochemical reactions,^29,30^ we wondered whether the exploitation of such isocyanide radical anions could lead to developing new useful chemical transformations proceeding via EDA complex formation.^31−35^ Accordingly, a screening of possible electron donors endowed with different redox potentials (i.e., carboxylic and boronic acids and dimethylaniline, DMA) was performed. Interestingly, the test reaction of isocyanide **1** with DMA in MeCN at room temperature and under irradiation with 30 W blue light-emitting diodes (LEDs), open in air and in the presence of 10 equivalents of water, led to the formation of amide **2** in 20% yield (Scheme 1).^36^

## Results and Discussion

### Direct Photoexcitation of Aromatic Isocyanides

**Scheme 2:**
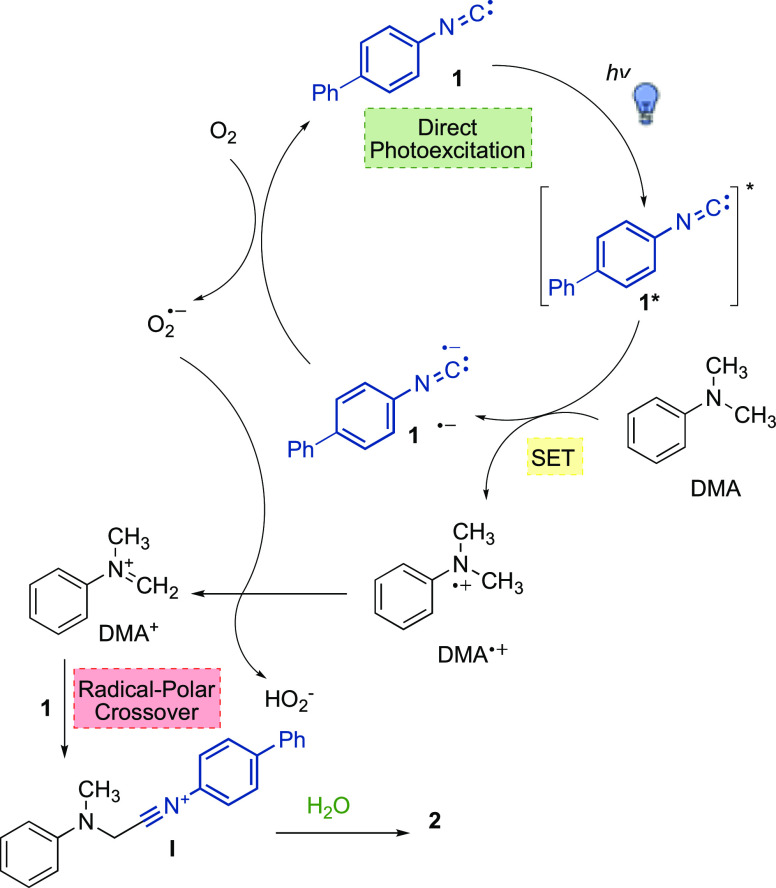
Mechanistic Hypothesis for the Formation of **2**

Intrigued by the mechanism underlying its formation and with the aim to gain information useful to optimize reaction conditions, the UV–vis absorption spectra of **1**, DMA, and a mixture of both were recorded to check the presence of an EDA complex. While no bathochromic shift was observed for the mixture of **1** and DMA, we noticed that the absorption spectrum of **1** was characterized by two bands with λ_max_ at 265 and 360 nm (Figure S1, Supporting Information). This observation prompted us to propose a mechanistic hypothesis entailing a catalytic role of isocyanide **1** upon its direct photoexcitation with visible light (Scheme 2).

More in detail, **1**, upon light absorption, reached an electronically excited state, thus acting as a photocatalyst: a SET from DMA to 1* led to the formation of the imidoyl radical anion **1**^•–^ and the radical cation of dimethylaniline DMA^•+^. Molecular oxygen was then able to regenerate isocyanide **1**, while forming a superoxide radical anion O_2_^•–^, which abstracted a hydrogen atom from DMA^•+^, thus leading to the formation of the iminium ion DMA^+^. The latter was then intercepted by the ground-state isocyanide **1**, to afford a nitrilium ion **I**, and eventually, after the addition of water, the amide **2**. Overall, formation of amide **2** proceeded via a radical/polar crossover pathway.

**Figure 3:**
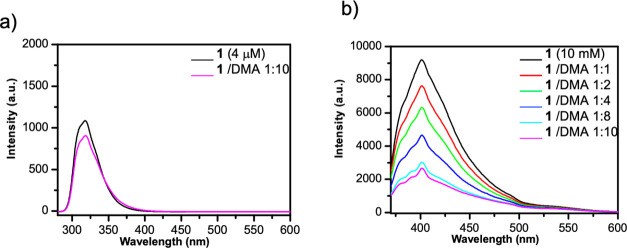
(a) Fluorescence spectra of **1** in the absence and presence of DMA (10 equiv) at 25 °C and an excitation wavelength of 265 nm and (b) Stern–Volmer fluorescence quenching of 1* with increasing amounts of DMA at 25 °C and an excitation wavelength of 360 nm.

Experimental data further supporting this mechanistic hypothesis were provided by Stern–Volmer quenching of **1** with increasing amounts of DMA (Figure 3) and by the formation of product **2–**^**18**^**O** in the presence of H_2_^18^O, as detected by means of high-resolution mass spectrometry (HRMS) analysis of the crude reaction mixture (Figure S2, Supporting Information). Taken together, such results provided a rational basis to identify optimum reaction conditions. Changing the ratio of **1**:DMA as well as the reaction times or the light source did not improve the yield of amide **2** (Table S1, Supporting Information). Therefore, we wondered if the poor yield of **2** could be ascribed to a very short half-life of imidoyl radical anion **1**^•–^, which would lead to a back-electron transfer (BET) event to isocyanide **1** and DMA, thus preventing a quantitative oxidation of DMA to DMA^+^.

In light of these observations, we reasoned that the use of a σ-electron acceptor, such as a Lewis acid, could promote the stabilization of such imidoyl radical anion, thus preventing unproductive BETs. Actually, the reaction performed in the presence of 30 mol % of Yb(OTf)_3_ afforded amide **2** in 95% isolated yield.^37^ While the irradiation with black light did not change the yield, no product was obtained when the light was carefully excluded, proving the photocatalytic nature of the transformation. Other Lewis acids such as silver, lanthanum, and copper triflates as well as transition metals and different ytterbium sources showed to have either no or poorer catalytic activity (Table S1, Supporting Information). A further survey of the minimum amount of Yb(OTf)_3_ required to get optimum yields revealed that a 10 mol % equivalent was effective with a reaction time of 20 h. A tentative scaling up to 0.25 and 0.8 mmol afforded 41 and 23% yields, respectively, with a recovery of about 50% of the starting isocyanide. Interestingly, the use of 30 W blue LEDs was crucial to the transformation, as when the reaction was performed under irradiation with 1 W blue LEDs, only traces of **2** could be detected.

**Figure 4:**
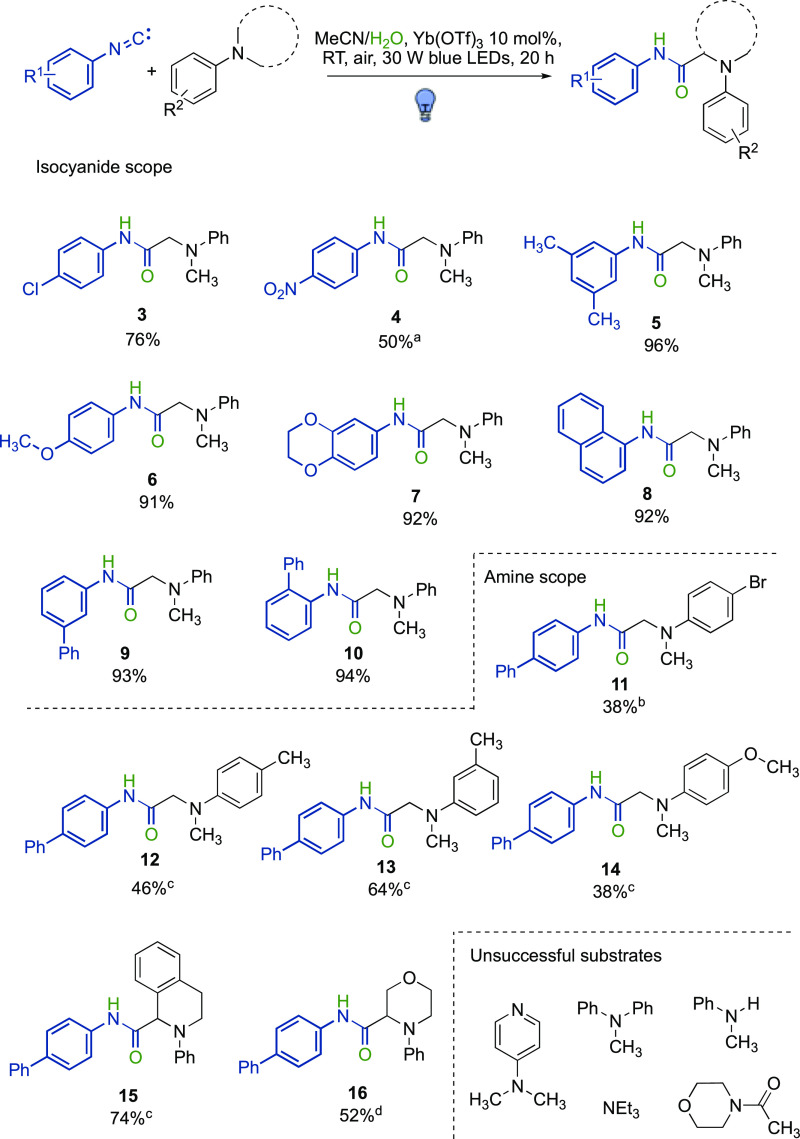
Substrate scope for aromatic isocyanides and amines [^a^without Yb(OTf)_3_; ^b^5 d; ^c^48 h; ^d^72 h].

With the optimized conditions in hand, the substrate scope of both aromatic isocyanides and amines was investigated. As shown in Figure 4, both electron-poor (**3**, **4**) and electron-rich isocyanides (**5**–**7**) gave excellent yields, with the *para*- and *meta*-substitution patterns not affecting the formation of the desired products. Interestingly, even *ortho*-isocyanobiphenyl, usually reacting intramolecularly when involved in the formation of imidoyl radical intermediates, gave the linear amide **10** in 94% yield, thus supporting the radical/polar crossover mechanistic hypothesis. As for the amine partners, both electron-poor (**11**) and electron-rich (**12**–**14**) *N*,*N*-dimethylanilines were competent starting materials, as well as cyclic tertiary aromatic amines (**15** and **16**). Heteroaromatic dimethylanilines, such as *N*,*N*-dimethyl aminopyridine, as well as *N*-methyldiphenylamine, secondary anilines, tertiary aliphatic amines, and amides, were all unsuccessful substrates. These results were in accordance with related existing reactions.^38^

### Reaction of Aliphatic Isocyanides

**Scheme 3:**
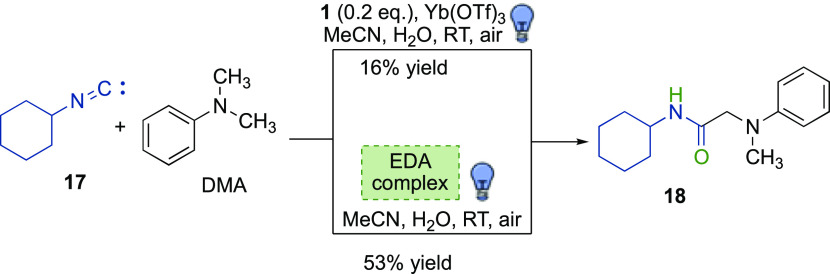
Reaction of Aliphatic Isocyanides

With the aim of further expanding the isocyanide scope to aliphatic ones and to prove the ability of aromatic isocyanides to act as photocatalysts, a test reaction involving cyclohexylisocyanide **17**, DMA, a catalytic 20 mol % loading of isocyanide **1** and 10 mol % of Yb(OTf)_3_ was performed (Scheme 3). After 20 h, the desired amide **18** was obtained in 16% yield. Optimization of reaction conditions (Table S2, Supporting Information) surprisingly led to the formation of product **18** in 53% yield in the absence of both **1** and Yb(OTf)_3_ (Scheme 3).

**Figure 5:**
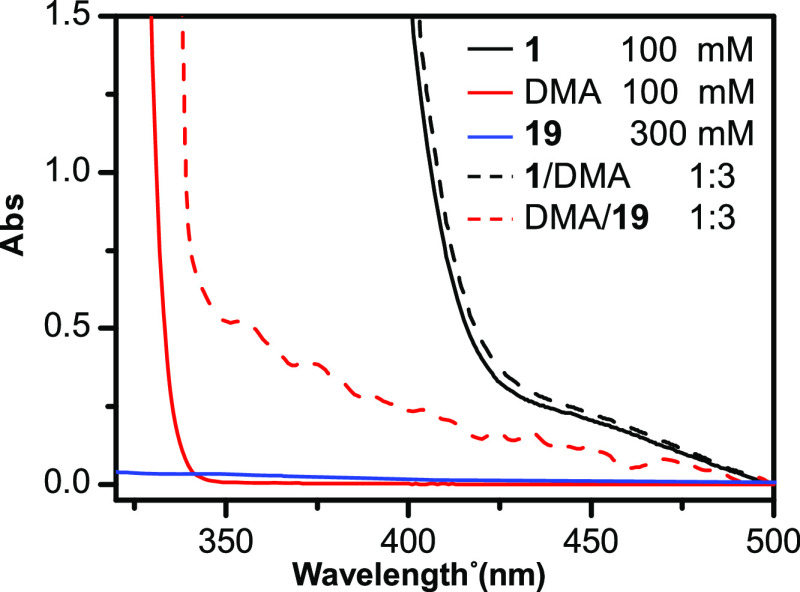
UV–Vis absorption spectra at 25 °C of **19**, DMA alone, and in combination with **19**. For comparison, spectra of isocyanide **1** alone and upon the addition of DMA were also reported.

**Scheme 4:**
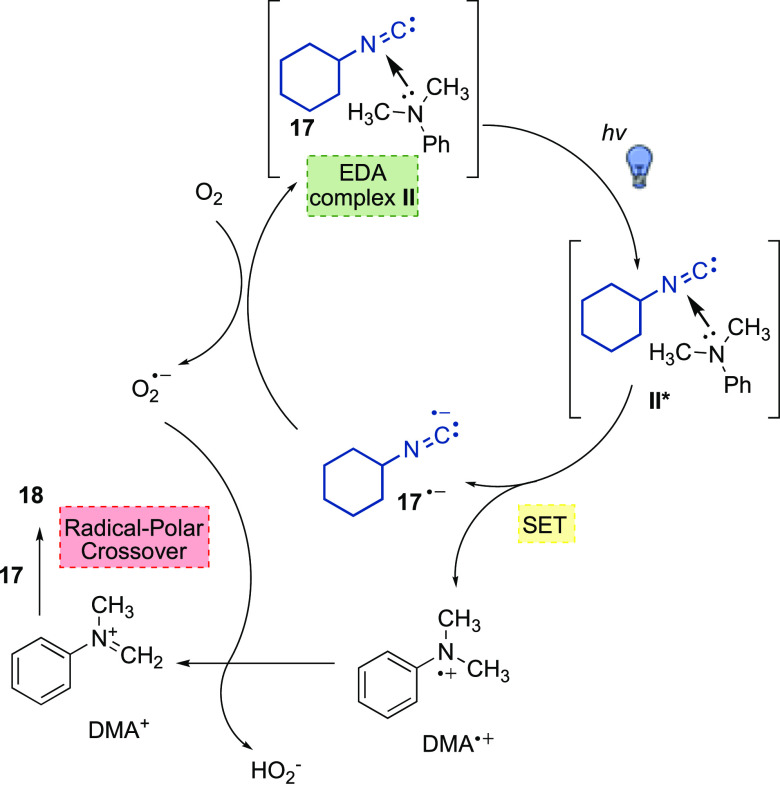
Mechanistic Hypothesis for the Reaction Involving Aliphatic Isocyanides

In order to shed light on the nature of this unexpected outcome, UV–Vis absorption spectra of DMA alone and in combination with an aliphatic nonvolatile isocyanide such as 1-adamantyl isocyanide **19** were recorded. As apparent from Figure 5, such experiments indicated a charge-transfer band due to the formation of an EDA complex between DMA and **19**, thus leading to propose the mechanistic hypothesis shown in Scheme 4. More in detail, visible-light excitation of the EDA complex **II** triggered a SET from DMA (the donor) to the isocyanide **17** (the acceptor), thus forming the radical cation DMA^•+^ and the isocyanide radical anion **17**^•–^.

The latter, similar to the pathway proposed for aromatic isocyanide **1** (Scheme 2), was oxidized back to **17** by molecular oxygen furnishing a superoxide radical anion O_2_^•–^, which was responsible for hydrogen atom abstraction from DMA^•+^, eventually affording the iminium ion DMA^+^. As stated above, overall, the formation of product **18** proceeded via a radical-polar crossover mechanism.

**Figure 6:**
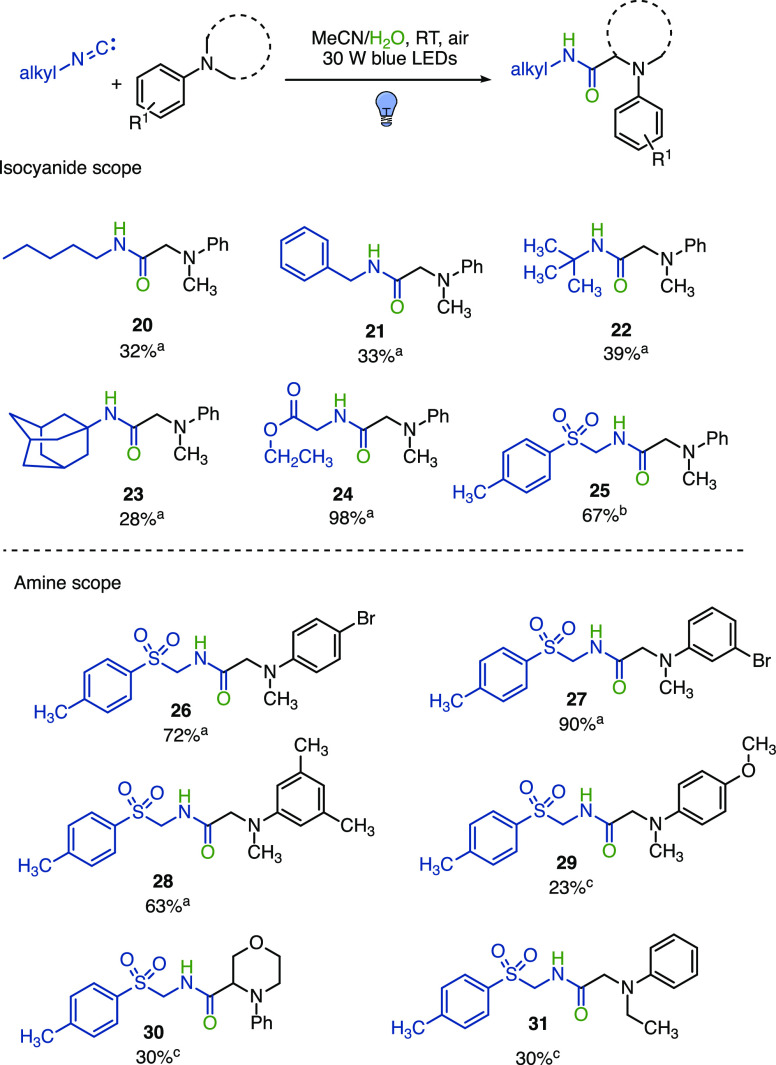
Substrate scope of aliphatic isocyanides (^a^48 h; ^b^20 h; and ^c^72 h).

As for the generality of this transformation, as shown in Figure 6, it proved to be efficient with electron-rich aliphatic primary, secondary, and bulky tertiary isocyanides (**20**–**23**), while excellent yields were obtained with isocyanides bearing electron-withdrawing groups (**24** and **25**). When *p*-toluenesulfonylmethyl isocyanide (TosMIC) was reacted with different *N*,*N*-dimethylanilines, excellent yields were observed in the presence of electron-withdrawing substituents regardless of the *para*- or *meta*-substitution pattern (**26** and **27**, Figure 6), while electron-donor substituents (**28** and **29**, Figure 6) as well as cyclic tertiary aromatic amines (**30**, Figure 6) led to yields from good to moderate. When nonsymmetrical *N*-ethyl-*N*-methylaniline was reacted as the starting amine, the selective formation of regioisomer **31** was obtained in 30% yield (Figure 6).

### Catalytic Role of Aromatic Isocyanides: Proof-of-Concept

If the unearthing of EDA complex formation between aliphatic isocyanides and tertiary aliphatic amines contributed to explain the mechanism underlying the generation of amide derivatives **18**, **20**–**31** (Figure 6), on the other hand, the need for further proving the catalytic role of aromatic isocyanides, for example **1**, was still disappointed.

**Scheme 5:**
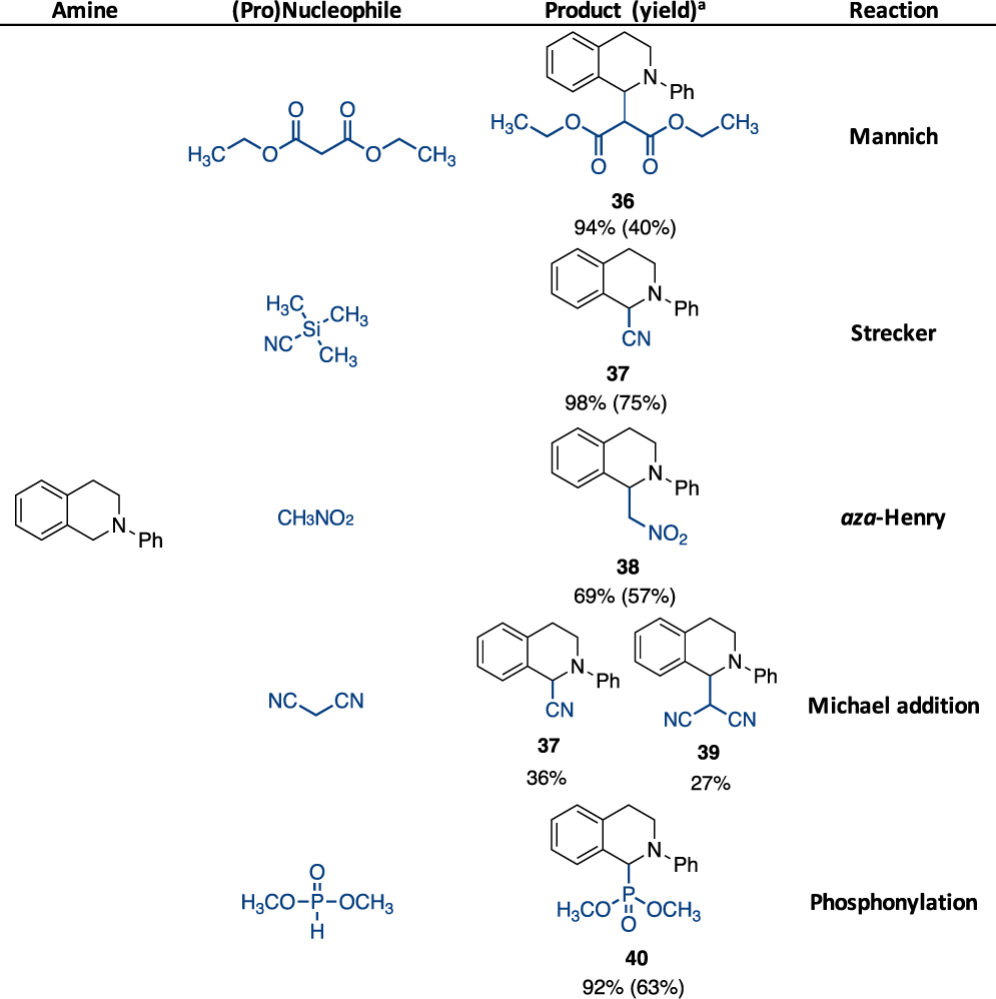
Michael-Type Addition Promoted by Isocyanide **1** as a Photocatalyst ^1^H NMR yields; in parentheses isolated yields.

Accordingly, in order to investigate whether this unexplored catalytic reactivity of isocyanides could be harnessed at a more general level, a range of both carbon and hetero-atom (pro)nucleophiles, such as diethyl malonate (Mannich-type reaction),^39^ cyanotrimethylsilane (Strecker-type reaction),^40^ nitromethane (*aza*-Henry reaction),^41^ malononitrile,^39^ and dimethyl phosphite,^42^ were made to react with *N*-phenyl-1,2,3,4-tetrahydroisoquinoline in the presence of a 20 mol % loading of isocyanide **1** (Scheme 5).

Here, the resonance-stabilized carbanions (Michael donors) were formed upon deprotonation by the *in situ* formed hydroperoxide anion HO_2_^–^, without requiring the addition of any further base. As shown in Scheme 5, all the (pro)nucleophiles gave the cross dehydrogenative coupling adducts in good to excellent yields; note that the reaction between *N*-phenyl-1,2,3,4-tetrahydroisoquinoline and diethyl malonate performed in the absence of **1** afforded a 4% yield of product **32**. Cyanotrimethylsilane and nitromethane led to the Strecker and *aza*-Henry products **33** and **34** in 98 and 69% yields, respectively. Malononitrile showed to be effective as a Michael donor under standard reaction conditions leading to a mixture of α-aminonitrile **33** and adduct **35**. These results were consistent with previous reports in the literature.^38^ Similarly, dimethyl phosphite afforded phosphonylated product **36** in 92% yield.

## Conclusions

In conclusion, the possibility to trigger completely new reactivities by enlightening a dark side of isocyanide was herein disclosed. Indeed, aromatic isocyanides proved able to act as photocatalysts triggering the oxidation of α-amino C(sp^3^)–H bonds. When stoichiometric amounts of aromatic isocyanides were used, the self-catalyzed reaction led to the formation of amide derivatives upon the radical/polar crossover reaction in good to excellent yields and with a wide substrate scope. Aliphatic isocyanides proved to form photoactive EDA complexes with aromatic amines and when combined with water, they were able to provide amide adducts under very mild and, to our knowledge, unprecedented, metal-free oxidative conditions. Importantly, it was also shown that a catalytic loading of an aromatic isocyanide can promote the cross dehydrogenative coupling of a tertiary aromatic amine, such as *N*-phenyl-1,2,3,4-tetrahydroisoquinoline, with a range of (pro)nucleophiles affording Mannich, Strecker, *aza*-Henry, Michael-addition, and phosphonylated adducts in good to excellent yields. As a whole, the experimental data provided evidence of the potential exploitation of aromatic isocyanides as a new class of highly tunable organic photocatalysts. Worth of note, isocyanides are nowadays considered a class of widely accessible compounds, with thousands of derivatives endowed with no or limited toxicity reported in the literature and readily synthesizable from the corresponding amines.^43,44^ Actually, the addition of electron-withdrawing or electron-donor groups on their aromatic moiety, as well as the ring expansion to polycyclic scaffolds, could lead to easily accessible and fine modulation of their redox and optical properties, which could expand the substrate scope to a broader range of C(sp^3^)–H bonds. Such investigations are currently in progress in our laboratories.

## Experimental Section

### General Methods

Commercially available reagents and solvents were used without further purification. Photochemical reactions were carried out using a PhotoRedOx Box (EvoluChem) with a 30 W blue LED (EvoluChem, model: HCK1012-01-008, wavelength 450 nm, LED: CREE XPE; for the emission spectrum of the light source see: https://www.hepatochem.com/photoreactors-leds-accessories/led-evoluchem/). A holder suitable for 4 mL scintillation vials (45 × 14.7 mm) has been fitted within the box: this allows a fixed sample placement distance from the light source. All NMR spectra were obtained using a Bruker AVANCE NEO 400 or 700 MHz instrument. Experiments for structure elucidation were performed in CDCl_3_ at 25 °C with a RT-DR-BF/1H-5 mm-OZ SmartProbe. High-resolution electrospray ionization mass spectrometry (ESI-MS) spectra were recorded on a Thermo LTQ Orbitrap XL mass spectrometer. The spectra were recorded by infusion into the ESI source using MeOH as the solvent. Chemical shifts (δ) are reported in parts per million (ppm) relative to the residual solvent peak. Column chromatography was performed on silica gel (70–230 mesh ASTM) using the reported eluents. Thin-layer chromatography (TLC) was carried out on 5 × 20 cm plates with a layer thickness of 0.25 mm (Silica gel 60 F_254_) to monitor the reaction by using UV as the revelation method.

### General Procedure for the Preparation of Compounds **2–16**

To a 4 mL colorless screw-cap glass vial equipped with a magnetic stir bar were added isocyanide (0.08 mmol) and Yb(OTf)_3_ (0.008 mmol, 0.1 equiv). Then, 800 μL of dry MeCN (0.1 M), 14.4 μL of microfiltered water (0.8 mmol, 10 equiv), and the aniline derivative (0.16 mmol, 2 equiv) were added into the reaction vial via a syringe. The resulting mixture was stirred open flask in a Photoredox box (EvoluChem), under 30 W blue LED irradiation, at room temperature, until the completion of the reaction, as monitored by TLC (specific reaction times are available for each compound). Then, the solvent was removed under vacuum and the crude mixture was purified by silica gel chromatography.

#### *N*-([1,1′-Biphenyl]-4-yl)-2-(methyl(phenyl)amino)acetamide (**2**)

The crude material (reaction time: 20 h) was purified by column chromatography (*n*-hexane/ethyl acetate 97:3) to give the product as a light pink solid [23.0 mg, 91% yield; 62.6 mg, 23% when the reaction was performed on a 0.8 mmol scale, with a recovery of 51% (76 mg) of the starting isocyanide (reaction time: 5dd); 41% NMR yield when the reaction was performed on a 0.25 mmol scale, with 50% of unreacted 4-isocyanobiphenyl (reaction time: 5dd)]. ^1^H NMR (700 MHz, CDCl_3_): δ 8.50 (br s, −N*H*), 7.61 (d, *J* = 8.5 Hz, 2H), 7.57–7.56 (m, 4H), 7.43 (t, *J* = 7.6 Hz, 2H), 7.34–7.31 (m, 3H), 6.92 (t, *J* = 7.4 Hz, 1H), 6.86 (d, *J* = 7.9 Hz, 2H), 3.99 (s, 2H), 3.10 (s, 3H); ^13^C{^1^H} NMR (101 MHz, CDCl_3_): δ 168.8, 149.5, 140.5, 137.5, 136.5, 129.6, 128.8, 127.6, 127.2, 126.9, 120.2, 119.5, 113.8, 60.1, 40.1; HRMS (ESI) *m*/*z*: calcd [M + H]^+^ for C_21_H_21_N_2_O^+^, 317.1648; found, [M + H]^+^ 317.1650.

#### *N*-(4-Chlorophenyl)-2-(methyl(phenyl)amino)acetamide (**3**)^36^

The crude material (reaction time: 20 h) was purified by column chromatography (*n*-hexane/ethyl acetate 97:3) to give the product as a pink solid (16.7 mg, 76% yield). ^1^H NMR (400 MHz, CDCl_3_): δ 8.43 (br s, −NH), 7.49–7.47 (m, 2H), 7.33–7.27 (m, 4H), 6.91 (t, *J* = 7.3 Hz, 1H), 6.83 (d, *J* = 8.0 Hz, 2H), 3.96 (s, 2H), 3.08 (s, 3H); ^13^C{^1^H} NMR (101 MHz, CDCl_3_): δ 168.8, 149.4, 135.8, 129.6, 129.6, 129.0, 121.2, 119.6, 113.8, 60.1, 40.2; HRMS (ESI) *m*/*z*: calcd [M + H]^+^ for C_15_H_16_ClN_2_O^+^, 275.0946; found, [M + H]^+^ 275.0943.

#### 2-(Methyl(phenyl)amino)-*N*-(4-nitrophenyl)acetamide (**4**)^44^

The crude material [reaction performed without Yb(OTf)_3_; reaction time: 5 days] was purified by column chromatography (*n*-hexane/ethyl acetate 94:6) to give the product as a yellow amorphous solid (11.5 mg, 50% yield). ^1^H NMR (700 MHz, CDCl_3_): δ 8.78 (br s, −N*H*), 8.21–8.20 (m, 2H), 7.73–7.72 (m, 2H), 7.32 (dd, *J* = 8.7, 7.4 Hz, 2H), 6.93 (t, *J* = 7.3 Hz, 1H), 6.84 (d, *J* = 8.1 Hz, 2H), 3.99 (s, 2H), 3.10 (s, 3H); ^13^C{^1^H} NMR (176 MHz, CDCl_3_): δ 169.5, 149.3, 143.8, 142.9, 129.7, 125.1, 120.0, 119.3, 114.0, 60.3, 40.4; HRMS (ESI) *m*/*z*: calcd [M + H]^+^ for C_15_H_16_N_3_O_3_^+^, 286.1186; found, [M + H]^+^ 286.1185.

#### *N*-(3,5-Dimethylphenyl)-2-(methyl(phenyl)amino)acetamide (**5**)

The crude material (reaction time: 20 h) was purified by column chromatography (*n*-hexane/ethyl acetate 97:3) to give the product as a brownish solid (20.7 mg, 96% yield). ^1^H NMR (700 MHz, CDCl_3_): δ 8.32 (br s, −N*H*), 7.32–7.29 (m, 2H), 7.16 (s, 2H), 6.90 (t, *J* = 7.3 Hz, 1H), 6.83 (d, *J* = 8.0 Hz, 2H), 6.77 (s, 1H), 3.94 (s, 2H), 3.07 (s, 3H), 2.29 (s, 6H); ^13^C{^1^H} NMR (101 MHz, CDCl_3_): δ 168.5, 149.5, 138.8, 137.1, 129.5, 126.3, 119.4, 117.6, 113.7, 60.1, 40.0, 21.3; HRMS (ESI) *m*/*z*: calcd [M + H]^+^ for C_17_H_21_N_2_O^+^, 269.1648; found, [M + H]^+^ 269.1647.

#### *N*-(4-Methoxyphenyl)-2-(methyl(phenyl)amino)acetamide (**6**)^45^

The crude material (reaction time: 20 h) was purified by column chromatography (*n*-hexane/ethyl acetate 95:5) to give the product as a brown solid (19.6 mg, 91% yield). ^1^H NMR (700 MHz, CDCl_3_): δ 8.32 (br s, −N*H*), 7.43–7.41 (m, 2H), 7.32–7.29 (m, 2H), 6.89 (t, *J* = 7.4 Hz, 1H), 6.86–6.83 (m, 4H), 3.95 (s, 2H), 3.78 (s, 3H), 3.08 (s, 3H); ^13^C{^1^H} NMR (101 MHz, CDCl_3_): δ 168.5, 156.7, 149.5, 130.4, 129.5, 121.8, 119.3, 114.2, 113.7, 59.9, 55.5, 40.1; HRMS (ESI) *m*/*z*: calcd [M + H]^+^ for C_16_H_19_N_2_O_2_^+^, 271.1441; found, [M + H]^+^ 271.1439.

#### *N*-(2,3-Dihydrobenzo[b][1,4]dioxin-6-yl)-2-(methyl(phenyl)amino)-acetamide (**7**)

The crude material (reaction time: 20 h) was purified by column chromatography (*n*-hexane/ethyl acetate 85:15) to give the product as a brownish solid (22.0 mg, 92% yield). ^1^H NMR (400 MHz, CDCl_3_): δ 8.25 (br s, −N*H*), 7.32–7.28 (m, 2H), 7.17 (d, *J* = 2.5 Hz, 1H), 6.90–6.86 (m, 2H), 6.83–6.77 (m, 3H), 4.24–4.21 (m, 4H), 3.93 (s, 2H), 3.06 (s, 3H); ^13^C{^1^H} NMR (101 MHz, CDCl_3_): δ 168.4, 149.5, 143.5, 140.7, 130.9, 129.5, 119.3, 117.2, 113.7, 113.6, 109.8, 64.4, 64.3, 59.9, 40.1; HRMS (ESI) *m*/*z*: calcd [M + H]^+^ for C_17_H_19_N_2_O_3_^+^, 299.1390; found, [M + H]^+^ 299.1390.

#### 2-(Methyl(phenyl)amino)-*N*-(naphthalen-1-yl)acetamide (**8**)^44^

The crude material (reaction time: 20 h) was purified by column chromatography (*n*-hexane/ethyl acetate 96:4) to give the product as a brown solid (21.3 mg, 92% yield). ^1^H NMR (400 MHz, CDCl_3_): δ 8.96 (br s, −N*H*), 8.08 (d, *J* = 7.5 Hz, 1H), 7.84 (d, *J* = 8.1 Hz, 1H), 7.68 (d, *J* = 8.2 Hz, 1H), 7.49 (t, *J* = 7.9 Hz, 1H), 7.45–7.42 (m, 2H), 7.38–7.34 (m, 3H), 6.97–6.92 (m, 3H), 4.12 (s, 2H), 3.21 (s, 3H); ^13^C{^1^H} NMR (101 MHz, CDCl_3_): δ 168.9, 149.3, 134.1, 131.7, 129.7, 128.8, 126.6, 126.4, 126.0, 125.8, 125.6, 120.0, 120.0, 119.6, 113.9, 59.9, 40.4; HRMS (ESI) *m*/*z*: calcd [M + H]^+^ for C_19_H_19_N_2_O^+^, 291.1492; found, [M + H]^+^ 291.1489.

#### *N*-([1,1′-Biphenyl]-3-yl)-2-(methyl(phenyl)amino)acetamide (**9**)

The crude material (reaction time: 20 h) was purified by column chromatography (*n*-hexane/ethyl acetate 98:2) to give the product as a reddish solid (23.5 mg, 93% yield). ^1^H NMR (400 MHz, CDCl_3_): δ 8.50 (br s, −N*H*), 7.73–7.72 (m, 1H), 7.59–7.55 (m, 3H), 7.44–7.40 (m, 3H), 7.38–7.29 (m, 5H), 6.91 (t, *J* = 7.3 Hz, 1H), 6.86 (d, *J* = 8.0 Hz, 2H), 3.98 (s, 2H), 3.10 (s, 3H); ^13^C{^1^H} NMR (101 MHz, CDCl_3_): δ 168.8, 149.5, 142.2, 140.6, 137.7, 129.6, 129.4, 128.7, 127.5, 127.2, 123.4, 119.5, 118.8, 118.7, 113.8, 60.2, 40.1; HRMS (ESI) *m*/*z*: calcd [M + H]^+^ for C_21_H_21_N_2_O^+^, 317.1648; found, [M + H]^+^ 317.1648.

#### *N*-([1,1′-Biphenyl]-2-yl)-2-(methyl(phenyl)amino)acetamide (**10**)

The crude material (reaction time: 20 h) was purified by column chromatography (*n*-hexane/ethyl acetate 98:2) to give the product as a reddish sticky solid (23.8 mg, 94% yield). ^1^H NMR (400 MHz, CDCl_3_): δ 8.65 (br s, −N*H*), 8.46 (d, *J* = 8.1 Hz, 1H), 7.33–7.29 (m, 1H), 7.18–7.04 (m, 7H), 7.00–6.98 (m, 2H), 6.77 (t, *J* = 7.3 Hz, 1H), 6.48 (d, *J* = 8.0 Hz, 2H), 3.74 (s, 2H), 2.58 (s, 3H); ^13^C{^1^H} NMR (101 MHz, CDCl_3_): δ 168.6, 148.7, 137.6, 134.6, 131.9, 129.8, 129.2, 129.0, 128.8, 128.5, 127.6, 124.1, 119.9, 118.8, 113.2, 59.6, 39.4; HRMS (ESI) *m*/*z*: calcd [M + H]^+^ for C_21_H_21_N_2_O^+^, 317.1648; found, [M + H]^+^ 317.1646.

#### *N*-([1,1′-Biphenyl]-4-yl)-2-((4-bromophenyl)(methyl)amino)-acetamide (**11**)

The crude material (reaction time: 5 days) was purified by column chromatography (*n*-hexane/ethyl acetate 95:5) to give the product as an off-white solid (12.1 mg, 38% yield). ^1^H NMR (700 MHz, CDCl_3_): δ 8.31 (br s, −N*H*), 7.59–7.55 (m, 6H), 7.43 (t, *J* = 7.7 Hz, 2H), 7.40–7.38 (m, 2H), 7.33 (t, *J* = 7.4 Hz, 1H), 6.71 (d, *J* = 9.0 Hz, 2H), 3.96 (s, 2H), 3.09 (s, 3H); ^13^C{^1^H} NMR (176 MHz, CDCl_3_): δ 168.2, 148.4, 140.4, 137.7, 136.3, 132.3, 128.8, 127.7, 127.2, 126.9, 120.2, 115.3, 111.8, 59.9, 40.3; HRMS (ESI) *m*/*z*: calcd [M + H]^+^ for C_21_H_20_BrN_2_O^+^, 395.0754; found, [M + H]^+^ 395.0753.

#### *N*-([1,1′-Biphenyl]-4-yl)-2-(methyl(*p*-tolyl)amino)acetamide (**12**)

The crude material (reaction time: 48 h) was purified by column chromatography (*n*-hexane/ethyl acetate 97:3) and preparative TLC (dichloromethane) to give the product as a light pink solid (12.1 mg, 46% yield). ^1^H NMR (700 MHz, CDCl_3_): δ 8.57 (br s, −N*H*), 7.61 (d, *J* = 8.6 Hz, 2H), 7.57–7.55 (m, 4H), 7.42 (t, *J* = 7.7 Hz, 2H), 7.33 (t, *J* = 7.4 Hz, 1H), 7.12 (d, *J* = 8.4 Hz, 2H), 6.77 (d, *J* = 8.5 Hz, 2H), 3.93 (s, 2H), 3.06 (s, 3H), 2.29 (s, 3H); ^13^C{^1^H} NMR (176 MHz, CDCl_3_): δ 169.0, 147.4, 140.5, 137.4, 136.6, 130.0, 129.0, 128.8, 127.6, 127.2, 126.9, 120.2, 114.1, 60.5, 40.4, 20.3; HRMS (ESI) *m*/*z*: calcd [M + H]^+^ for C_22_H_23_N_2_O^+^, 331.1805; found, [M + H]^+^ 331.1806.

#### *N*-([1,1′-Biphenyl]-4-yl)-2-(methyl(*m*-tolyl)amino)acetamide (**13**)

The crude material (reaction time: 48 h) was purified by column chromatography (*n*-hexane/ethyl acetate 98:2) to give the product as a light pink solid (16.9 mg, 64% yield). ^1^H NMR (700 MHz, CDCl_3_): δ 8.51 (br s, −N*H*), 7.62–7.61 (d, 2H), 7.57–7.56 (m, 4H), 7.43 (t, *J* = 7.7 Hz, 2H), 7.33 (t, *J* = 7.4 Hz, 1H), 7.22–7.19 (m, 1H), 6.74 (d, *J* = 7.5 Hz, 1H), 6.67–6.66 (m, 2H), 3.97 (s, 2H), 3.08 (s, 3H), 2.35 (s, 3H); ^13^C{^1^H} NMR (176 MHz, CDCl_3_): δ 168.9, 149.6, 140.5, 139.5, 137.5, 136.5, 129.4, 128.8, 127.7, 127.2, 126.9, 120.4, 120.2, 114.6, 111.0, 60.2, 40.1, 21.8; HRMS (ESI) *m*/*z*: calcd [M + H]^+^ for C_22_H_23_N_2_O^+^, 331.1805; found, [M + H]^+^ 331.1806.

#### *N*-([1,1′-Biphenyl]-4-yl)-2-((4-methoxyphenyl)(methyl)-amino)acetamide (**14**)

The crude material (reaction performed with 3 equiv of 4-methoxy-*N*,*N*-dimethylaniline; reaction time: 48 h) was purified by column chromatography (*n*-hexane/ethyl acetate 95:5) to give the product as a dark green solid (10.5 mg, 38% yield). ^1^H NMR (700 MHz, CDCl_3_): δ 8.71 (br s, −N*H*), 7.63–7.62 (m, 2H), 7.57–7.56 (m, 4H), 7.43 (t, *J* = 7.7 Hz, 2H), 7.33 (t, *J* = 7.4 Hz, 1H), 6.89–6.88 (m, 2H), 6.84–6.82 (m, 2H), 3.88 (s, 2H), 3.78 (s, 3H), 3.02 (s, 3H); ^13^C{^1^H} NMR (176 MHz, CDCl_3_): δ 169.0, 153.6, 144.0, 140.5, 137.4, 136.6, 128.8, 127.6, 127.1, 126.9, 120.1, 115.9, 114.9, 61.0, 55.7, 41.0; HRMS (ESI) *m*/*z*: calcd [M + H]^+^ for C_22_H_23_N_2_O_2_^+^, 347.1754; found, [M + H]^+^ 347.1750.

#### *N*-([1,1′-Biphenyl]-4-yl)-2-phenyl-1,2,3,4-tetrahydroisoquinoline-1-carboxamide (**15**)

The crude material (reaction time: 48 h) was purified by column chromatography (*n*-hexane/ethyl acetate 98:2) to give the product as an off-white solid (24.0 mg, 74% yield). ^1^H NMR (400 MHz, CDCl_3_): δ 8.89 (br s, −N*H*), 7.69 (d, *J* = 7.2 Hz, 1H), 7.60–7.52 (m, 6H), 7.43 (t, *J* = 7.6 Hz, 2H), 7.39–7.27 (m, 5H), 7.21 (d, *J* = 7.1 Hz, 1H), 7.06 (d, *J* = 8.2 Hz, 2H), 6.98 (t, *J* = 7.3 Hz, 1H), 5.13 (s, 1H), 4.00–3.95 (m, 1H), 3.47–3.41 (m, 1H), 3.19–3.03 (m, 2H); ^13^C{^1^H} NMR (101 MHz, CDCl_3_): δ 170.6, 149.4, 140.5, 137.3, 136.8, 134.5, 132.1, 129.6, 129.1, 128.7, 127.8, 127.7, 127.5, 127.1, 126.8 (3 C), 120.4, 120.1, 115.3, 66.4, 45.4, 28.8; HRMS (ESI) *m*/*z*: calcd [M + H]^+^ for C_28_H_25_N_2_O^+^, 405.1961; found, [M + H]^+^ 405.1957.

#### *N*-([1,1′-Biphenyl]-4-yl)-4-phenylmorpholine-3-carboxamide (**16**)

The crude material (reaction time: 72 h) was purified by column chromatography (*n*-hexane/ethyl acetate 95:5) to give the product as a light purple solid (15.0 mg, 52% yield). ^1^H NMR (700 MHz, CDCl_3_): δ 8.17 (br s, −N*H*), 7.53 (d, *J* = 7.3 Hz, 2H), 7.50 (d, *J* = 8.5 Hz, 2H), 7.44 (d, *J* = 8.6 Hz, 2H), 7.41 (t, *J* = 7.7 Hz, 2H), 7.36–7.30 (m, 3H), 7.07 (d, *J* = 8.1 Hz, 2H), 7.00 (t, *J* = 7.3 Hz, 1H), 4.20–4.19 (m, 1H), 4.16–4.15 (m, 1H), 4.10–4.09 (m, 1H), 3.96–3.89 (m, 2H), 3.53–3.50 (m, 1H), 3.32–3.29 (m, 1H); ^13^C{^1^H} NMR (176 MHz, CDCl_3_): δ 168.5, 149.6, 140.4, 137.5, 136.5, 129.9, 128.8, 127.6, 127.2, 126.9, 122.2, 120.2, 117.7, 67.7, 66.5, 62.0, 49.0; HRMS (ESI) *m*/*z*: calcd [M + H]^+^ for C_23_H_23_N_2_O_2_^+^, 359.1754; found, [M + H]^+^ 319.1754.

### General Procedure for the Preparation of Compounds **18**, **20–31**

To a 4 mL colorless screw-cap glass vial equipped with a magnetic stir bar were added isocyanide (0.08 mmol), 800 μL of dry MeCN (0.1 M), 14.4 μL of microfiltered water (0.8 mmol, 10 equiv), and the aniline derivative (0.16 mmol, 2 equiv). The resulting mixture was stirred open flask in a Photoredox box (EvoluChem), under 30 W blue LED irradiation, at room temperature, until the completion of the reaction, as monitored by TLC (specific reaction times are available for each compound). Then, the solvent was removed under vacuum and the crude mixture was purified by silica gel chromatography.

#### *N*-Cyclohexyl-2-(methyl(phenyl)amino)acetamide (**18**)^36^

The crude material (reaction time: 72 h) was purified by column chromatography (*n*-hexane/ethyl acetate 9:1) to give the product as an off-white solid (10.4 mg, 53% yield). ^1^H NMR (400 MHz, CDCl_3_): δ 7.29–7.25 (m, 2H), 6.84 (t, *J* = 7.3 Hz, 1H), 6.74 (d, *J* = 8.1 Hz, 2H), 6.44 (br d, −N*H*), 3.87–3.78 (m, 1H), 3.83 (s, 3H), 2.99 (s, 3H), 1.86–1.82 (m, 2H), 1.67–1.56 (m, 3H), 1.40–1.28 (m, 2H), 1.16–1.03 (m, 3H); ^13^C{^1^H} NMR (101 MHz, CDCl_3_): δ 169.3, 149.5, 129.3, 118.7, 113.3, 59.2, 47.8, 39.7, 33.0, 25.4, 24.7; HRMS (ESI) *m*/*z*: calcd [M + H]^+^ for C_15_H_23_N_2_O^+^, 247.1805; found, [M + H]^+^ 247.1804.

#### 2-(Methyl(phenyl)amino)-*N*-pentylacetamide (**20**)^45^

The crude material (reaction time: 48 h) was purified by column chromatography (*n*-hexane/ethyl acetate 9:1) to give the product as an off-white amorphous solid (6.0 mg, 32% yield). ^1^H NMR (700 MHz, CDCl_3_): δ 7.29–7.26 (m, 2H), 6.84 (t, *J* = 7.3 Hz, 1H), 6.74 (d, *J* = 8.3 Hz, 2H), 6.57 (br t, −N*H*), 3.85 (s, 2H), 3.27 (q, *J* = 9.4 Hz, 2H), 3.01 (s, 3H), 1.46 (m, 2H), 1.30–1.22 (m, 4H), 0.85 (t, *J* = 7.3 Hz, 3H); ^13^C{^1^H} NMR (176 MHz, CDCl_3_): δ 170.2, 149.3, 129.4, 118.6, 113.1, 59.0, 39.8, 39.2, 29.3, 29.0, 22.3, 14.0; HRMS (ESI) *m*/*z*: calcd [M + H]^+^ for C_14_H_23_N_2_O^+^, 235.1805; found, [M + H]^+^ 235.1808.

#### *N*-Benzyl-2-(methyl(phenyl)amino)acetamide (**21**)^36^

The crude material (reaction time: 48 h) was purified by column chromatography (*n*-hexane/ethyl acetate 9:1) to give the product as a brownish solid (6.7 mg, 33% yield). ^1^H NMR (400 MHz, CDCl_3_): δ 7.31–7.24 (m, 5H), 7.21–7.19 (m, 2H), 6.90 (br t, −N*H*), 6.84 (t, *J* = 7.3 Hz, 1H), 6.74 (d, *J* = 8.2 Hz, 2H), 4.49 (d, *J* = 6.0 Hz, 2H), 3.92 (s, 2H), 3.00 (s, 3H); ^13^C{^1^H} NMR (101 MHz, CDCl_3_): δ 170.4, 149.3, 138.0, 129.4, 128.7, 127.5, 127.5, 118.8, 113.3, 59.0, 43.1, 39.9; HRMS (ESI) *m*/*z*: calcd [M + H]^+^ for C_16_H_19_N_2_O^+^, 255.1492; found, [M + H]^+^ 255.1496.

#### *N*-((Methyl(phenyl)amino)methyl)pivalamide (**22**)^36^

The crude material (reaction time: 48 h) was purified by column chromatography (*n*-hexane/ethyl acetate 96:4) to give the product as an off-white amorphous solid (6.9 mg, 39% yield). ^1^H NMR (400 MHz, CDCl_3_): δ 7.29–7.25 (m, 2H), 6.85 (t, *J* = 7.3 Hz, 1H), 6.74 (d, *J* = 8.0 Hz, 2H), 6.38 (br s, −N*H*), 3.73 (s, 2H), 2.98 (s, 3H), 1.33 (s, 9H); ^13^C{^1^H} NMR (176 MHz, CDCl_3_): δ 169.6, 149.5, 129.3, 118.7, 113.4, 59.9, 50.9, 39.8, 28.7; HRMS (ESI) *m*/*z*: calcd [M + H]^+^ for C_13_H_21_N_2_O^+^, 221.1648; found, [M + H]^+^ 221.1654.

#### *N*-((3s,5s,7s)-Adamantan-1-yl)-2-(methyl(phenyl)amino)acetamide (**23**)

The crude material (reaction time: 48 h) was purified by column chromatography (*n*-hexane/ethyl acetate 9:1) to give the product as an off-white solid (6.7 mg, 28% yield). ^1^H NMR (400 MHz, CDCl_3_): δ 7.29–7.25 (m, 3H), 6.84 (t, *J* = 7.3 Hz, 1H), 6.74 (d, *J* = 8.0 Hz, 2H), 6.25 (br s, −N*H*), 3.71 (s, 2H), 2.98 (s, 3H), 2.06 (s, 3H), 1.96 (d, *J* = 2.8 Hz, 6H), 1.67–1.65 (m, 6H); ^13^C{^1^H} NMR (101 MHz, CDCl_3_): δ 169.2, 149.5, 129.3, 118.7, 113.4, 59.9, 51.6, 41.6, 39.7, 36.3, 29.4; HRMS (ESI) *m*/*z*: calcd [M + H]^+^ for C_19_H_27_N_2_O^+^, 299.2118; found, [M + H]^+^ 299.2117.

#### Ethyl *N*-Methyl-*N*-phenylglycylglycinate (**24**)^36^

The crude material (reaction time: 48 h) was purified by column chromatography (*n*-hexane/ethyl acetate 85:15) to give the product as a yellow solid (19.6 mg, 98% yield). ^1^H NMR (400 MHz, CDCl_3_): δ 7.30–7.26 (m, 2H), 7.05 (br t, −N*H*), 6.85 (t, *J* = 7.3 Hz, 1H), 6.78 (d, *J* = 8.1 Hz, 2H), 4.18 (q, *J* = 7.1 Hz, 2H), 4.06 (d, *J* = 5.7 Hz, 2H), 3.91 (s, 2H), 3.04 (s, 3H), 1.26 (t, *J* = 7.1 Hz, 3H); ^13^C{^1^H} NMR (101 MHz, CDCl_3_): δ 171.0, 169.5, 149.4, 129.4, 118.8, 113.4, 61.5, 58.8, 41.0, 39.7, 14.1; HRMS (ESI) *m*/*z*: calcd [M + H]^+^ for C_13_H_19_N_2_O_3_^+^, 251.1390; found, [M + H]^+^ 251.1391.

#### 2-(Methyl(phenyl)amino)-*N*-(tosylmethyl)acetamide (**25**)^36^

The crude material (reaction time: 20 h) was purified by column chromatography (*n*-hexane/ethyl acetate 85:15) to give the product as a white solid (17.8 mg, 67% yield). ^1^H NMR (400 MHz, CDCl_3_): δ 7.72 (d, *J* = 8.2 Hz, 2H), 7.34–7.29 (m, 4H), 6.89 (t, *J* = 7.3 Hz, 1H), 6.67 (d, *J* = 8.2 Hz, 2H), 4.68 (d, *J* = 6.9 Hz, 2H), 3.75 (s, 2H), 2.98 (s, 3H), 2.46 (s, 3H); ^13^C{^1^H} NMR (101 MHz, CDCl_3_): δ 170.3, 149.0, 145.5, 133.8, 129.9, 129.5, 128.8, 119.3, 113.5, 59.9, 58.5, 40.1, 21.8; HRMS (ESI) *m*/*z*: calcd [M + H]^+^ for C_17_H_21_N_2_O_3_S^+^, 333.1267; found, [M + H]^+^ 333.1267.

#### 2-((4-Bromophenyl)(methyl)amino)-*N*-(tosylmethyl)acetamide (**26**)^46^

The crude material (reaction time: 48 h) was purified by column chromatography (*n*-hexane/ethyl acetate 7:3) to give the product as an off-white solid (23.7 mg, 72% yield). ^1^H NMR (700 MHz, CDCl_3_): δ 7.69 (d, *J* = 8.2 Hz, 2H), 7.34–7.33 (m, 4H), 7.24 (br t, −N*H*), 6.52–6.50 (m, 2H), 4.68 (d, *J* = 6.9 Hz, 2H), 3.73 (s, 2H), 2.97 (s, 3H), 2.46 (s, 3H); ^13^C{^1^H} NMR (176 MHz, CDCl_3_): δ 169.8, 148.0, 145.7, 133.7, 132.2, 130.0, 128.8, 115.0, 111.4, 59.8, 58.3, 40.2, 21.8; HRMS (ESI) *m*/*z*: calcd [M + H]^+^ for C_17_H_20_BrN_2_O_3_S^+^, 411.0373; found, [M + H]^+^ 411.0373.

#### 2-((3-Bromophenyl)(methyl)amino)-*N*-(tosylmethyl)acetamide (**27**)^36^

The crude material (reaction time: 48 h) was purified by column chromatography (*n*-hexane/ethyl acetate 75:25) to give the product as a beige solid (29.5 mg, 90% yield). ^1^H NMR (700 MHz, CDCl_3_): δ 7.71 (d, *J* = 8.2 Hz, 2H), 7.35 (d, *J* = 8.2 Hz, 2H), 7.24 (br t, −N*H*), 6.99–6.97 (m, 1H), 6.98 (d, *J* = 7.9 Hz, 1H), 6.81–6.80 (m, 1H), 6.56 (dd, *J*_*a*_ = 8.4, *J*_*b*_ = 2.5 Hz, 1H), 4.67 (d, *J* = 6.9 Hz, 2H), 3.75 (s, 2H), 2.96 (s, 3H), 2.45 (s, 3H); ^13^C{^1^H} NMR (176 MHz, CDCl_3_): δ 169.7, 150.2, 145.7, 133.7, 130.7, 130.1, 128.8, 123.6, 121.9, 116.1, 111.8, 59.9, 57.9, 39.9, 21.8; HRMS (ESI) *m*/*z*: calcd [M + H]^+^ for C_17_H_20_BrN_2_O_3_S^+^, 411.0373; found, [M + H]^+^ 411.0377.

#### 2-((3,5-Dimethylphenyl)(methyl)amino)-*N*-(tosylmethyl)-acetamide (**28**)^36^

The crude material (reaction time: 48 h) was purified by column chromatography (*n*-hexane/ethyl acetate 8:2) to give the product as a beige sticky solid (18.1 mg, 63% yield). ^1^H NMR (700 MHz, CDCl_3_): δ 7.72 (d, *J* = 8.2 Hz, 2H), 7.35–7.32 (m, 3H), 6.55 (s, 1H), 6.33 (s, 2H), 4.67 (d, *J* = 6.9 Hz, 2H), 3.73 (s, 2H), 2.95 (s, 3H), 2.45 (s, 3H), 2.30 (s, 6H); ^13^C{^1^H} NMR (176 MHz, CDCl_3_): δ 170.6, 149.2, 145.5, 139.2, 133.9, 130.0, 128.8, 121.2, 111.4, 59.9, 58.6, 40.1, 21.8, 21.7. HRMS (ESI) *m*/*z*: calcd [M + H]^+^ for C_19_H_25_N_2_O_3_S^+^, 361.1580; found, [M + H]^+^ 361.1579.

#### 2-((4-Methoxyphenyl)(methyl)amino)-*N*-(tosylmethyl)-acetamide (**29**)^45^

The crude material (reaction time: 72 h) was purified by column chromatography (*n*-hexane/ethyl acetate 75:25) to give the product as a brownish sticky solid (6.8 mg, 23% yield). ^1^H NMR (700 MHz, CDCl_3_): δ 7.72 (d, *J* = 8.1 Hz, 2H), 7.53 (br t, −N*H*), 7.33 (d, *J* = 8.1 Hz, 2H), 6.85 (d, *J* = 9.0 Hz, 2H), 6.67 (d, *J* = 9.0 Hz, 2H), 4.70 (d, *J* = 6.9 Hz, 2H), 3.78 (s, 3H), 3.65 (s, 2H), 2.91 (s, 3H), 2.45 (s, 3H); ^13^C{^1^H} NMR (176 MHz, CDCl_3_): δ 170.5, 153.6, 145.6, 143.7, 133.8, 130.0, 128.8, 115.7, 114.8, 59.9, 59.6, 55.7, 41.1, 21.8; HRMS (ESI) *m*/*z*: calcd [M + H]^+^ for C_18_H_23_N_2_O_4_S^+^, 363.1373; found, [M + H]^+^ 363.1375.

#### 4-Phenyl-*N*-(tosylmethyl)morpholine-3-carboxamide (**30**)

The crude material (reaction time: 72 h) was purified by column chromatography (*n*-hexane/ethyl acetate 75:25) to give the product as a white solid (9.0 mg, 30% yield). ^1^H NMR (700 MHz, CDCl_3_): δ 7.59 (d, *J* = 8.1 Hz, 2H), 7.33 (t, *J* = 7.8 Hz, 2H), 7.27 (d, *J* = 8.0 Hz, 2H), 7.02–6.99 (m, 2H), 6.91 (d, *J* = 8.3 Hz, 2H), 4.73–4.69 (m, 1H), 4.47–4.44 (m, 1H), 3.96–3.94 (m, 1H), 3.90–3.88 (m, 2H), 3.82–3.78 (m, 2H), 3.43–3.40 (m, 1H), 3.24–3. 21 (m, 1H), 2.43 (s, 3H); ^13^C{^1^H} NMR (176 MHz, CDCl_3_): δ 170.1, 149.2, 145.4, 133.9, 129.9, 129.8, 128.7, 121.9, 117.1, 67.7, 66.5, 60.6, 59.9, 48.1, 21.8; HRMS (ESI) *m*/*z*: calcd [M + H]^+^ for C_19_H_23_N_2_O_4_S^+^, 375.1373; found, [M + H]^+^ 375.1375.

#### 2-(Ethyl(phenyl)amino)-*N*-(tosylmethyl)acetamide (**31**)^36^

The crude material (reaction time: 72 h) was purified by column chromatography (*n*-hexane/ethyl acetate 85:15) to give the product as a beige amorphous solid (8.3 mg, 30% yield). ^1^H NMR (700 MHz, CDCl_3_): δ 7.68–7.67 (m, 2H), 7.32–7.26 (m, 4 H), 6.87–6.85 (m, 1H), 6.65 (d, *J* = 6.3 Hz, 2H), 4.66–4.65 (m, 2H), 3.73 (s, 2H), 3.43–3.39 (m, 2H), 2.45 (s, 3H), 1.18–1.16 (m, 3H); ^13^C{^1^H} NMR (176 MHz, CDCl_3_): δ 170.4, 147.3, 145.5, 133.8, 129.9, 129.6, 128.8, 119.0, 113.7, 59.8, 55.4, 46.5, 21.8, 11.5; HRMS (ESI) *m*/*z*: calcd [M + H]^+^ for C_18_H_23_N_2_O_3_S^+^, 347.1424; found, [M + H]^+^ 347.1426.

### General Procedure for the Preparation of Compounds **32**, **33**, and **36**

To a 4 mL colorless screw-cap glass vial equipped with a magnetic stir bar were added the pro-nucleophile (0.08 mmol), *N*-phenyl-1,2,3,4-tetrahydroisoquinoline (33.4 mg, 0.16 mmol, 2 equiv.), and 800 μL of dry MeCN (0.1 M). Then, 4-isocyanobiphenyl **1** (2.9 mg, 0.016 mmol, 20% mol) was added to the resulting mixture, which was stirred open flask in a Photoredox box (EvoluChem), under 30 W blue LED irradiation, at room temperature, for 20 h. After the completion of the reaction, as monitored by TLC, the solvent was removed under vacuum and the crude mixture was purified by silica gel chromatography.

#### Diethyl 2-(2-Phenyl-1,2,3,4-tetrahydroisoquinolin-1-yl)malonate (**32**)^39^

The crude material was purified by column chromatography (*n*-hexane + triethylamine 0.1% v/v) to give the product as a yellowish sticky solid (11.7 mg, 40% isolated yield; NMR yield: 94%). ^1^H NMR (400 MHz, CDCl_3_): δ 7.30–7.08 (m, 6H), 6.98 (d, *J* = 8.1 Hz, 2H), 6.75 (t, *J* = 7.3 Hz, 1H), 5.72 (d, *J* = 9.1 Hz, 1H), 4.16–3.95 (m, 4H), 3.89 (d, *J* = 9.2 Hz, 1H), 3.71–3.62 (m, 2H), 3.11–3.03 (m, 1H), 2.91–2.85 (m, 1H), 1.17 (t, *J* = 7.1 Hz, 3H), 1.09 (t, *J* = 7.1 Hz, 3H); ^13^C{^1^H} NMR (176 MHz, CDCl_3_): δ 168.0, 167.2, 148.9, 136.0, 134.83, 129.1, 128.9, 127.5, 127.2, 126.0, 118.5, 115.1, 61.6, 59.6, 57.9, 42.3, 26.1, 13.9, 13.9; HRMS (ESI) *m*/*z*: calcd [M + H]^+^ for C_22_H_26_NO_4_^+^, 368.1856; found, [M + H]^+^ 368.1856.

#### 2-Phenyl-1,2,3,4-tetrahydroisoquinoline-1-carbonitrile (**33**)^40^

The crude material was purified by column chromatography (*n*-hexane + triethylamine 0.05% v/v) to give the product as a yellowish solid (14.0 mg, 75% isolated yield; NMR yield: 98%). ^1^H NMR (700 MHz, CDCl_3_): δ 7.39–7.36 (m, 2H), 7.34–7.27 (m, 3H), 7.25 (d, *J* = 7.5 Hz, 1H), 7.10 (d, *J* = 8.0 Hz, 2H), 7.03 (t, *J* = 7.3 Hz, 1H), 5.53 (s, 1H), 3.80–3.77 (m, 1H), 3.52–3.48 (m, 1H), 3.20–3.15 (m, 1H), 3.00–2.97 (m, 1H); ^13^C{^1^H} NMR (176 MHz, CDCl_3_): δ 148.4, 134.6, 129.6, 129.4, 128.8, 127.1, 126.9, 121.9, 117.8, 117.6, 53.3, 44.2, 28.6. HRMS (ESI) *m*/*z*: calcd [M + H]^+^ for C_16_H_15_N_2_^+^, 235.1230; found, [M + H]^+^ 235.1232.

#### Dimethyl (2-Phenyl-1,2,3,4-tetrahydroisoquinolin-1-yl)phosphonate (**36**)^42^

The crude material was purified by column chromatography (*n*-hexane/ethyl acetate 85:15) to give the product as a yellow sticky solid (16.1 mg, 63% yield). ^1^H NMR (700 MHz, CDCl_3_): δ 7.39–7.37 (m, 2H), 7.30–7.28 (m, 2H), 7.24–7.18 (m, 2H), 7.00 (d, *J* = 7.7 Hz, 2H), 6.84 (t, *J* = 7.0 Hz, 1H), 5.23 (d, *J* = 20.3 Hz, 1H), 4.06–4.02 (m, 1H), 3.69 (d, *J* = 10.5 Hz, 3H), 3.67 (d, *J* = 10.5 Hz, 3H), 3.67–3.65 (m, 1H), 3.11–3.01 (m, 2H); ^13^C{^1^H} NMR (176 MHz, CDCl_3_): δ 149.2 (d, *J*_C–P_ = 6.3 Hz), 136.4 (d, *J*_C–P_ = 6.0 Hz), 130.4, 129.3, 128.8 (d, *J*_C–P_ = 2.5 Hz), 127.9 (d, *J*_C–P_ = 4.9 Hz), 127.5 (d, *J*_C–P_ = 3.7 Hz), 126.0 (d, *J*_C–P_ = 2.5 Hz), 118.7, 114.8, 58.8 (d, *J*_C–P_ = 160.0 Hz), 53.9 (d, *J*_C–P_ = 6.9 Hz), 52.9 (d, *J*_C–P_ = 7.6 Hz), 43.6, 26.7; HRMS (ESI) *m*/*z*: calcd [M + K]^+^ for C_17_H_20_KNO_3_P^+^, 356.0812; found, [M + K]^+^ 356.0813.

#### 1-(Nitromethyl)-2-phenyl-1,2,3,4-tetrahydroisoquinoline (**34**)^41^

To a 4 mL colorless screw-cap glass vial equipped with a magnetic stir bar were added *N*-phenyl-1,2,3,4-tetrahydroisoquinoline (16.7 mg, 0.08 mmol), nitromethane (130 μL, 2.4 mmol, 30 equiv.), and 800 μL of dry MeCN (0.1 M). Then, 4-isocyanobiphenyl **1** (2.9 mg, 0.016 mmol, 20% mol) was added to the resulting mixture, which was stirred open flask in a Photoredox box (EvoluChem), under 30 W blue LED irradiation, at room temperature, for 20 h. After the completion of the reaction, as monitored by TLC, the solvent and the excess of nitromethane were removed under vacuum and the crude mixture was purified by preparative TLC (*n*-hexane/tetrahydrofuran 8:2) to give the product as a yellow amorphous solid (12,2 mg, 57% isolated yield; NMR yield: 69%). ^1^H NMR (700 MHz, CDCl_3_): δ 7.29–7.25 (m, 3H), 7.22–7.19 (m, 2H), 7.14 (d, *J* = 7.5 Hz, 1H), 6.98 (d, *J* = 8.3 Hz, 2H), 6.85 (t, *J* = 7.3 Hz, 1H), 5.55 (t, *J* = 7.2 Hz, 1H), 4.88 (dd, *J*_*a*_ = 11.9, *J*_*b*_ = 7.8 Hz, 1H), 4.57 (dd, *J*_*a*_ = 11.9, *J*_*b*_ = 6.7 Hz, 1H), 3.69–3.61 (m, 2H), 3.11–3.07 (m, 1H), 2.82–2.78 (m, 1H); ^13^C{^1^H} NMR (176 MHz, CDCl_3_): δ 148.4, 135.3, 132.9, 129.5, 129.2, 128.1, 127.0, 126.7, 119.4, 115.1, 78.8, 58.2, 42.1, 26.5; HRMS (ESI) *m*/*z*: calcd [M + H]^+^ for C_16_H_17_N_2_O_2_^+^, 269.1285; found, [M + H]^+^ 269.1285.

#### 2-(2-Phenyl-1,2,3,4-tetrahydroisoquinolin-1-yl)malononitrile (**35**)^39^

To a 4 mL colorless screw-cap glass vial equipped with a magnetic stir bar were added *N*-phenyl-1,2,3,4-tetrahydroisoquinoline (16.7 mg, 0.08 mmol), malononitrile (26.6 μL, 0.48 mmol, 6 equiv.), and 800 μL of dry MeCN (0.1 M). Then, 4-isocyanobiphenyl 1 (2.9 mg, 0.016 mmol, 20% mol) was added to the resulting mixture, which was stirred open flask in a Photoredox box (EvoluChem), under 30 W blue LED irradiation, at room temperature, for 20 h. After the completion of the reaction, as monitored by TLC, the solvent and the excess of malononitrile were removed under vacuum and the crude ^1^H NMR spectrum in the presence of 1,3,5-trimethoxybenzene as the internal standard was registered (NMR yield: 27%).

## Supporting Information Available

Absorption/fluorescence spectra, complete experimental procedures, characterization data, ^1^H- and ^13^C NMR, HRMS, and absorption/fluorescence spectra (PDF)
