# Supplementary material for: The Dark Side of Isocyanides: Visible-Light Photocatalytic Activity in the Oxidative Functionalization of C(sp3)–H Bonds
Source: J Org Chem. 2021 Dec 1;86(24):18117–27. doi: 10.1021/acs.joc.1c02378 (PMC8689654; doi:10.1021/acs.joc.1c02378)

SUPPORTING INFORMATION

The Dark Side of Isocyanides: Visible-Light Photocatalytic Activity in the Oxidative Functionalization of C(sp^3)-H Bonds

Camilla Russo,[†] Jussara Amato,[†] Gian Cesare Tron,[‡] and Mariateresa Giustiniano*,[†]

[†]Department of Pharmacy, University of Naples Federico II, via D. Montesano 49, 80131, Napoli, Italy
[‡]Department of Drug Science, University of Piemonte Orientale, Largo Donegani 2, 28100, Novara, Italy

Contents:

- UV-Vis absorption spectra of isocyanide **1**..……………………......………………...…..S3

- HRMS spectrum for control experiment...………………………………………..……..S4

- Optimization of reaction conditions…………………………………………………..S5

- UV-Vis absorption spectra of complex Yb-isocyanide **1**…………………………………..S7

- Copies of ^{1}H and ^{13}C spectra………..………………………………...……........S8

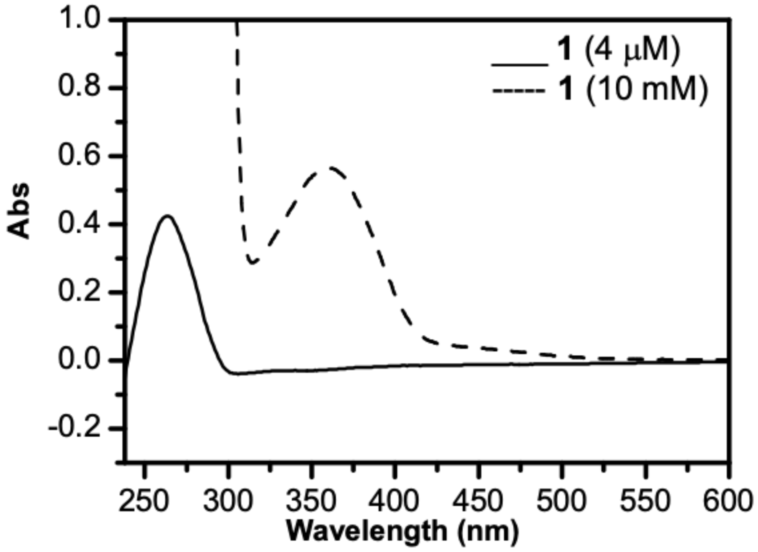

Figure S1. Uv-Vis spectra of isocyanide **1** at 25 °C (λ_{max} = 265 and 360 nm).

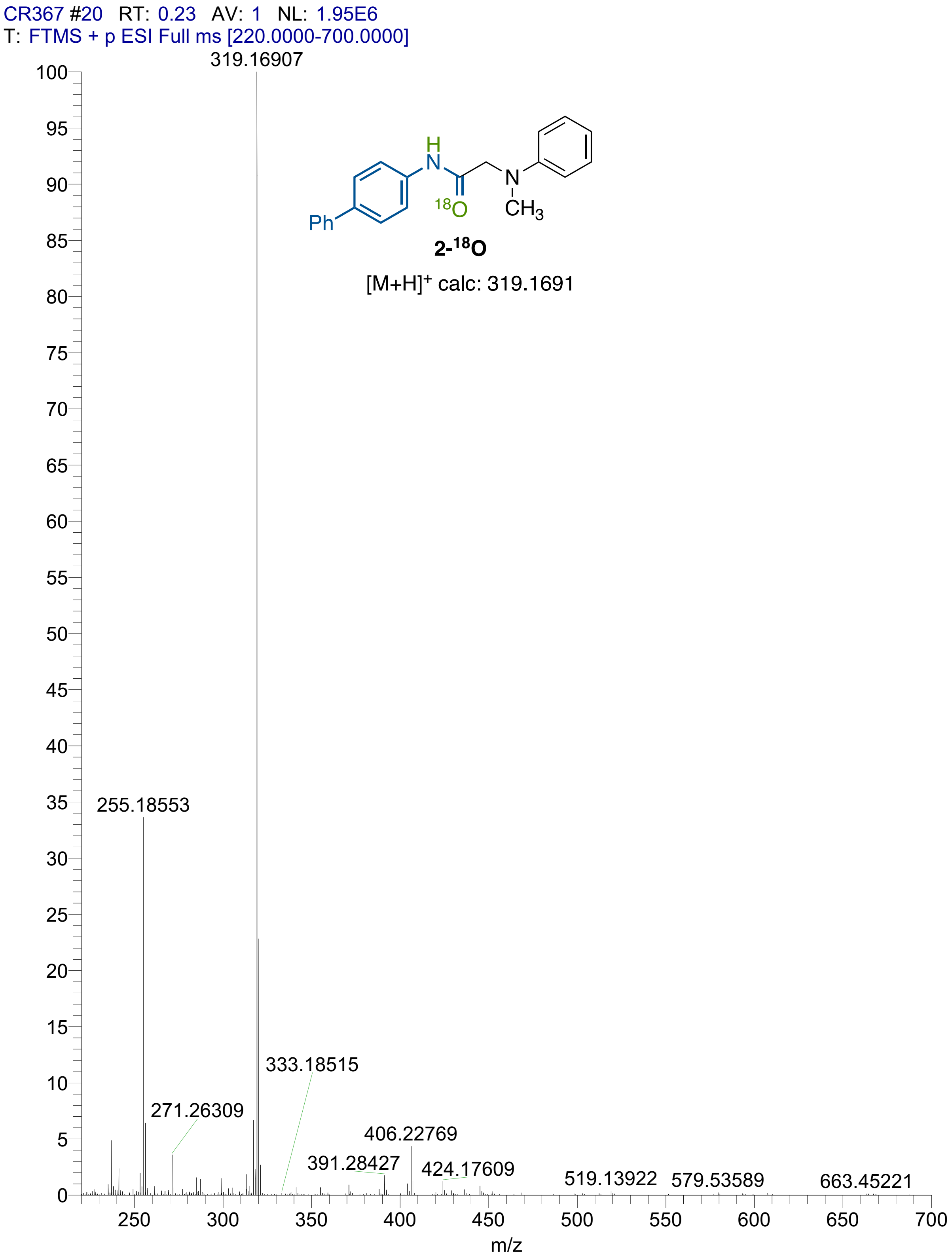

Figure S2. HRMS spectrum of the reaction performed with $H_2{}^{18}O$.

Table S1. Optimization of reaction conditions.

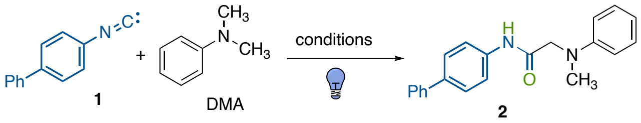

entry	Eq of DMA[a]	Light Source	LA (eq)	Reaction Time	Yield (%)[b]
**1**	2	Blue LEDs 30 W	-	20 h	20
**2**	0.7	Blue LEDs 30 W	-	20 h	14
**3**	2	Blue LEDs 30 W	-	48 h	21
**4**	2	Black light 18 W	-	20 h	14
**5**	2	Blue LEDs 30 W	$Yb(OTf)_3$ (0.3)	20 h	99 (95)[c]
**6**	2	Black light 18 W	$Yb(OTf)_3$ (0.3)	20 h	99
**7**	2	No one	$Yb(OTf)_3$ (0.3)	20 h	Traces
**8**	2	Blue LEDs 30 W	AgOTf (0.3)	20 h	14
**9**	2	Blue LEDs 30 W	$La(OTf)_3$ (0.3)	20 h	7
**10**	2	Blue LEDs 30 W	$Cu(OTf)_2$ (0.3)	20 h	14
**11**	2	Blue LEDs 30 W	$Cu(OAc)_2 \bullet H_2O$ (0.3)	20 h	14
**12**	2	Blue LEDs 30 W	$CuCl_2$ (0.3)	20 h	21
**13**	2	Blue LEDs 30 W	$Pd(OAc)_2$ (0.3)	20 h	Traces
**14**	2	Blue LEDs 30 W	$Ni(OTf)_2$ (0.3)	20 h	Traces
**15**	2	Blue LEDs 30 W	$Yb(OAc)_3 \bullet 4H_2O$ (0.3)	20 h	13
**16**	2	Blue LEDs 30 W	$YbCl_2 \bullet 6H_2O$ (0.3)	20 h	35
**17**	2	Blue LEDs 30 W	$Yb(OTf)_3$ (0.01)	20 h	52
**18**	2	Blue LEDs 30 W	$Yb(OTf)_3$ (0.05)	20 h	76
**19**	2	Blue LEDs 30 W	$Yb(OTf)_3$ (0.1)	20 h	99 (91)[c]
**20**	2	Blue LEDs 30 W	$Yb(OTf)_3$ (0.05)	48 h	94

[a]Compound **1** 0.08 mmol in MeCN 0.1 M, H_2O 10 equiv., RT**;** [b] ^{1}H-NMR yields; [c]isolated yield.

Table S2. Optimization of reaction conditions.

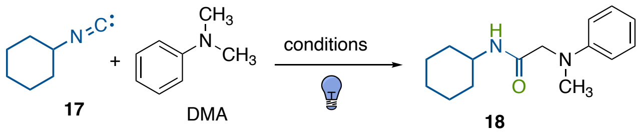

entry	Eq of DMA[a]	PC (eq)	LA (eq)	Reaction Time	Yield (%)[b]
**1**	2	Isocyanide 1 (0.2)	$Yb(OTf)_3$ (0.1)	20 h	16
**2**	2	Isocyanide 1 (0.2)	$Yb(OTf)_3$ (0.1)	48 h	32
**3**	2	Isocyanide 1 (0.2)	$Yb(OTf)_3$ (0.1)	72 h	34
**4**	0.7	Isocyanide 1 (0.2)	$Yb(OTf)_3$ (0.1)	48h	21
**5**	2	-	$Yb(OTf)_3$ (0.1)	48 h	18
**6**	2	-	-	72 h	53

[a]Compound **17** 0.08 mmol in MeCN 0.1 M, H_2O 10 equiv., RT; [b]isolated yield.

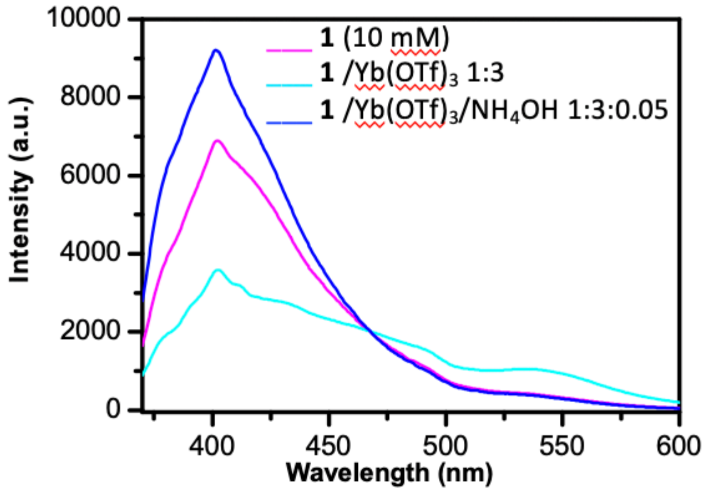

Figure S3. Superimposition of the fluorescence spectra (360 nm excitation wavelength) of isocyanide **1** in the absence and presence of $Yb(OTf)_3$ (formation of an Yb-isocyanide complex), and upon addition of ammonium hydroxide (disruption of the Yb-isocyanide complex and restoration of the fluorescence band of **1**).

^{1}H NMR
(700 MHz, $CDCl_3$)

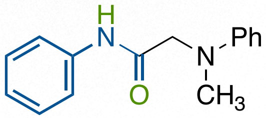

2

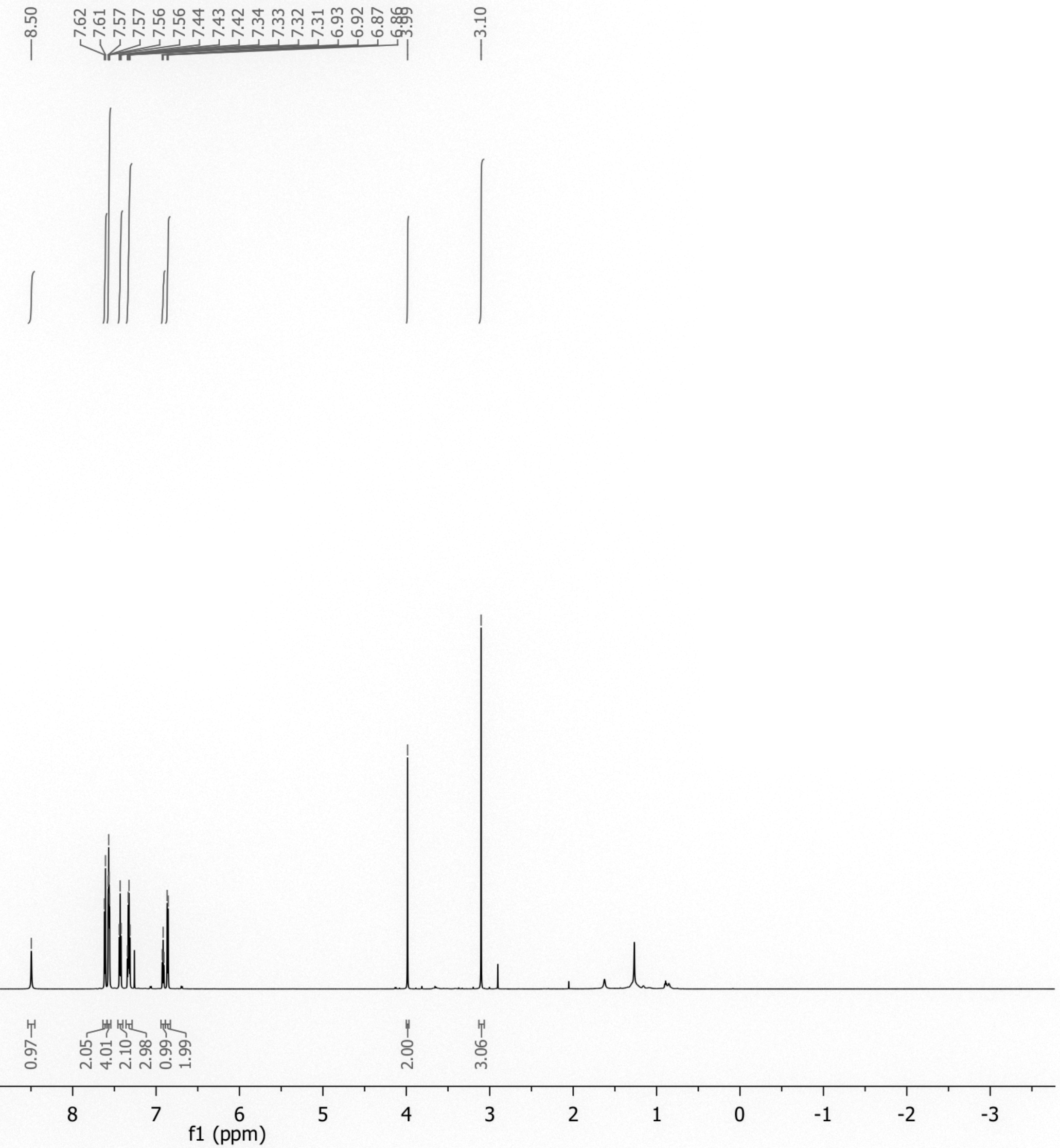

$^{13}C\{^{1}H\}$ NMR
101 MHz, $CDCl_3$

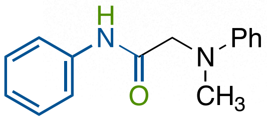

2

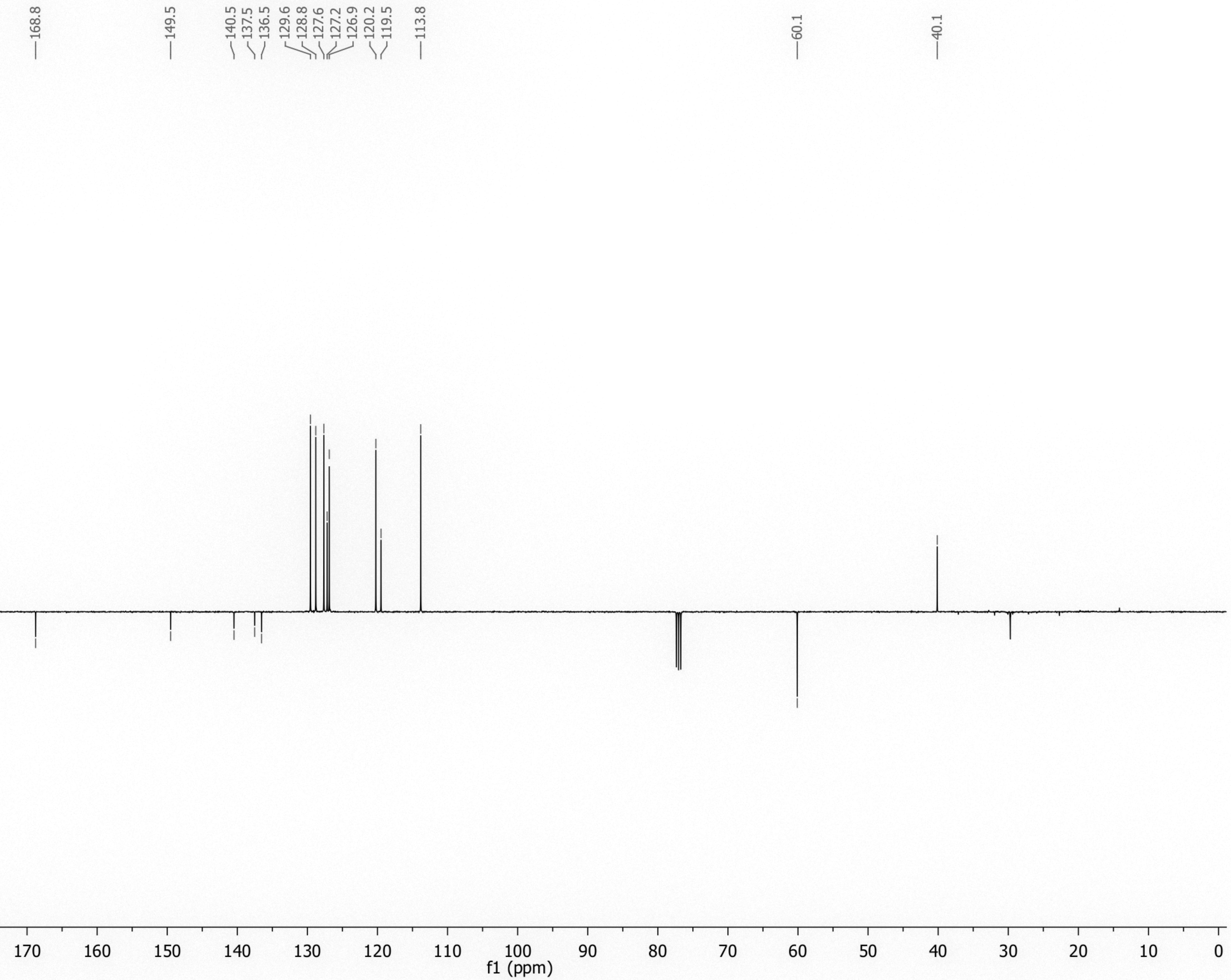

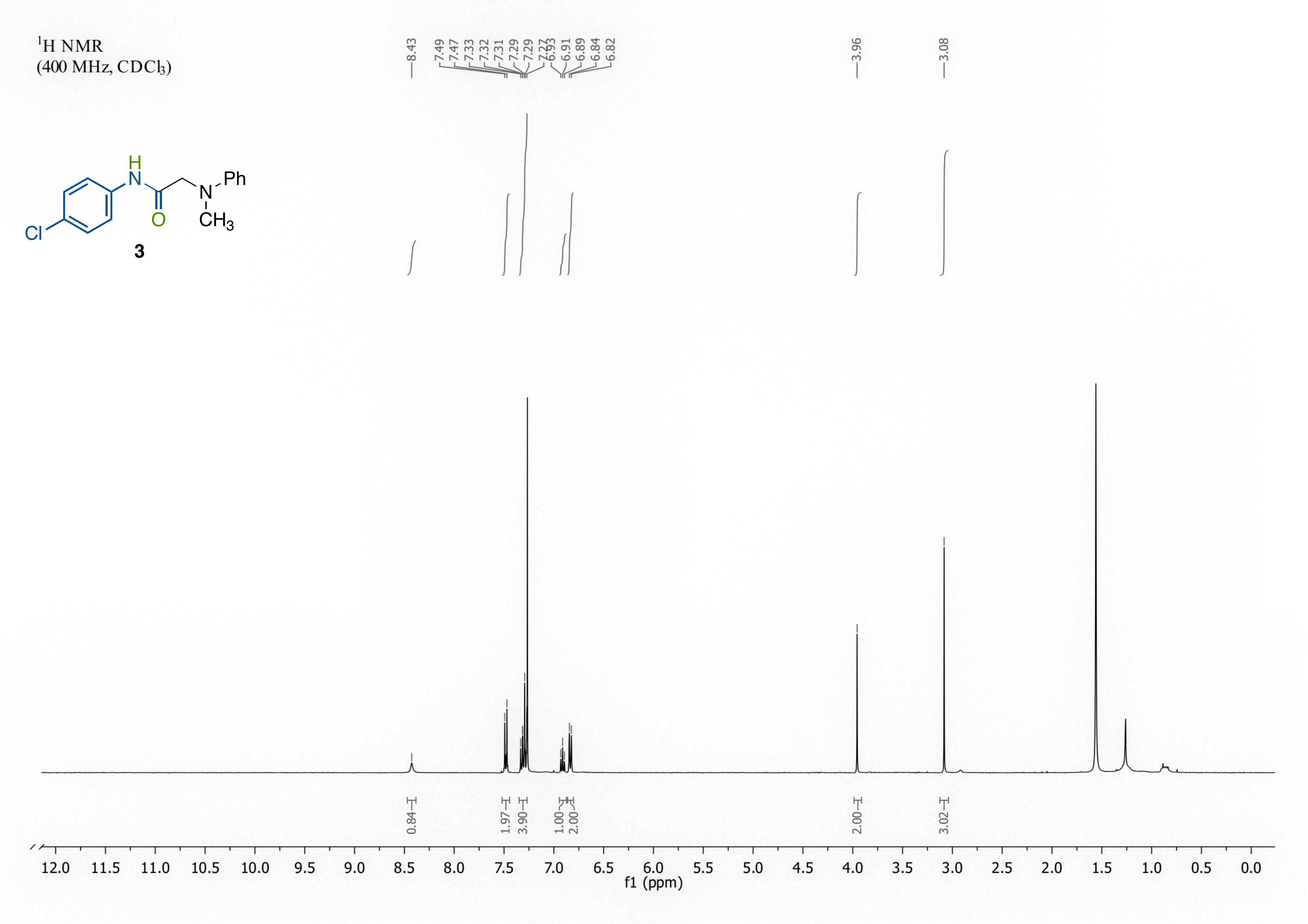

1H NMR
(400 MHz, CDCl3)
Cl
H
N
O
N
Ph
CH3
3
8.43
7.49
7.47
7.33
7.32
7.31
7.29
7.29
7.27
6.93
6.91
6.89
6.84
6.82
3.96
3.08
0.84
1.97
3.90
1.00
2.00
2.00
3.02
12.0 11.5 11.0 10.5 10.0 9.5 9.0 8.5 8.0 7.5 7.0 6.5 6.0 5.5 5.0 4.5 4.0 3.5 3.0 2.5 2.0 1.5 1.0 0.5 0.0
f1 (ppm)

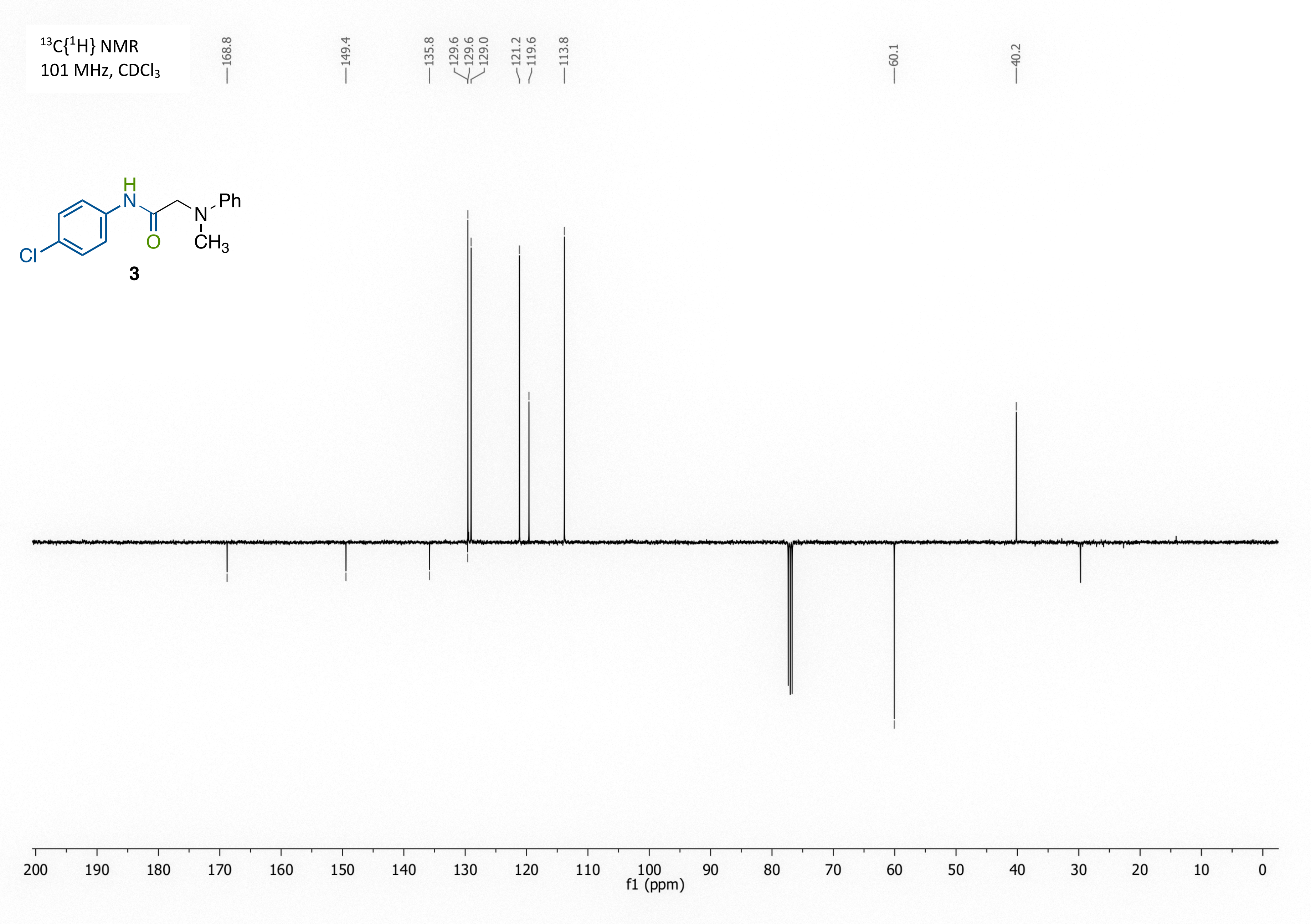

$^{13}C\{^1H\}$ NMR
101 MHz, $CDCl_3$
168.8
149.4
135.8
129.6
129.6
129.0
121.2
119.6
113.8
60.1
40.2
Cl
H
N
O
N
Ph
CH_3
3
200
190
180
170
160
150
140
130
120
110
100
90
80
70
60
50
40
30
20
10
0
f1 (ppm)

^{1}H NMR
(700 MHz, $CDCl_3$)

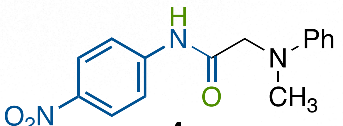

4

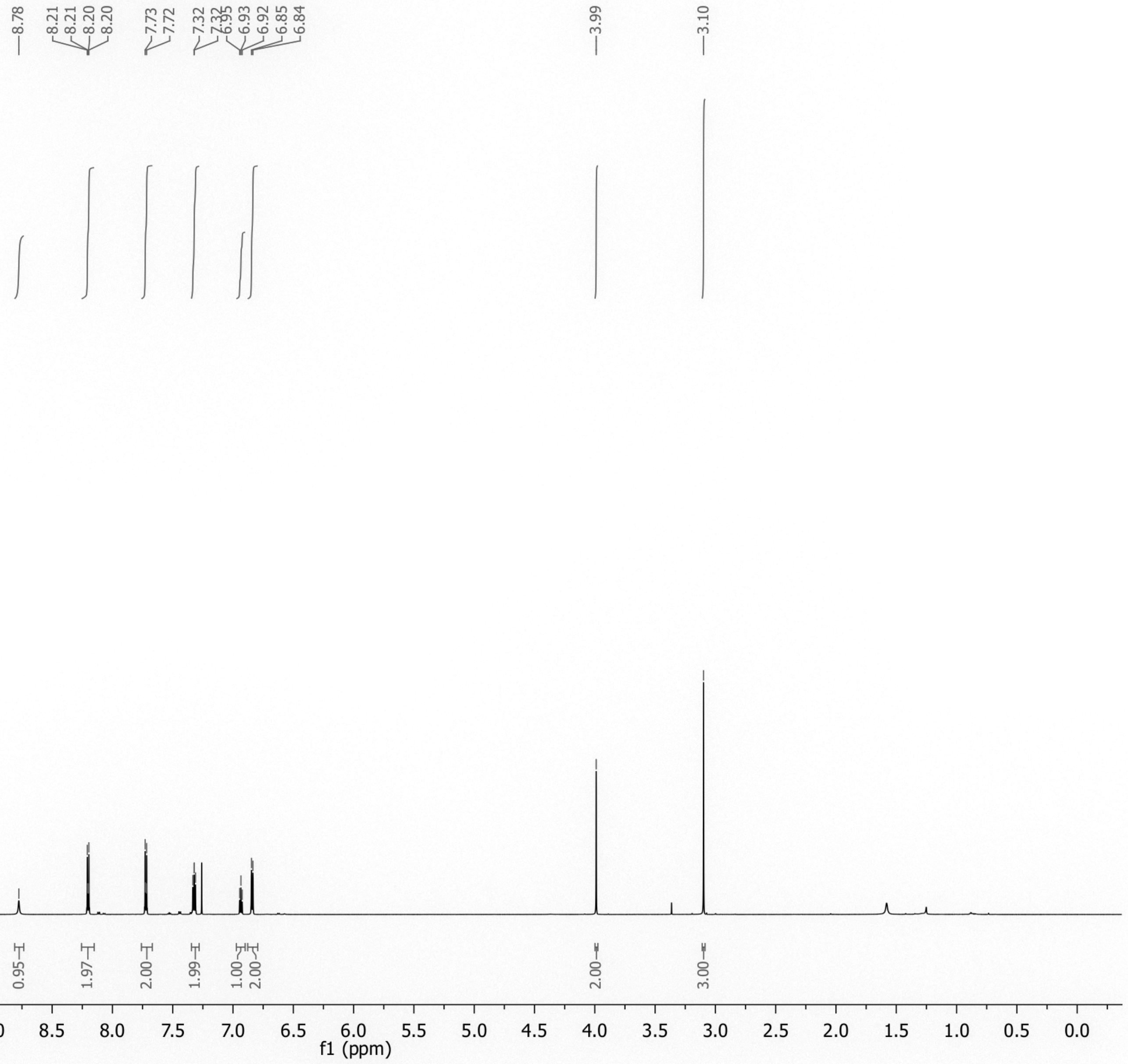

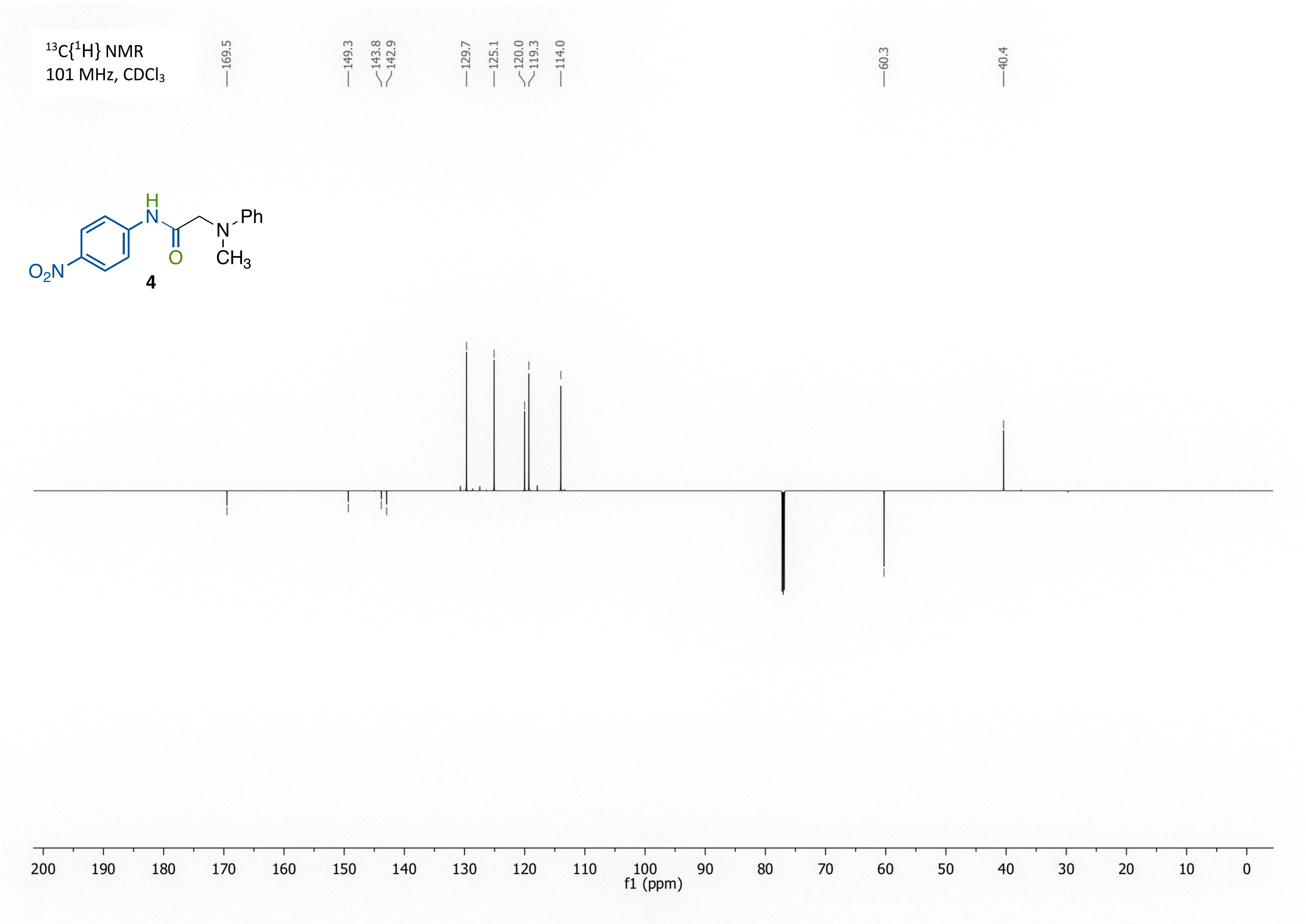

$^{13}C\{^{1}H\}$ NMR
101 MHz, $CDCl_3$
169.5
149.3
143.8
142.9
129.7
125.1
120.0
119.3
114.0
60.3
40.4
H
N
Ph
N
O
CH_3
O_2N
4
200
190
180
170
160
150
140
130
120
110
100
90
80
70
60
50
40
30
20
10
0
f1 (ppm)

^{1}H NMR
(700 MHz, $CDCl_3$)

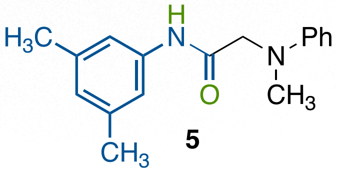

5

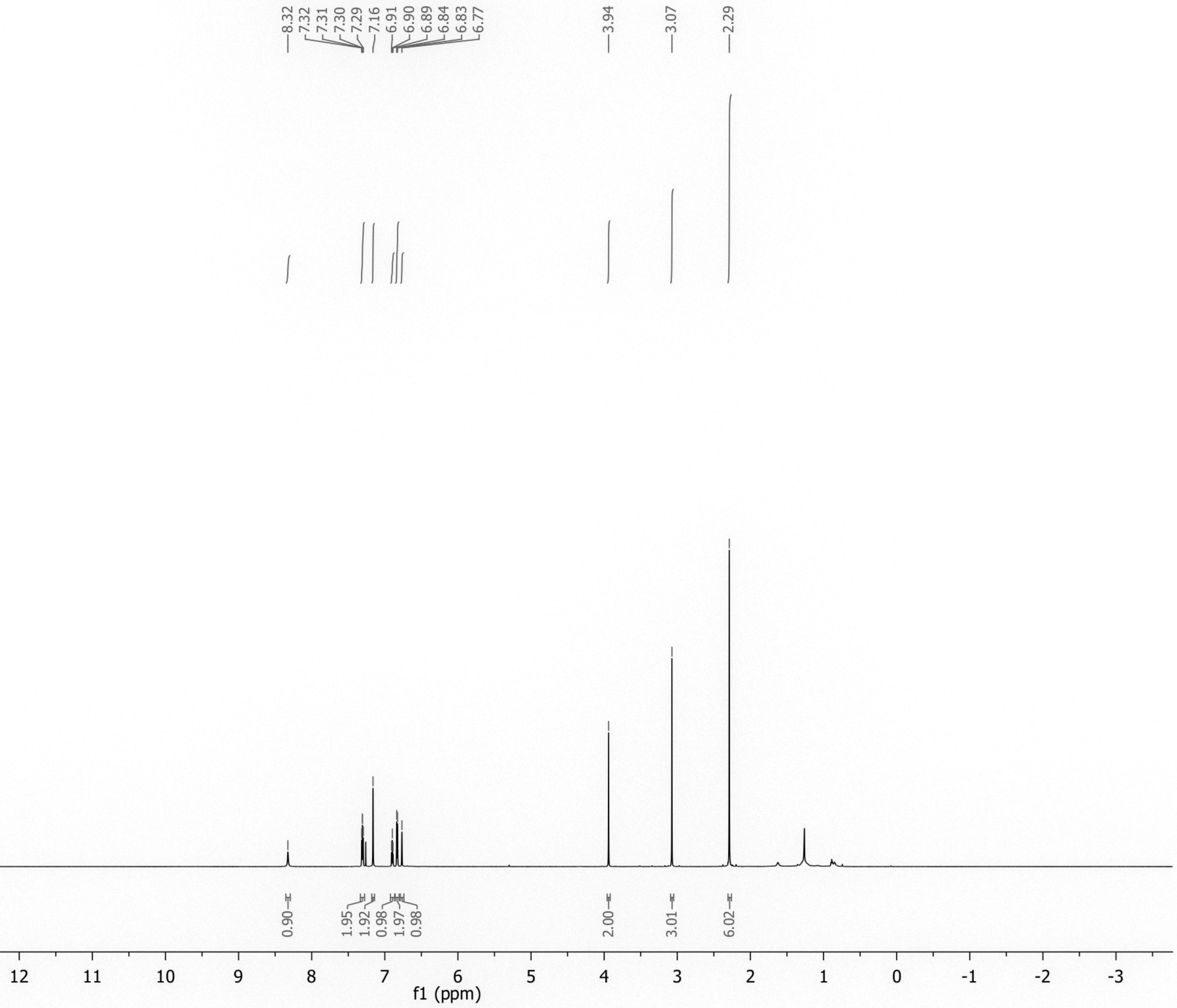

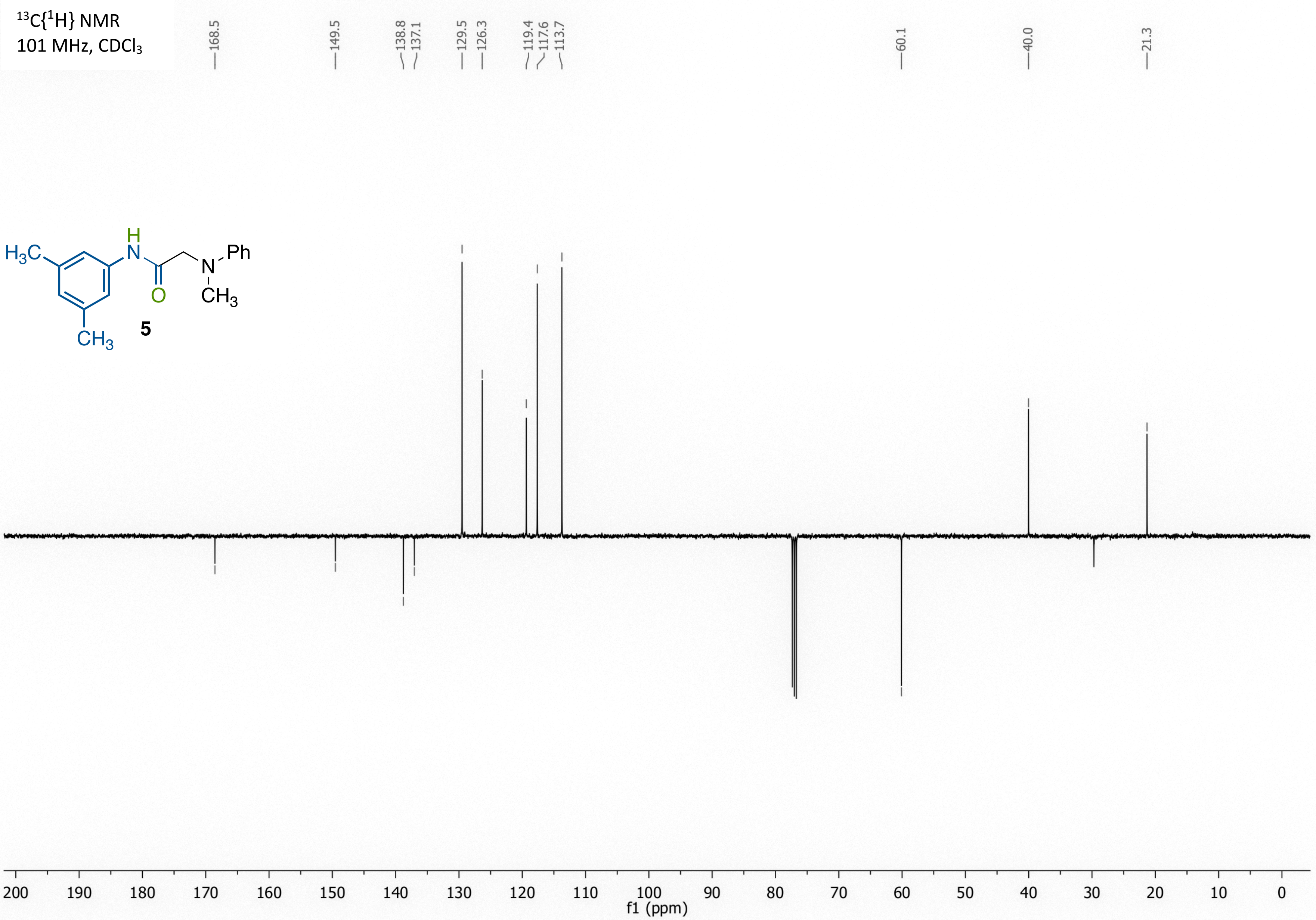
13C{1H} NMR
101 MHz, CDCl3
168.5
149.5
138.8
137.1
129.5
126.3
119.4
117.6
113.7
60.1
40.0
21.3
H3C
H
N
O
N
Ph
CH3
CH3
5
200 190 180 170 160 150 140 130 120 110 100 90 80 70 60 50 40 30 20 10 0
f1 (ppm)

^{1}H NMR
(700 MHz, $CDCl_3$)

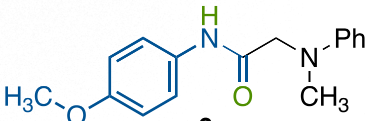

6

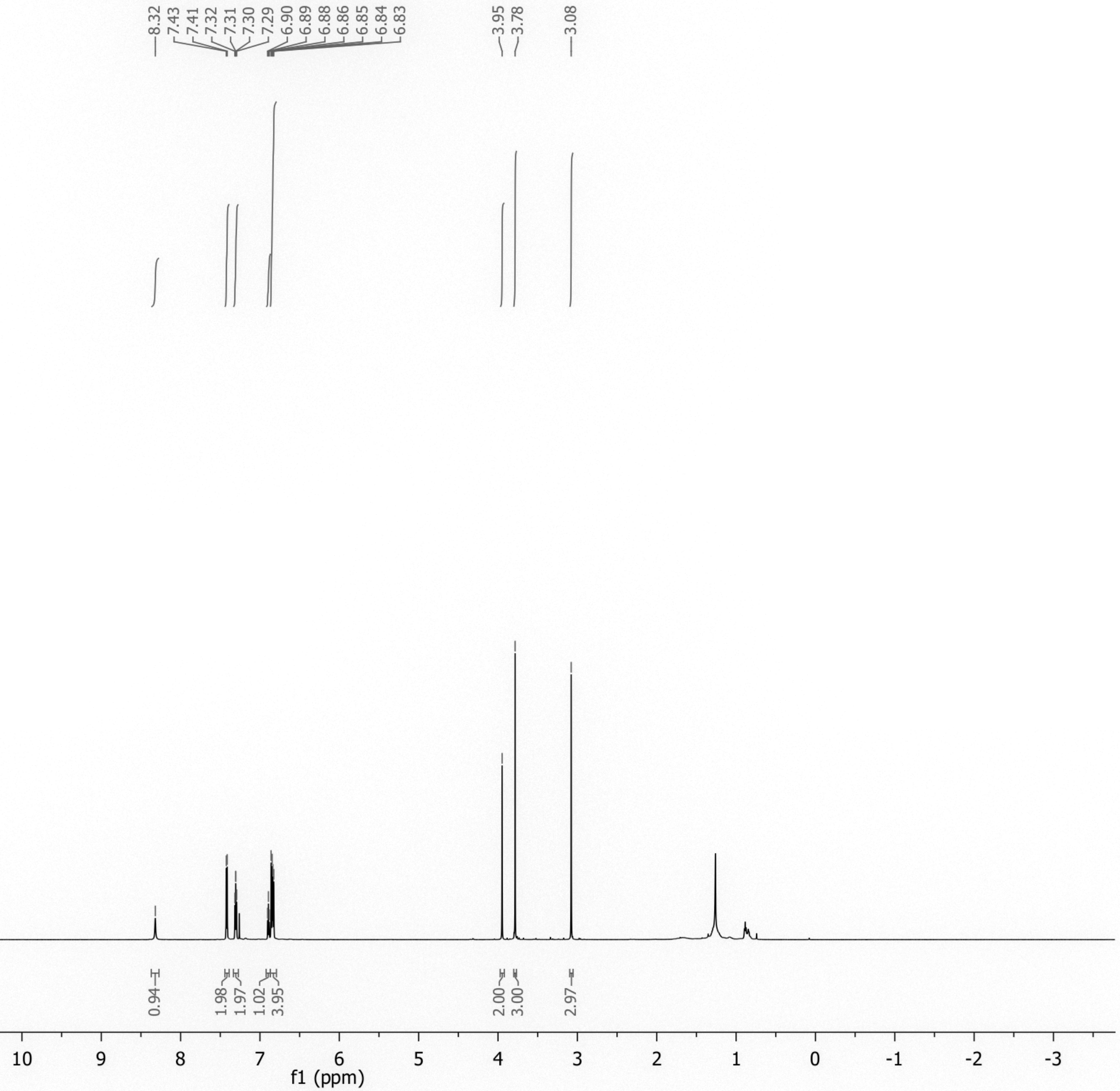

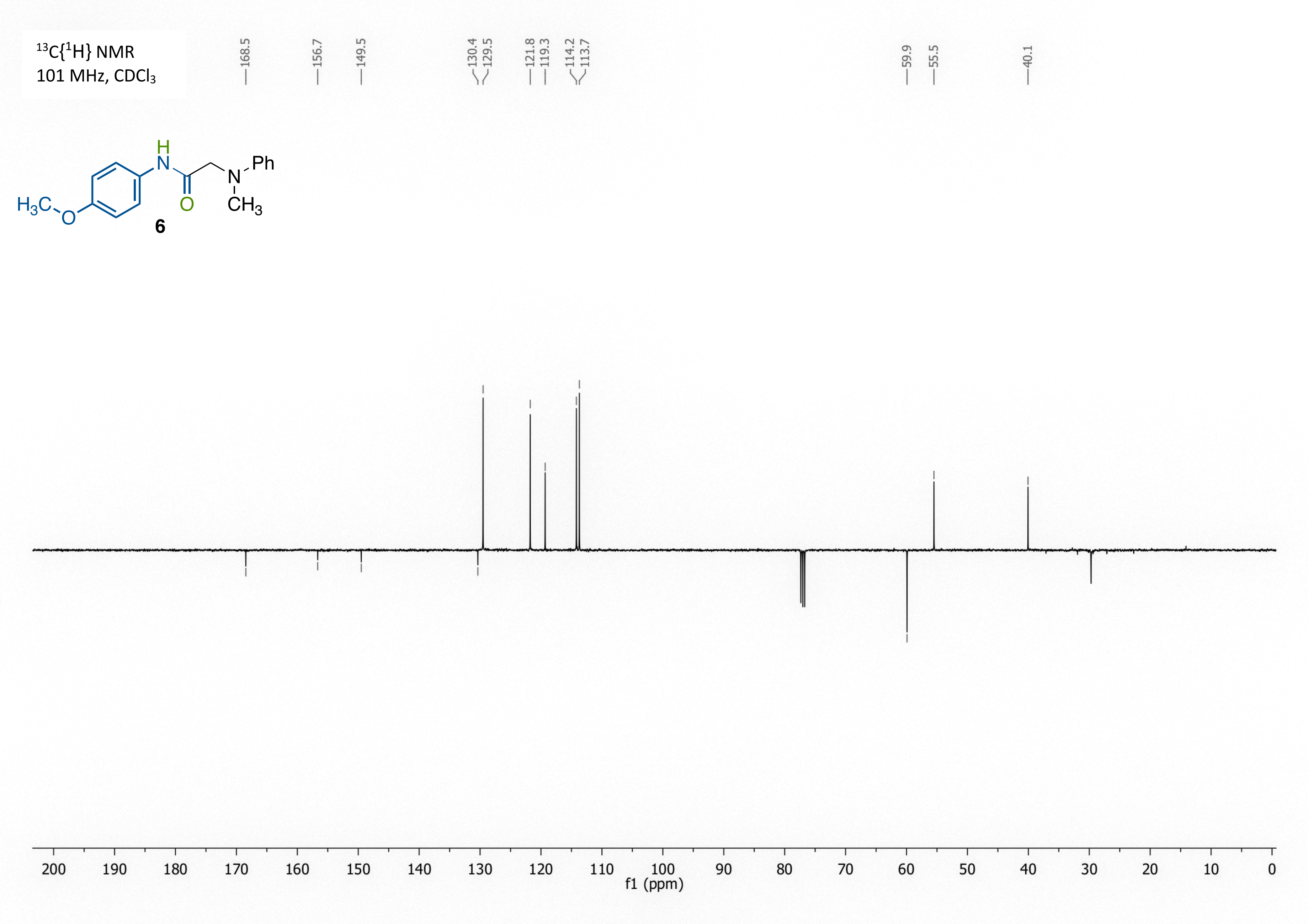

$^{13}C\{^1H\}$ NMR
101 MHz, $CDCl_3$
168.5
156.7
149.5
130.4
129.5
121.8
119.3
114.2
113.7
59.9
55.5
40.1
6
200 190 180 170 160 150 140 130 120 110 100 90 80 70 60 50 40 30 20 10 0
f1 (ppm)

^{1}H NMR
(400 MHz, $CDCl_3$)

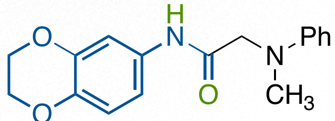

7

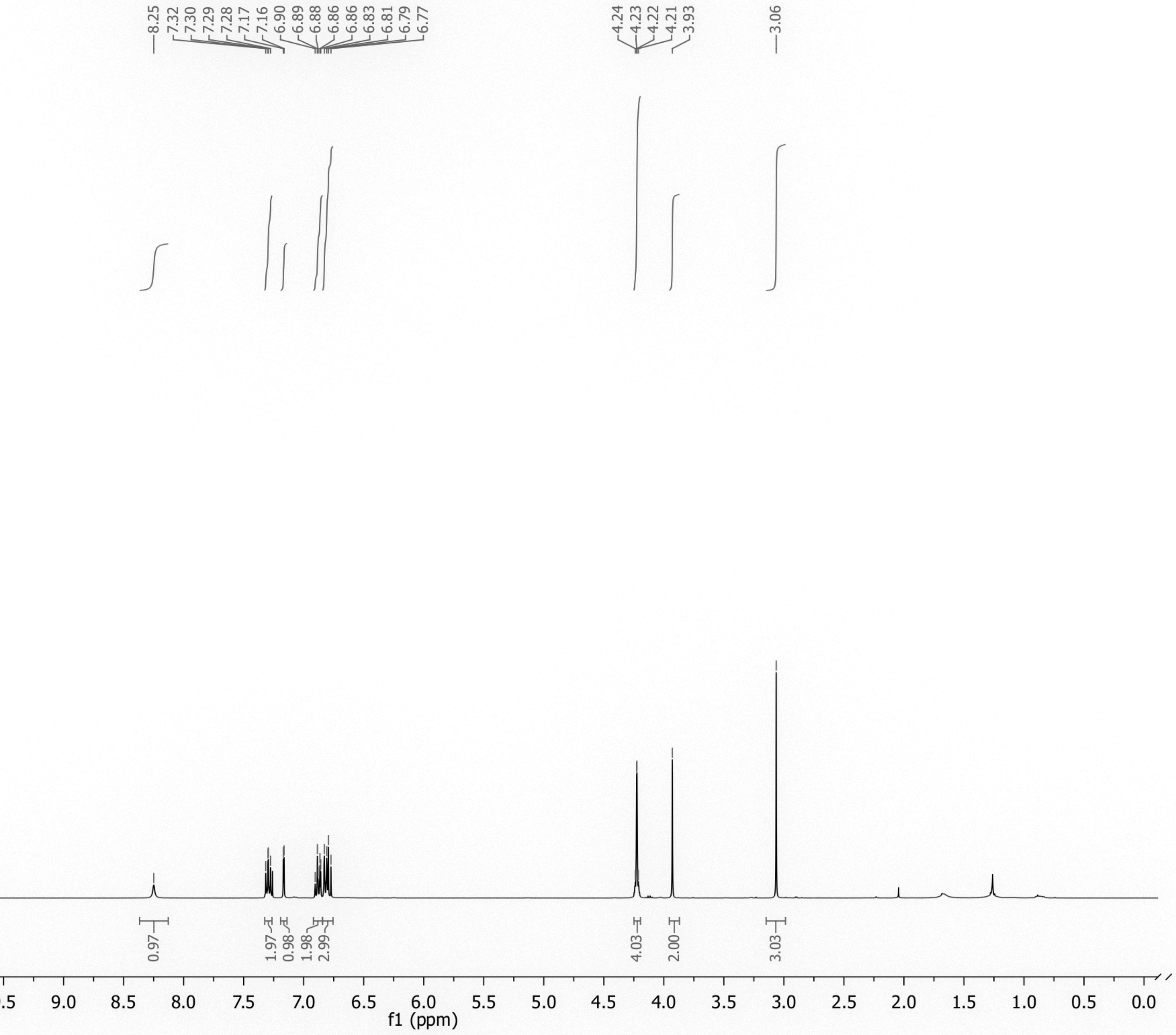

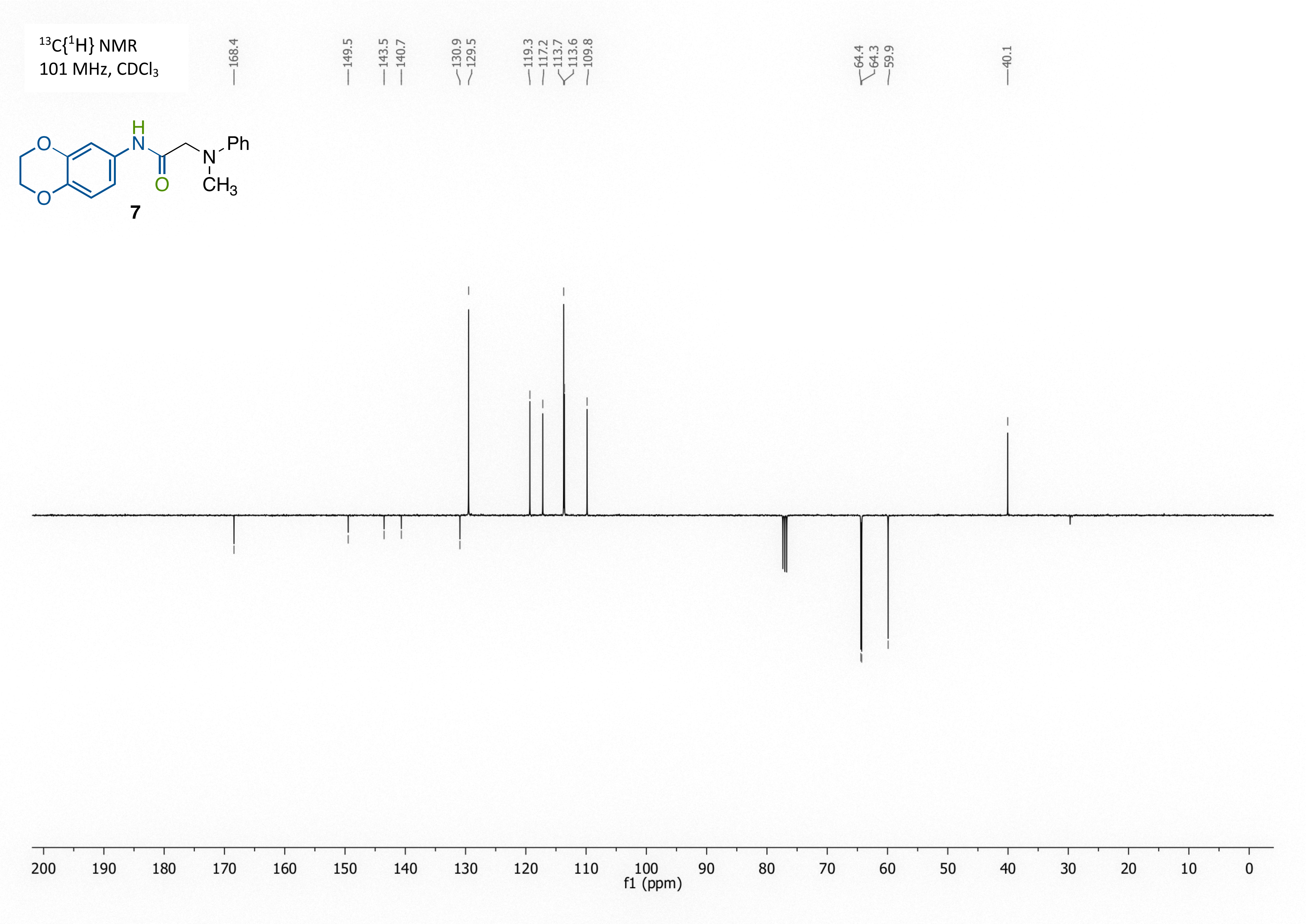

13C{1H} NMR
101 MHz, CDCl3
168.4
149.5
143.5
140.7
130.9
129.5
119.3
117.2
113.7
113.6
109.8
64.4
64.3
59.9
40.1
H
N
O
O
O
N
Ph
CH3
7
200 190 180 170 160 150 140 130 120 110 100 90 80 70 60 50 40 30 20 10 0
f1 (ppm)

^{1}H NMR
(400 MHz, $CDCl_3$)

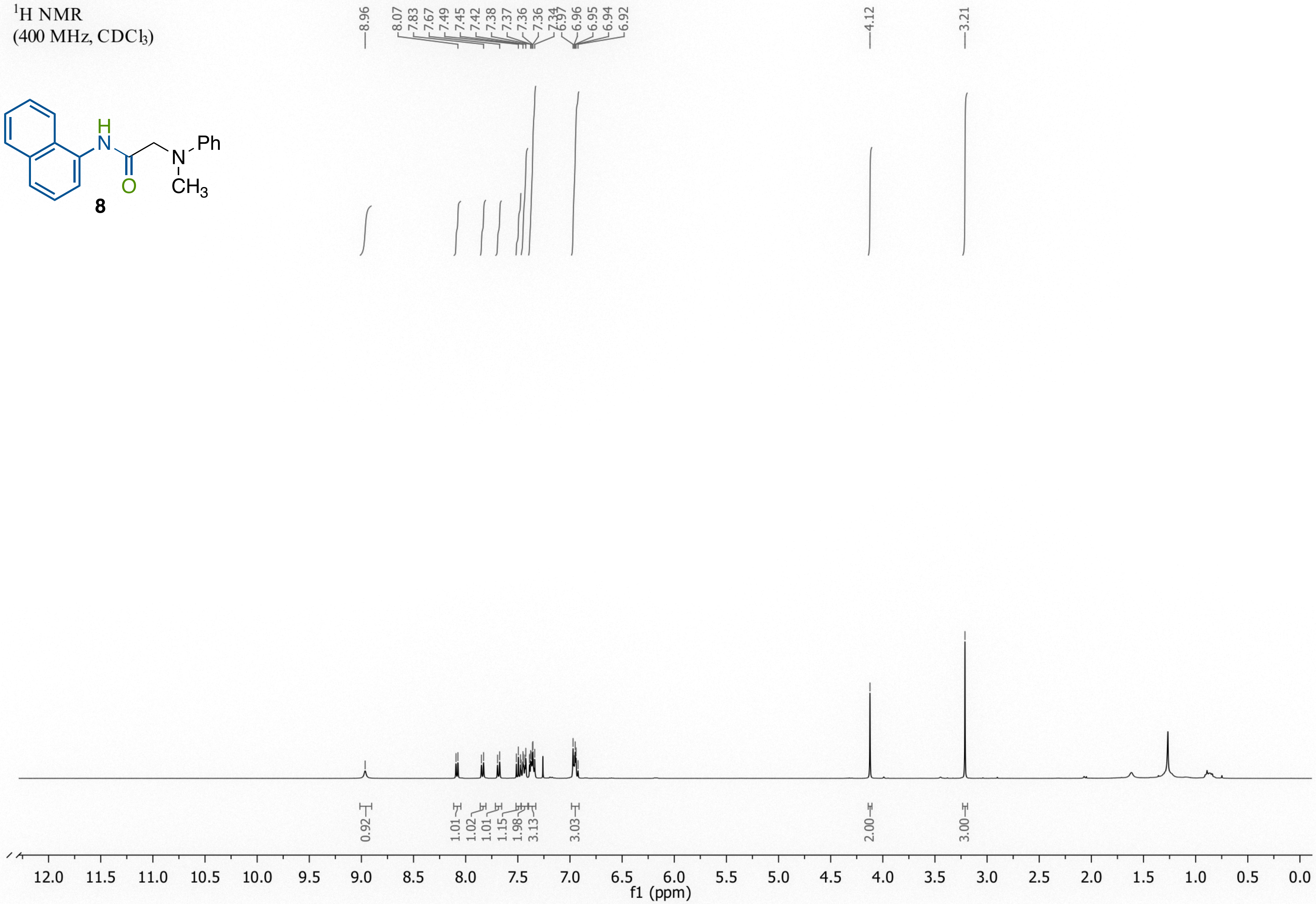

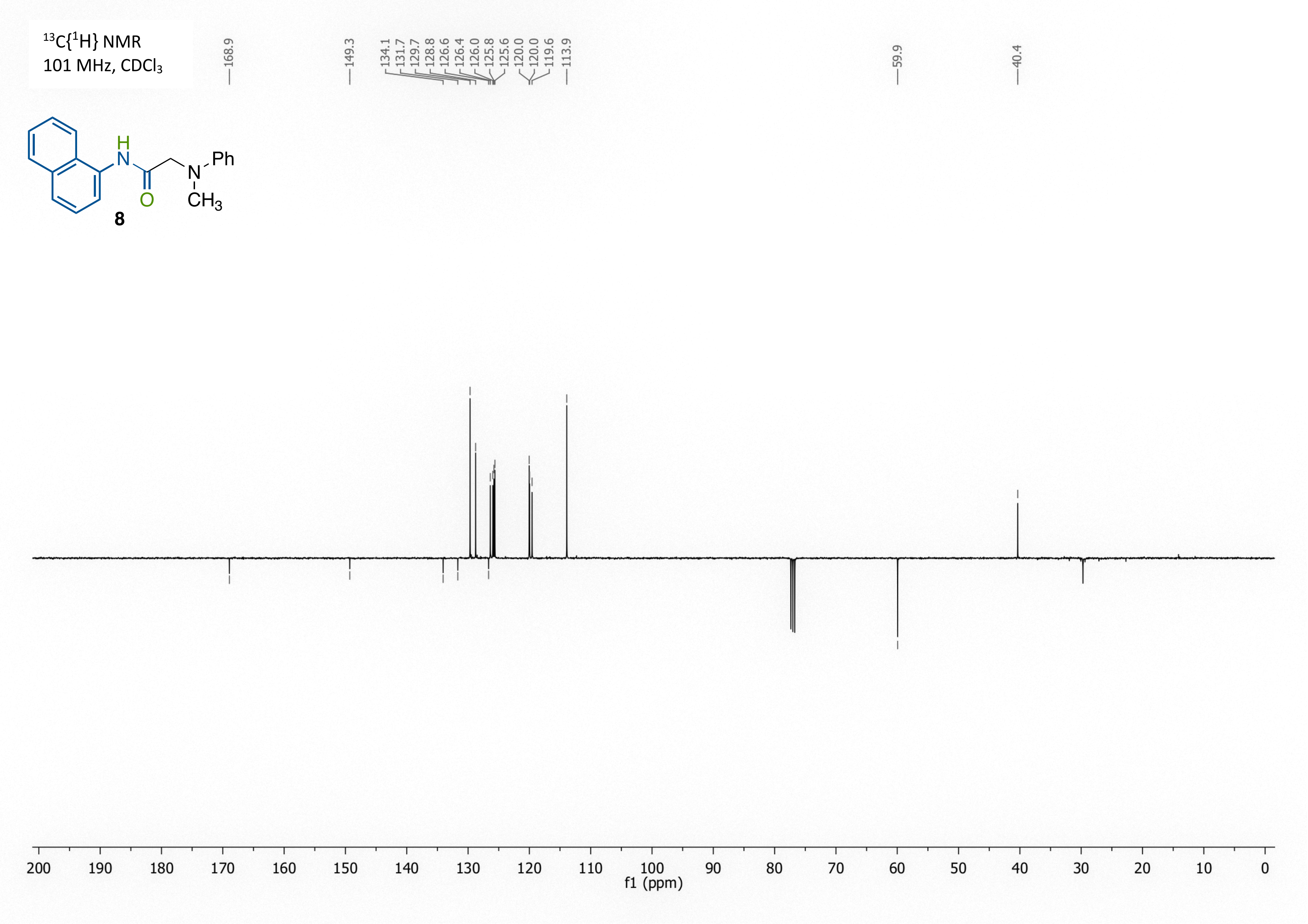
13C{1H} NMR
101 MHz, CDCl3
168.9
149.3
134.1
131.7
129.7
128.8
126.6
126.4
126.0
125.8
125.6
120.0
120.0
119.6
113.9
59.9
40.4
H
N
O
N
Ph
CH3
8
200 190 180 170 160 150 140 130 120 110 100 90 80 70 60 50 40 30 20 10 0
f1 (ppm)

^1H NMR
400 MHz, $CDCl_3$

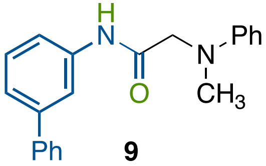

9

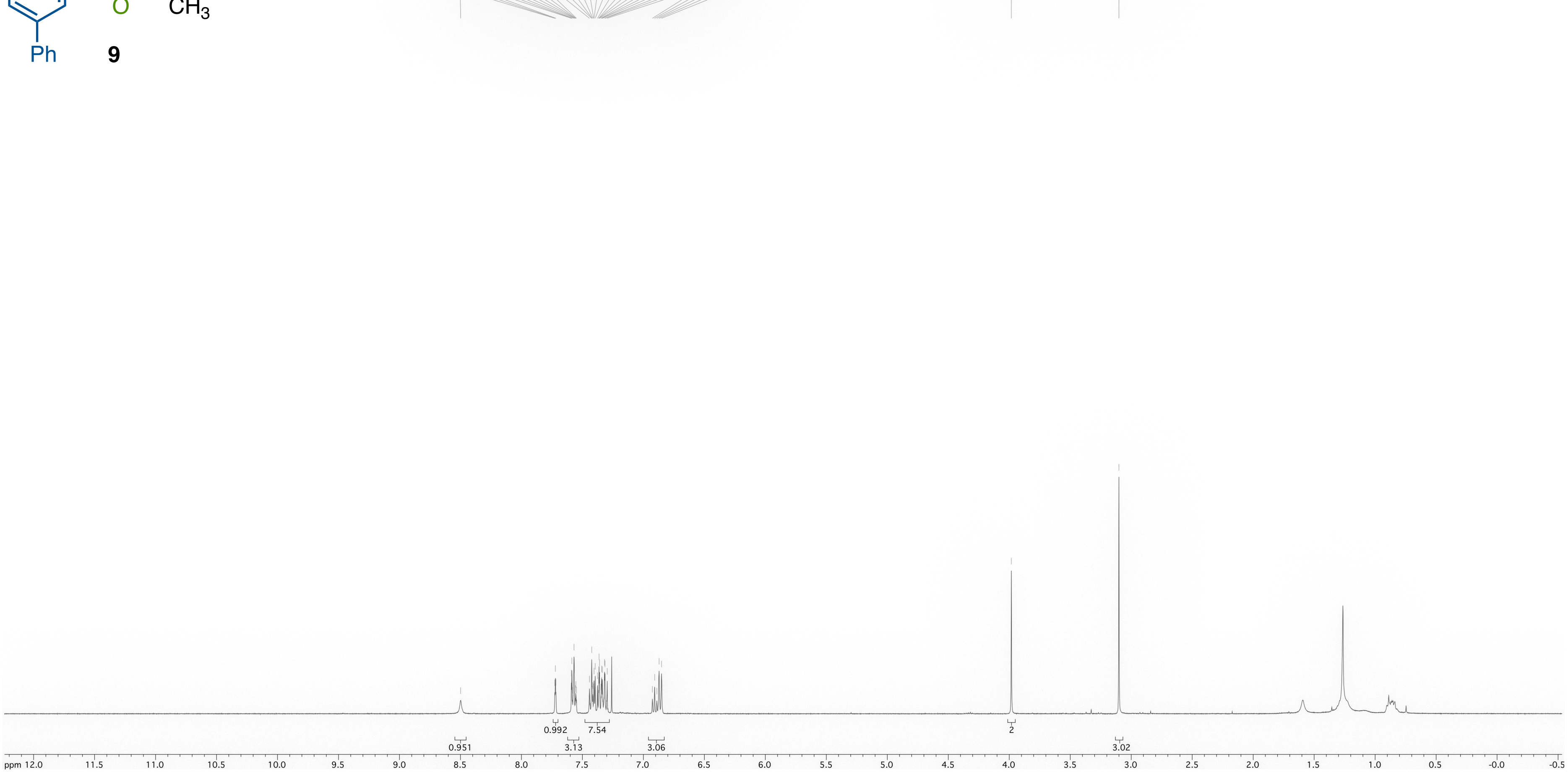

$^{13}C\{^1H\}$ NMR
101 MHz, $CDCl_3$

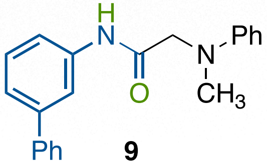

9

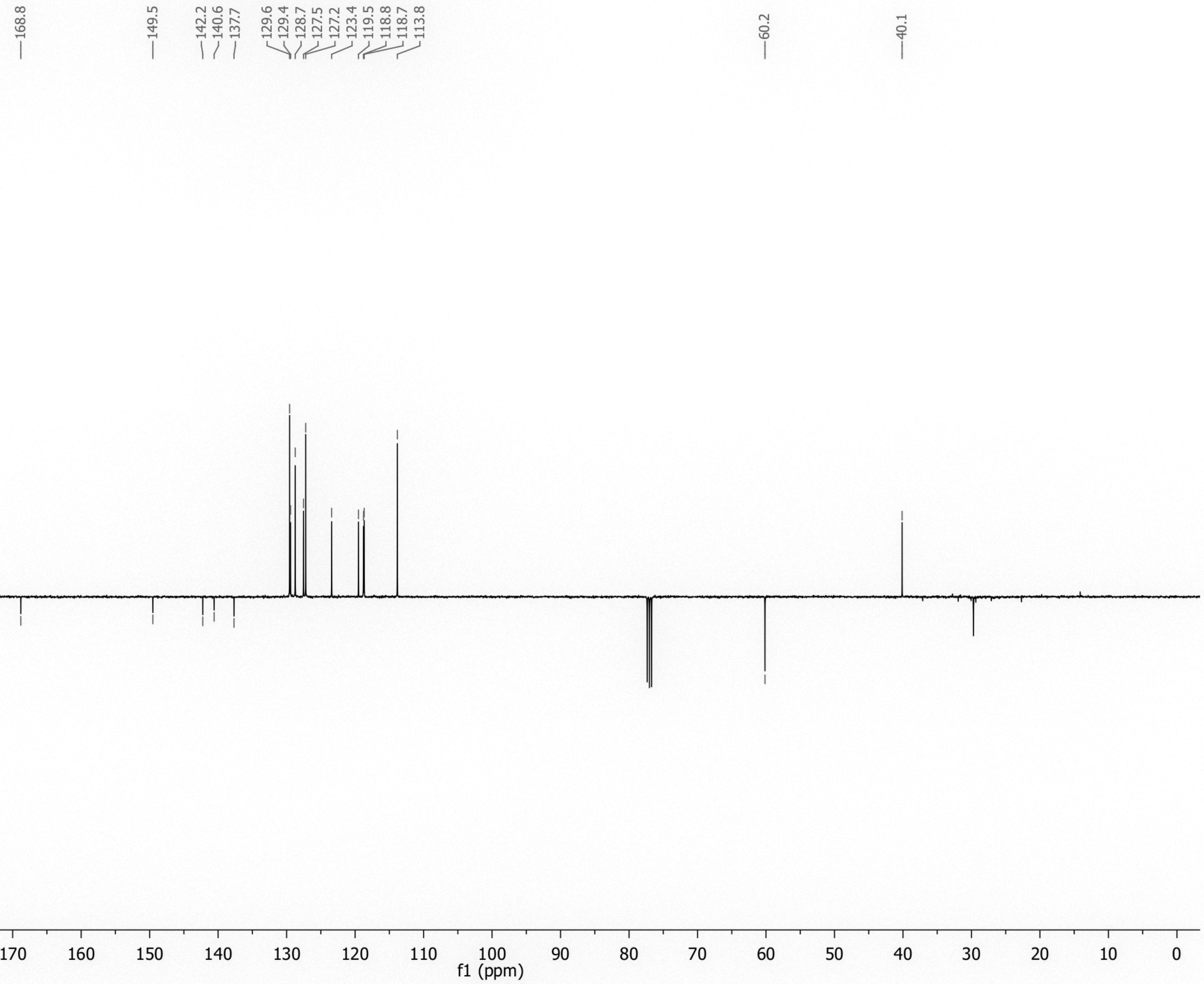

^1H NMR
(400 MHz, $CDCl_3$)

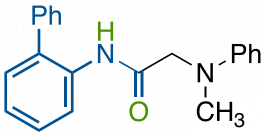

10

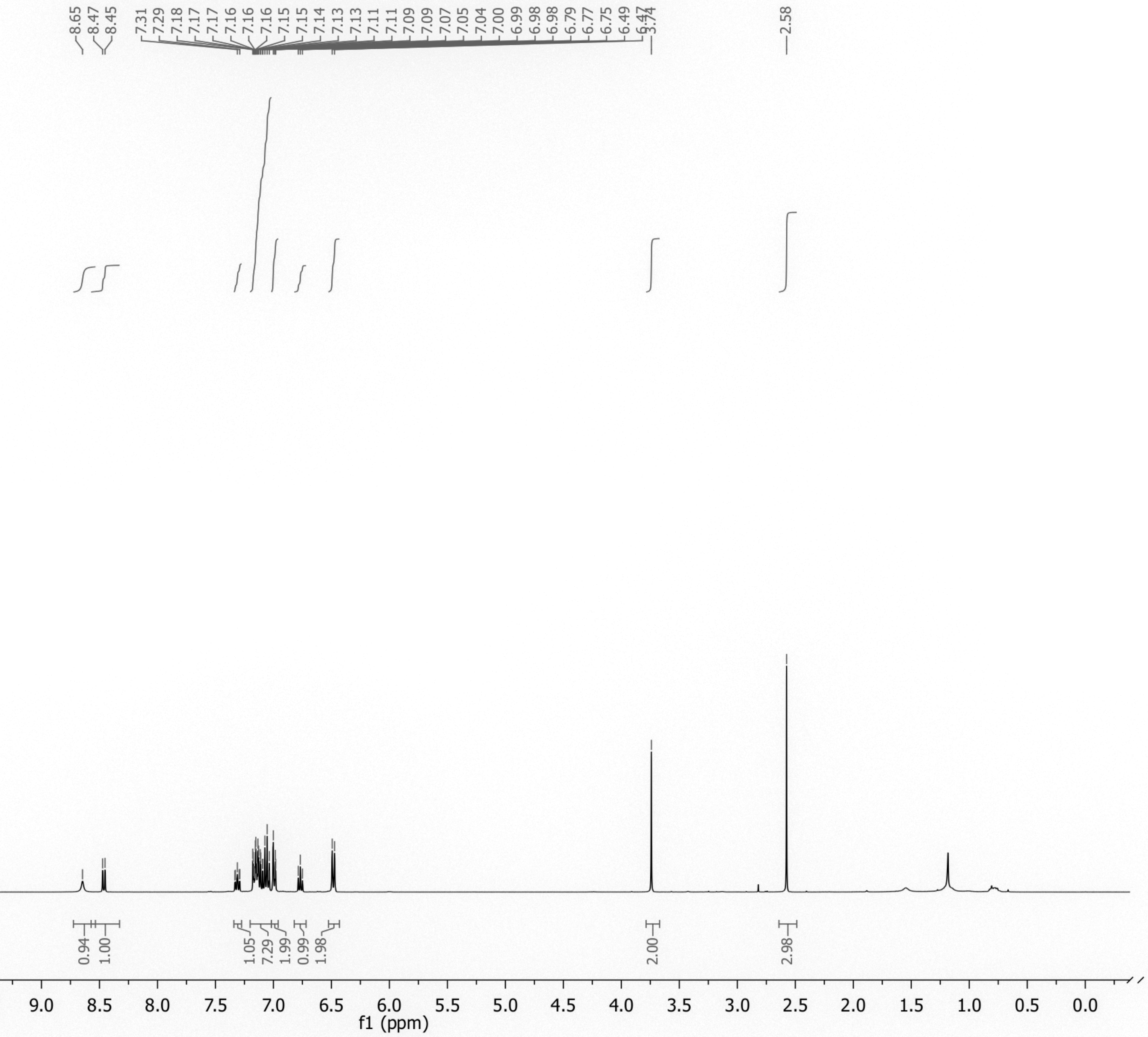

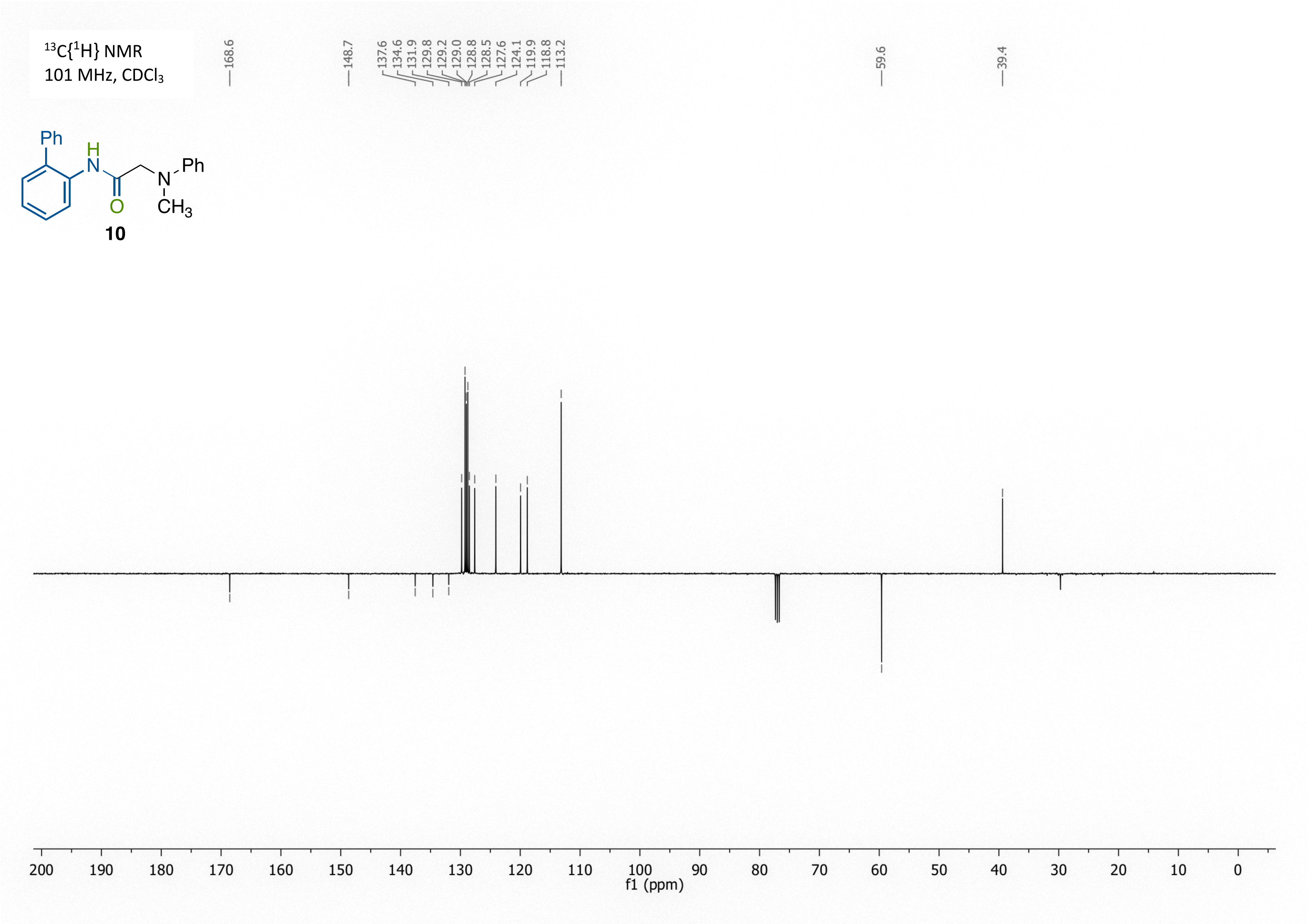
$^{13}C\{^1H\}$ NMR
101 MHz, $CDCl_3$
168.6
148.7
137.6
134.6
131.9
129.8
129.2
129.0
128.8
128.5
127.6
124.1
119.9
118.8
113.2
59.6
39.4
Ph
H
N
Ph
N
O
CH3
f1 (ppm)

10

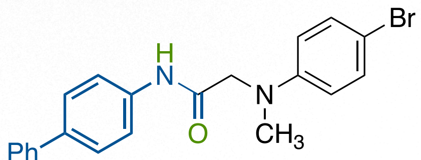

11

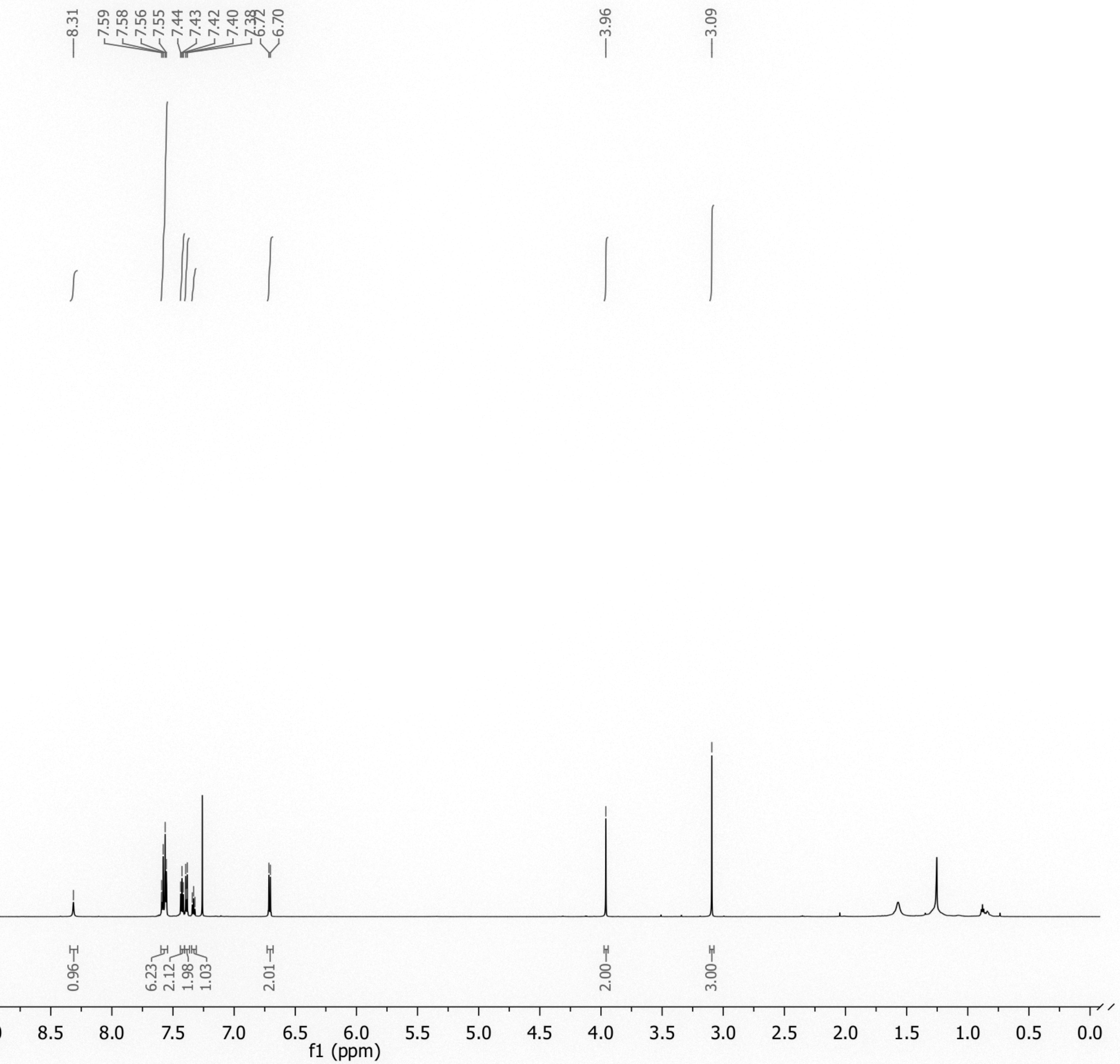

^{1}H NMR
(700 MHz, $CDCl_3$)
8.31
7.59
7.58
7.56
7.55
7.44
7.43
7.42
7.40
7.38
6.72
6.70
3.96
3.09
0.96
6.23
2.12
1.98
1.03
2.01
2.00
3.00
12.0
11.5
11.0
10.5
10.0
9.5
9.0
8.5
8.0
7.5
7.0
6.5
6.0
5.5
5.0
4.5
4.0
3.5
3.0
2.5
2.0
1.5
1.0
0.5
0.0
f1 (ppm)

$^{13}C\{^1H\}$ NMR
176 MHz, $CDCl_3$

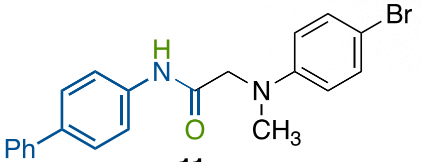

11

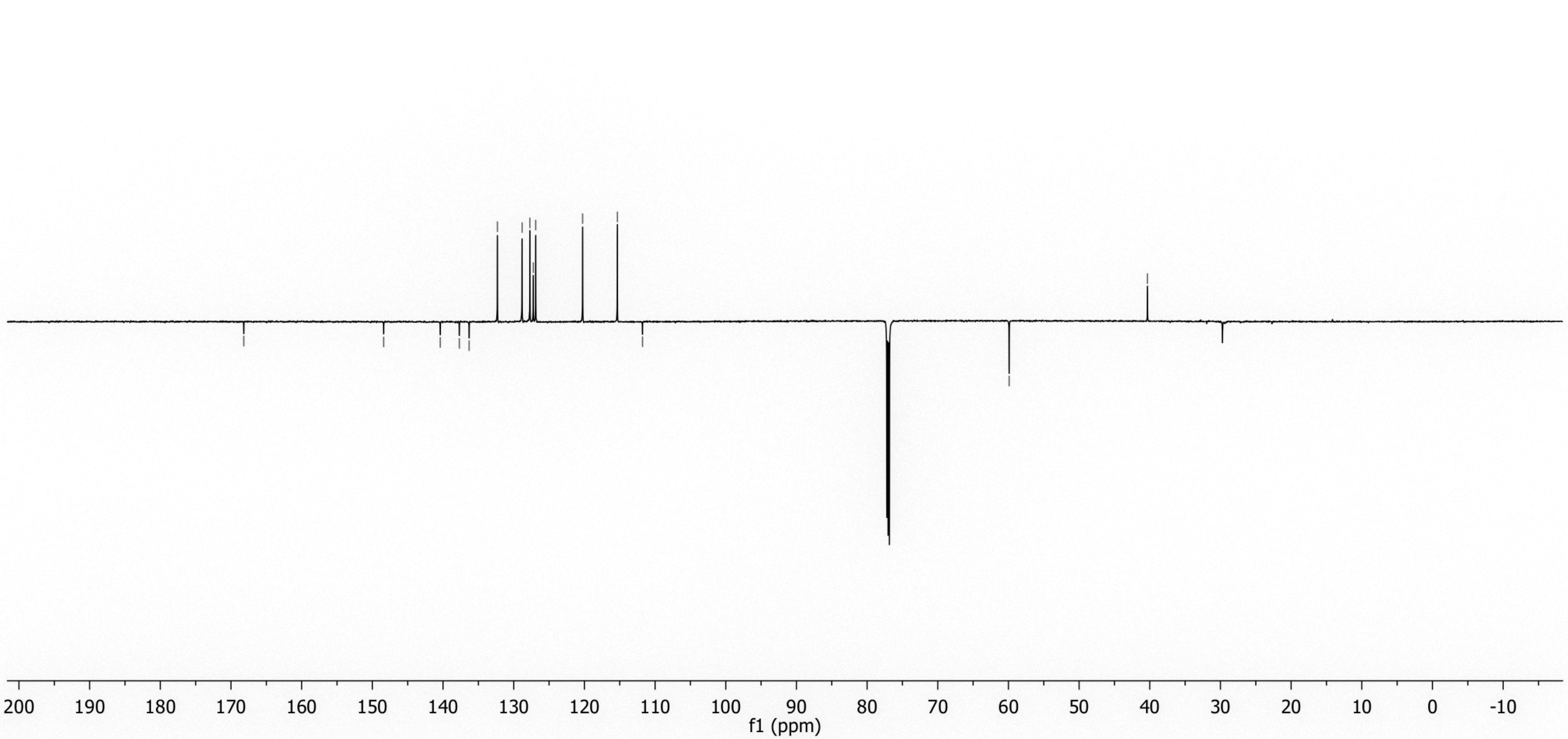

^{1}H NMR
700 MHz, $CDCl_3$

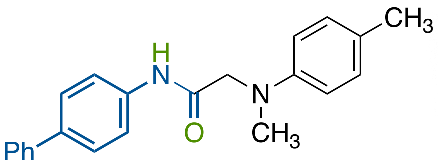

12

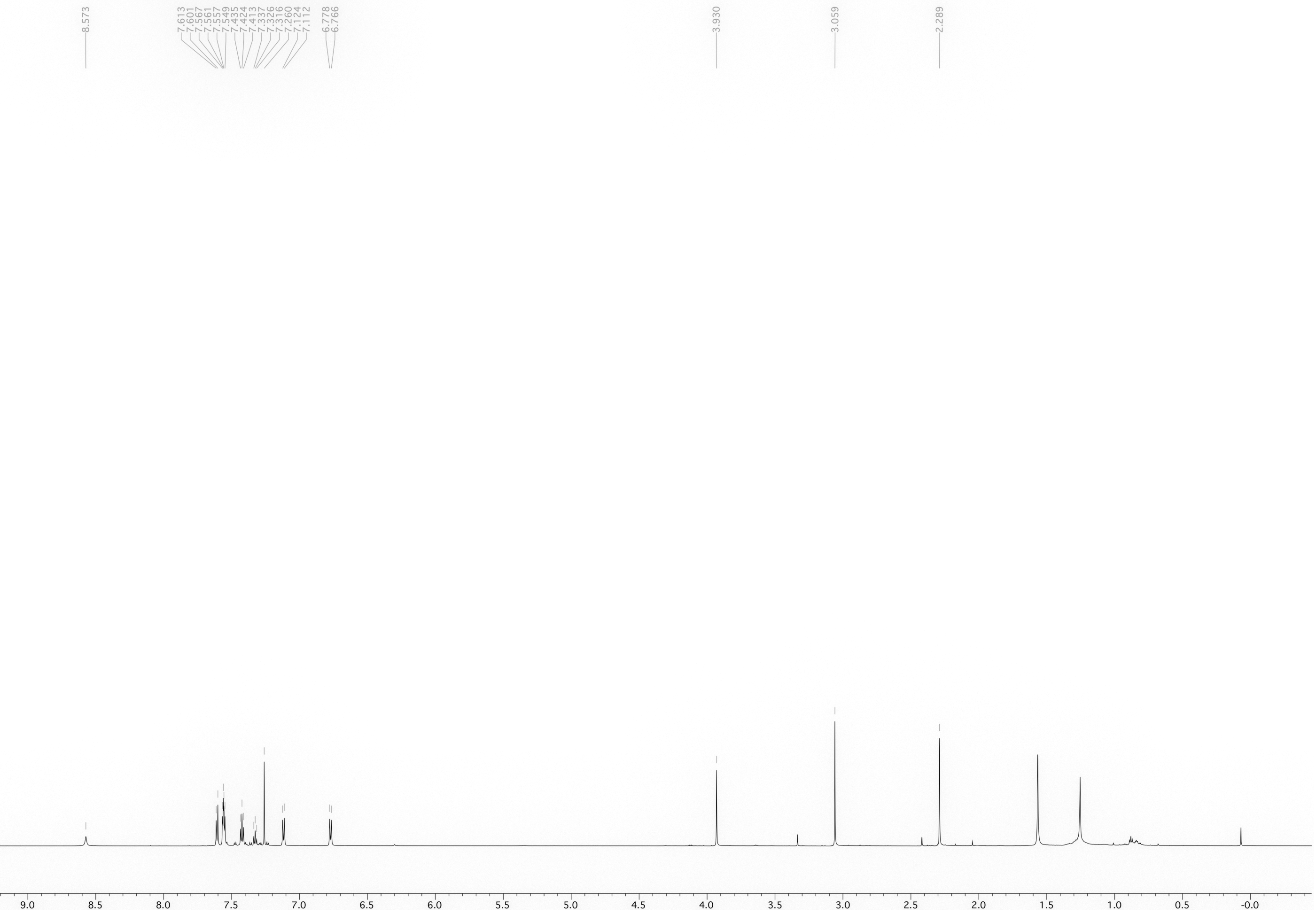

$^{13}C\{^1H\}$ NMR
176 MHz, $CDCl_3$

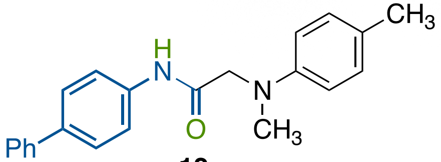

12

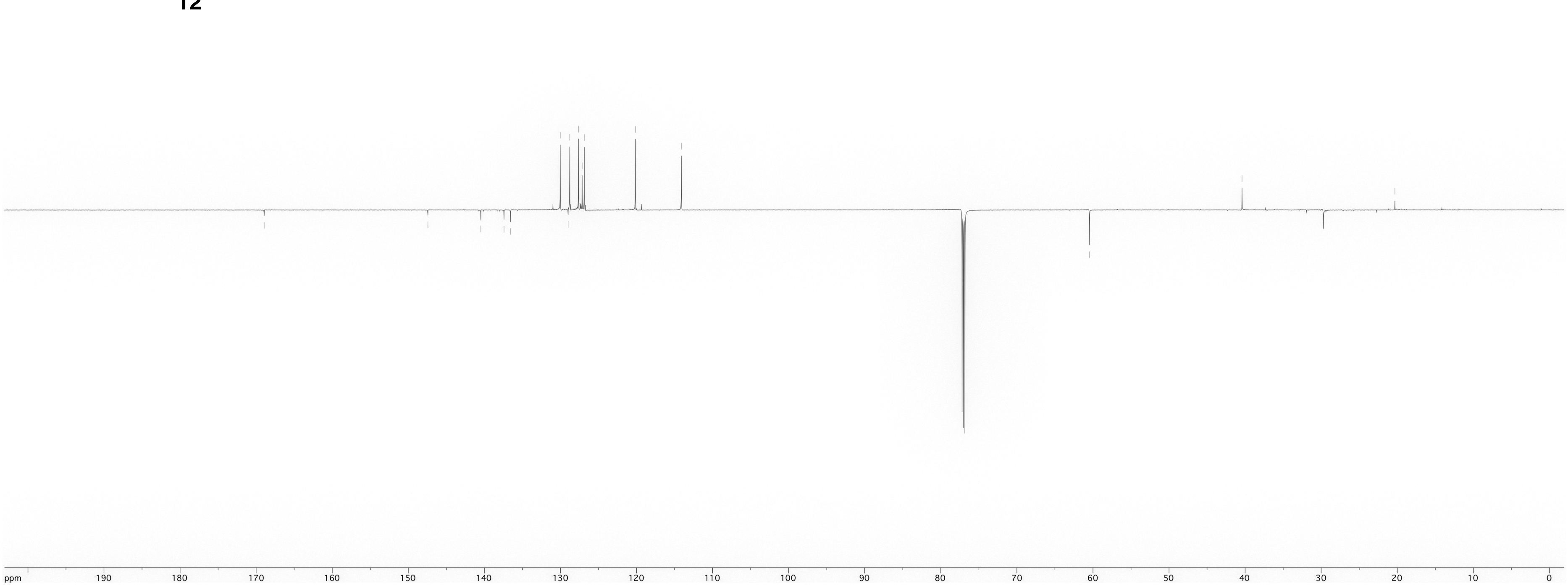

^{1}H NMR
700 MHz, $CDCl_3$

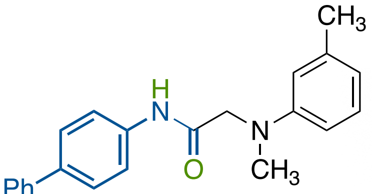

13

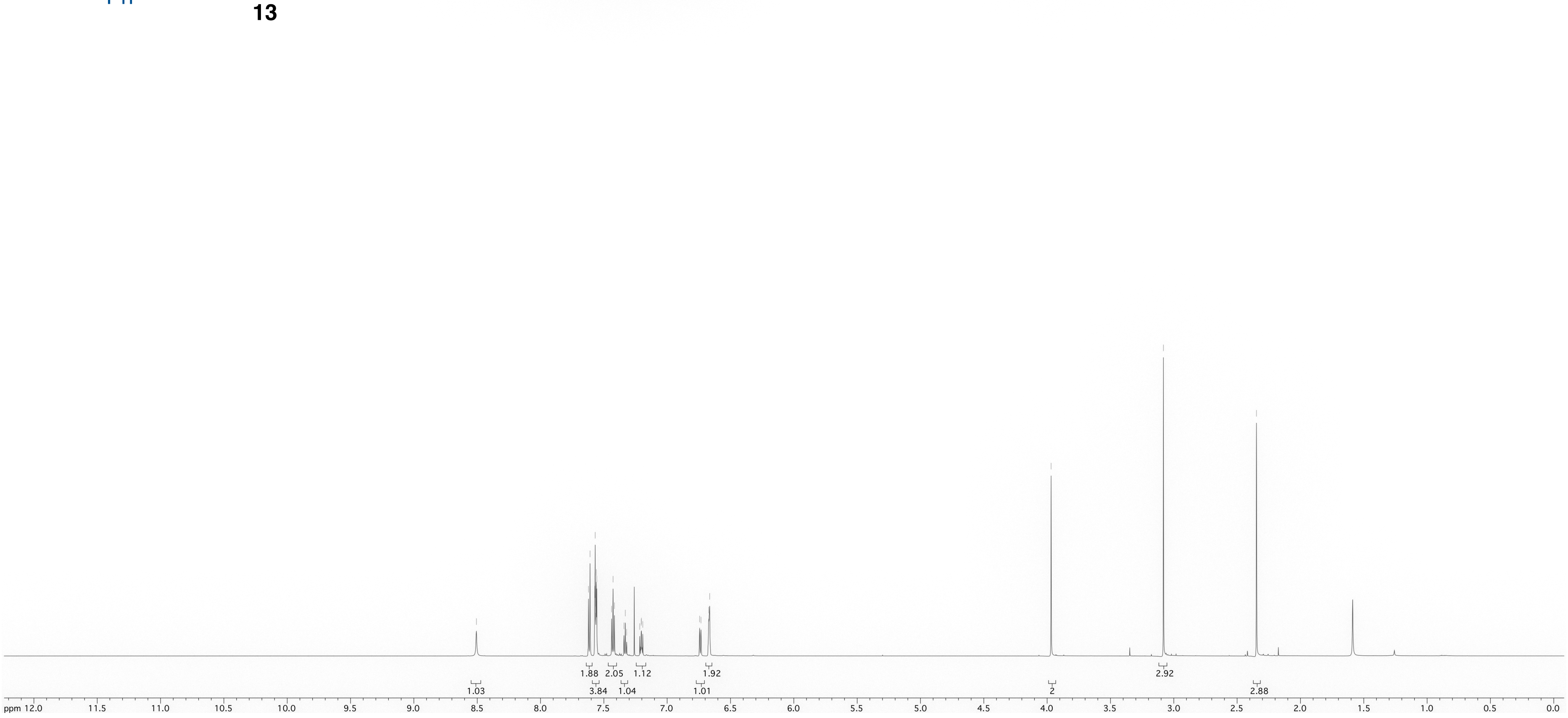

$^{13}C\{^1H\}$ NMR
176 MHz, $CDCl_3$

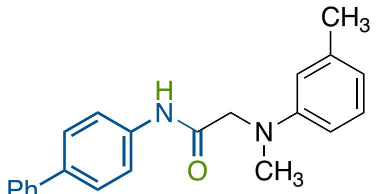

13

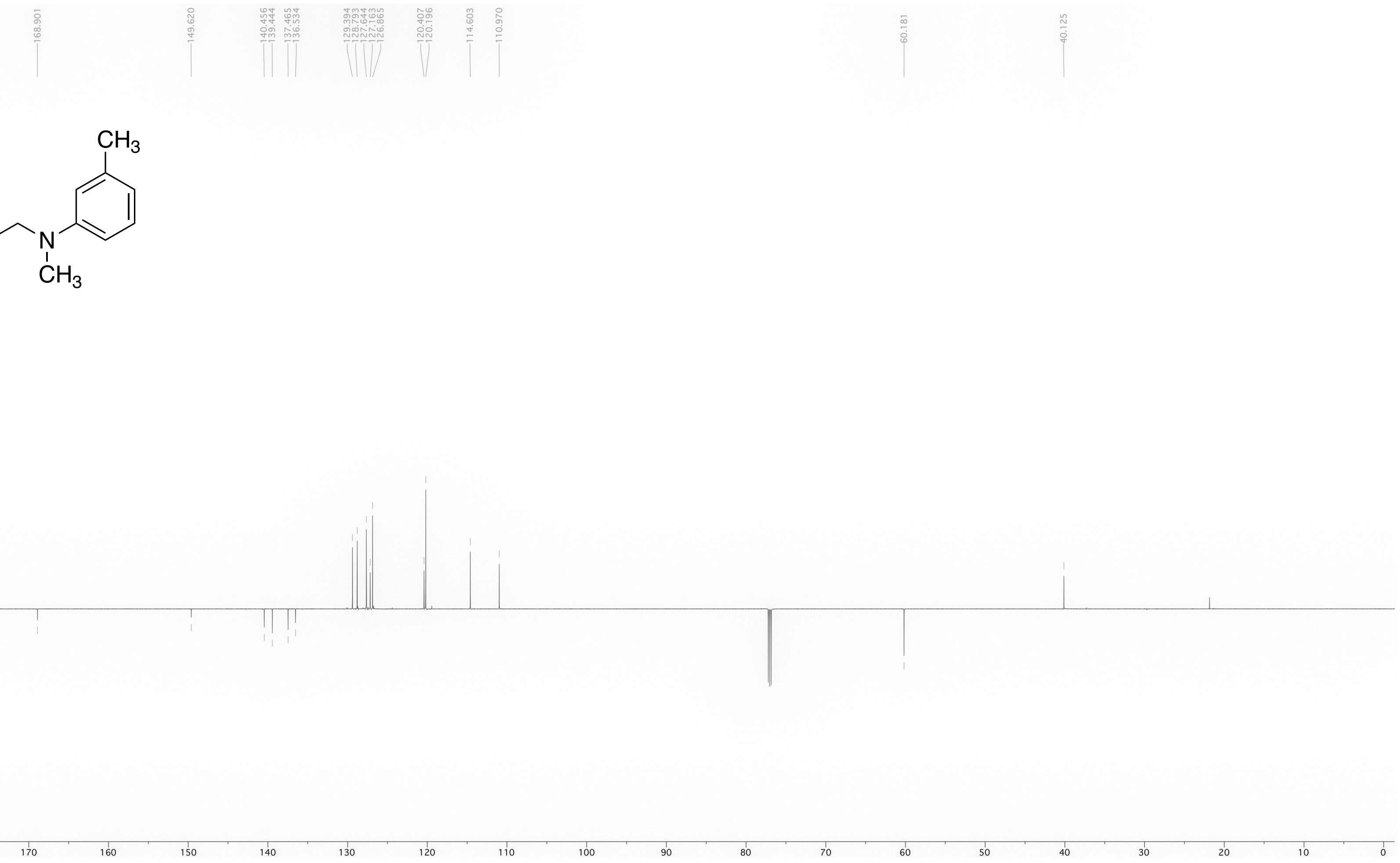

^{1}H NMR
700 MHz, $CDCl_3$

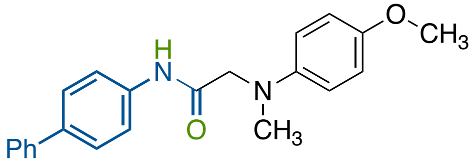

14

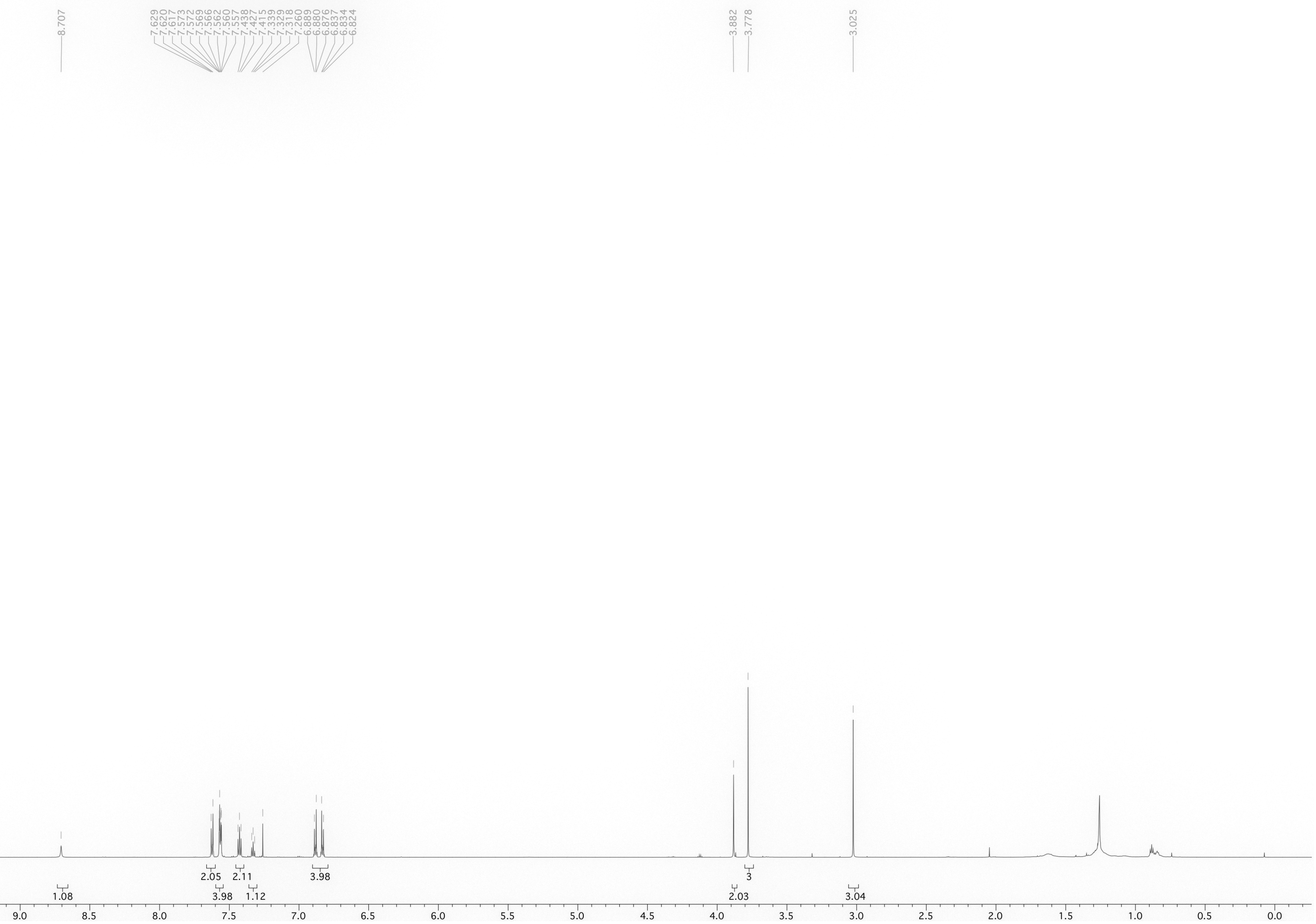

$^{13}C\{^1H\}$ NMR
176 MHz, $CDCl_3$

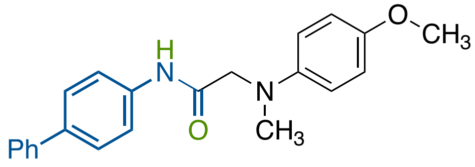

14

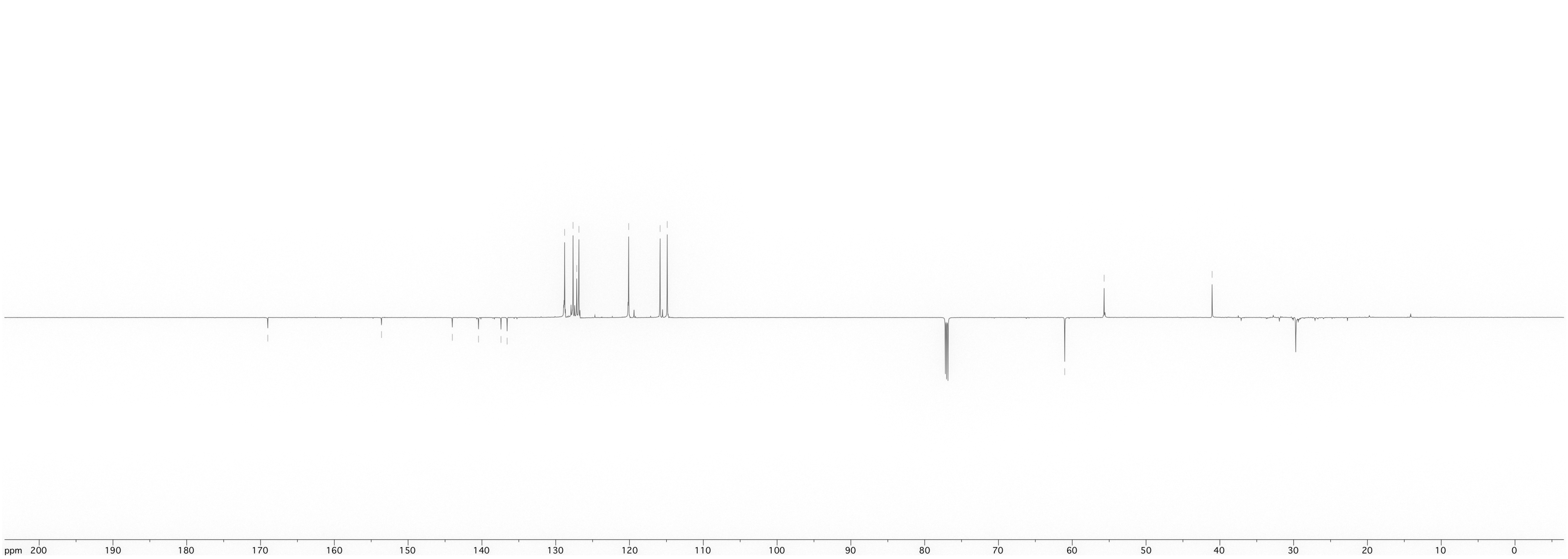

^{1}H NMR
(400 MHz, $CDCl_3$)

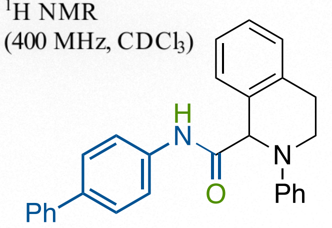

15

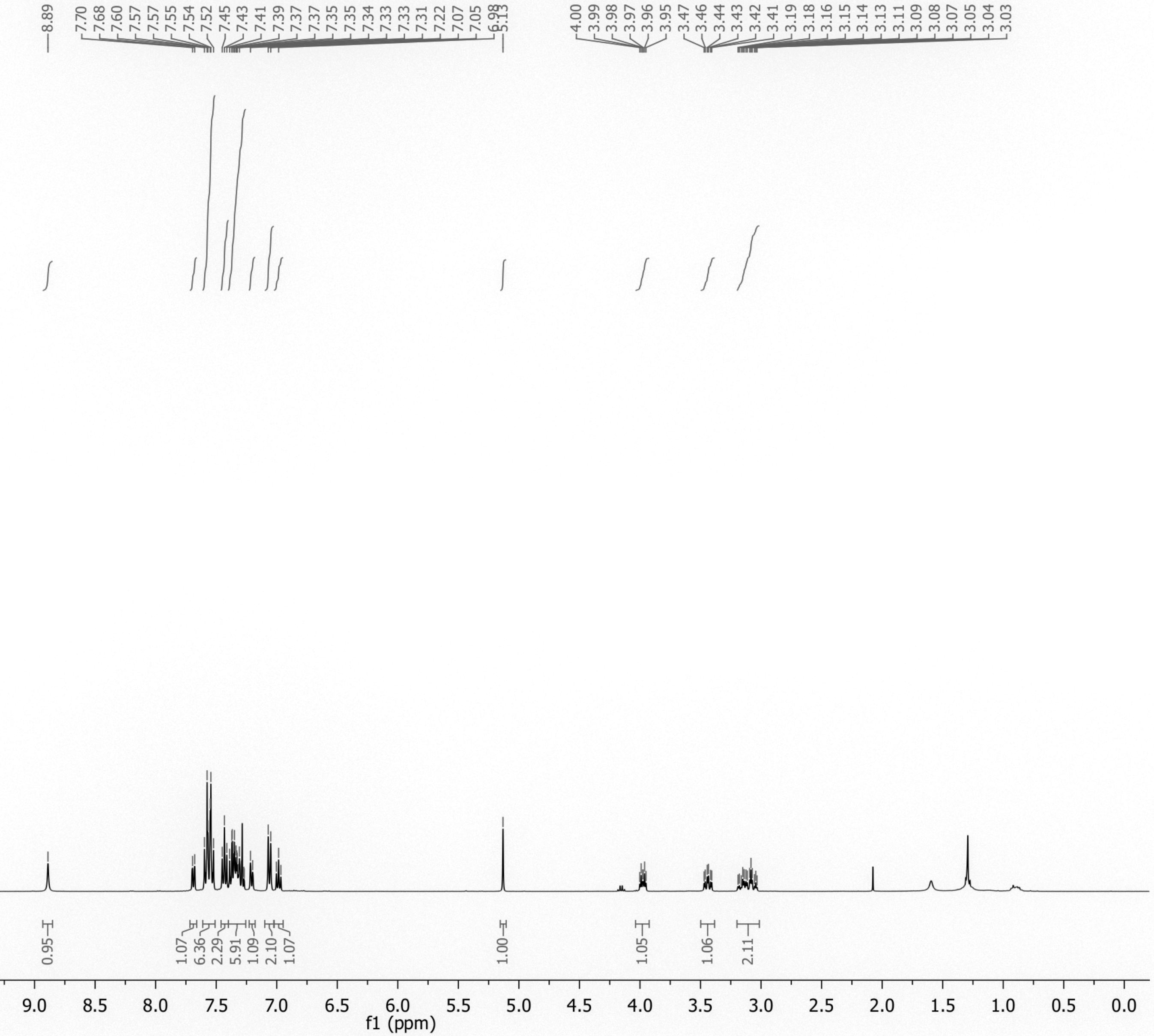

$^{13}C\{^{1}H\}$ NMR
101 MHz, $CDCl_3$

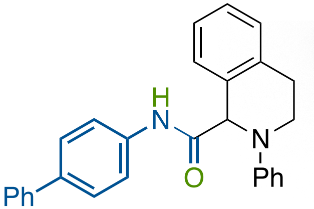

15

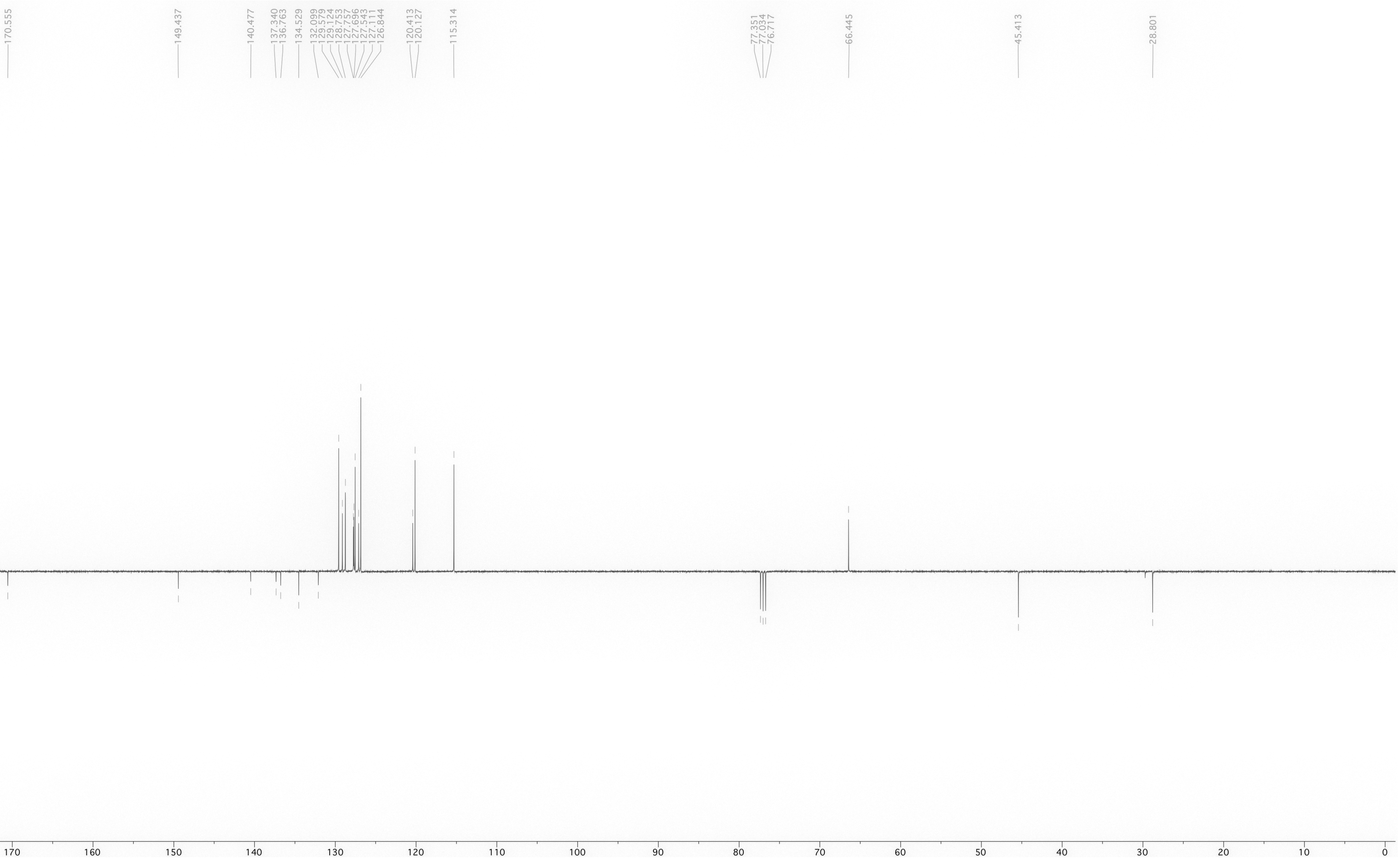

^{1}H NMR
700 MHz, $CDCl_3$

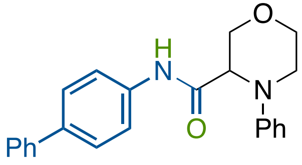

16

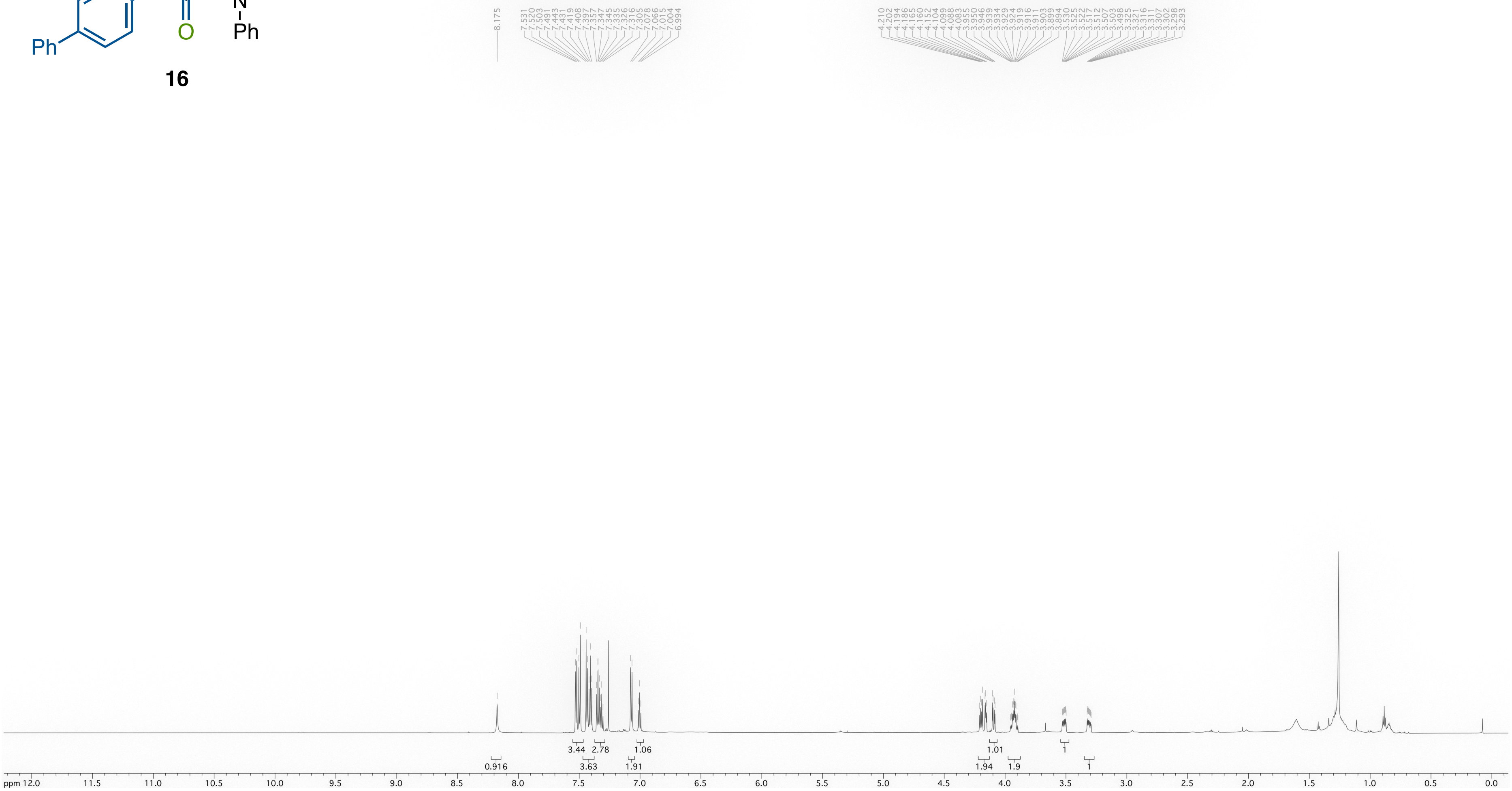

$^{13}C\{^{1}H\}$ NMR
176 MHz, $CDCl_3$

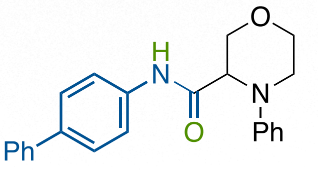

16

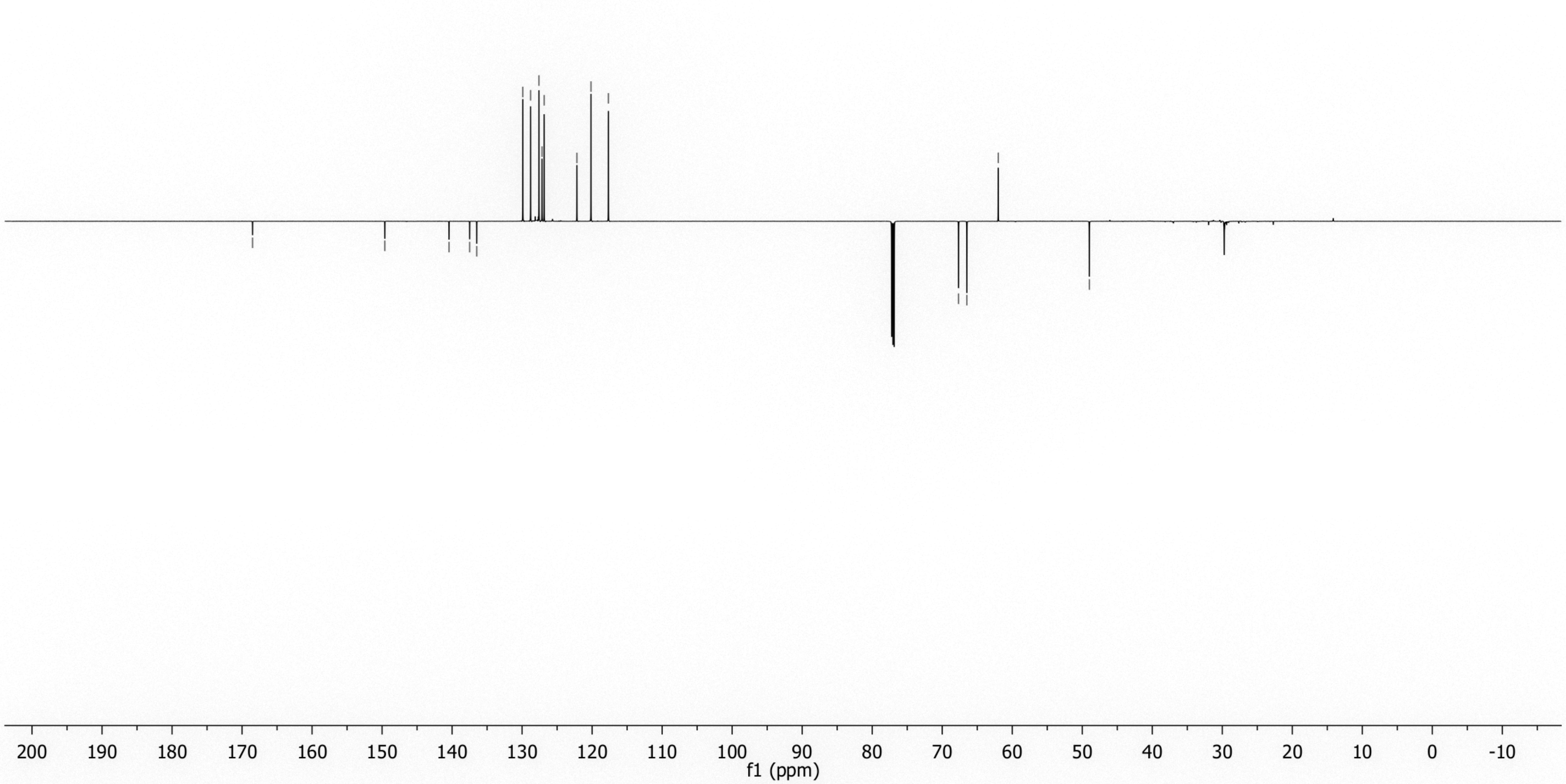

^{1}H NMR
400 MHz, $CDCl_3$

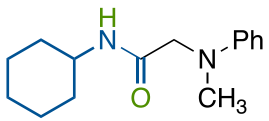

18

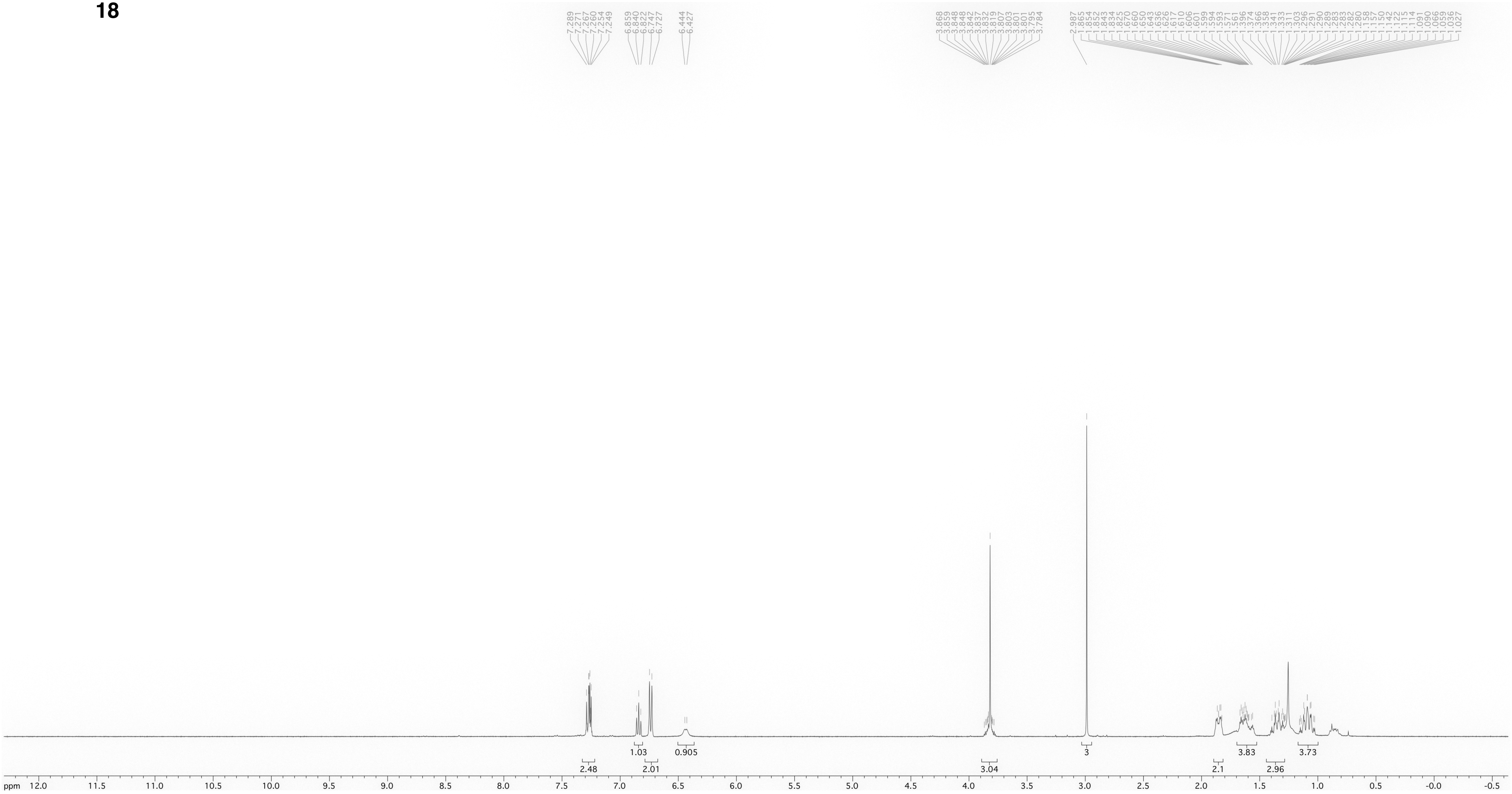

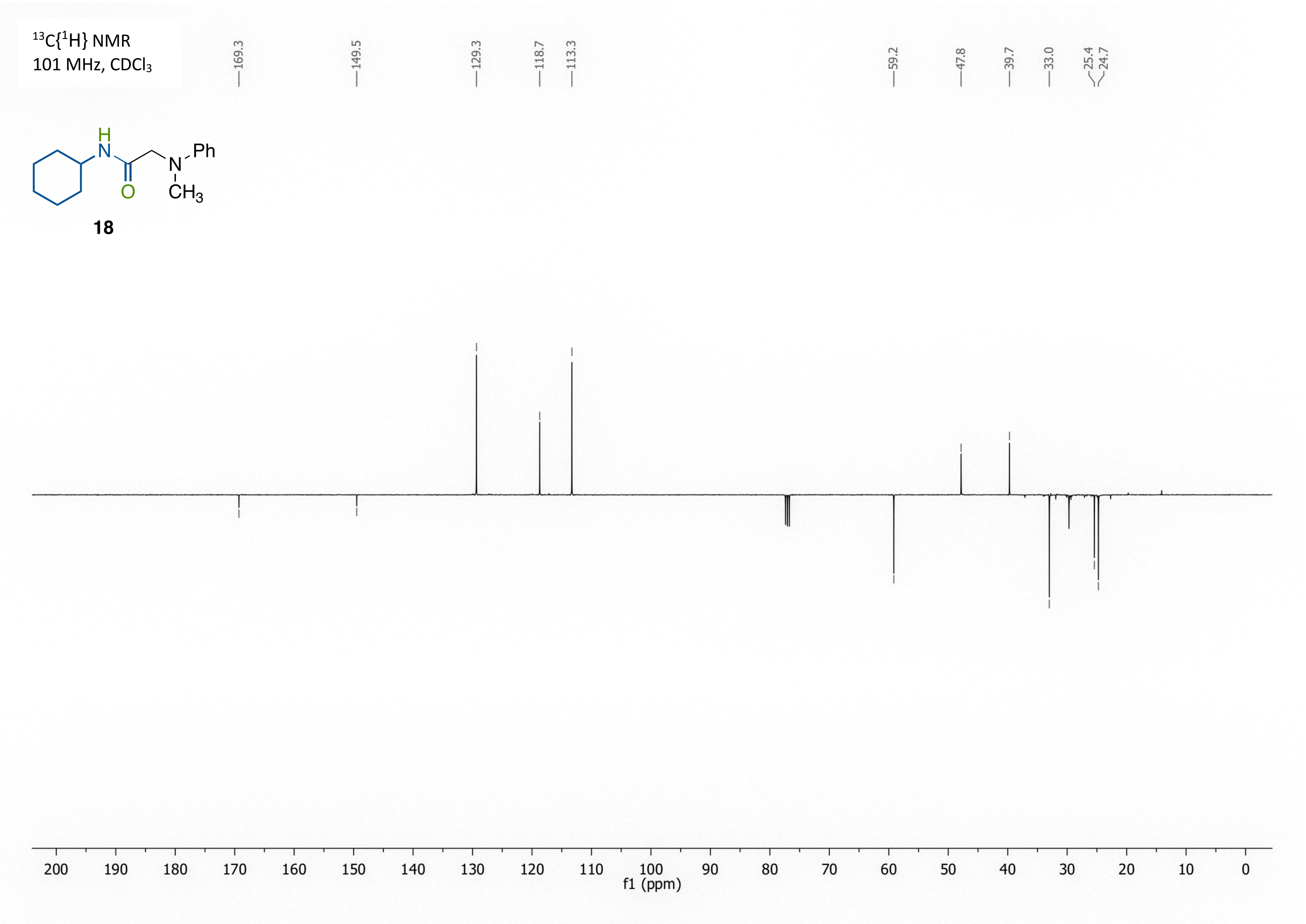

$^{13}C\{^{1}H\}$ NMR
101 MHz, $CDCl_3$
169.3
149.5
129.3
118.7
113.3
59.2
47.8
39.7
33.0
25.4
24.7
H
N
N
Ph
O
CH_3
18
200
190
180
170
160
150
140
130
120
110
100
90
80
70
60
50
40
30
20
10
0
f1 (ppm)

^1H NMR
(700 MHz, $CDCl_3$)

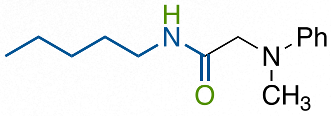

20

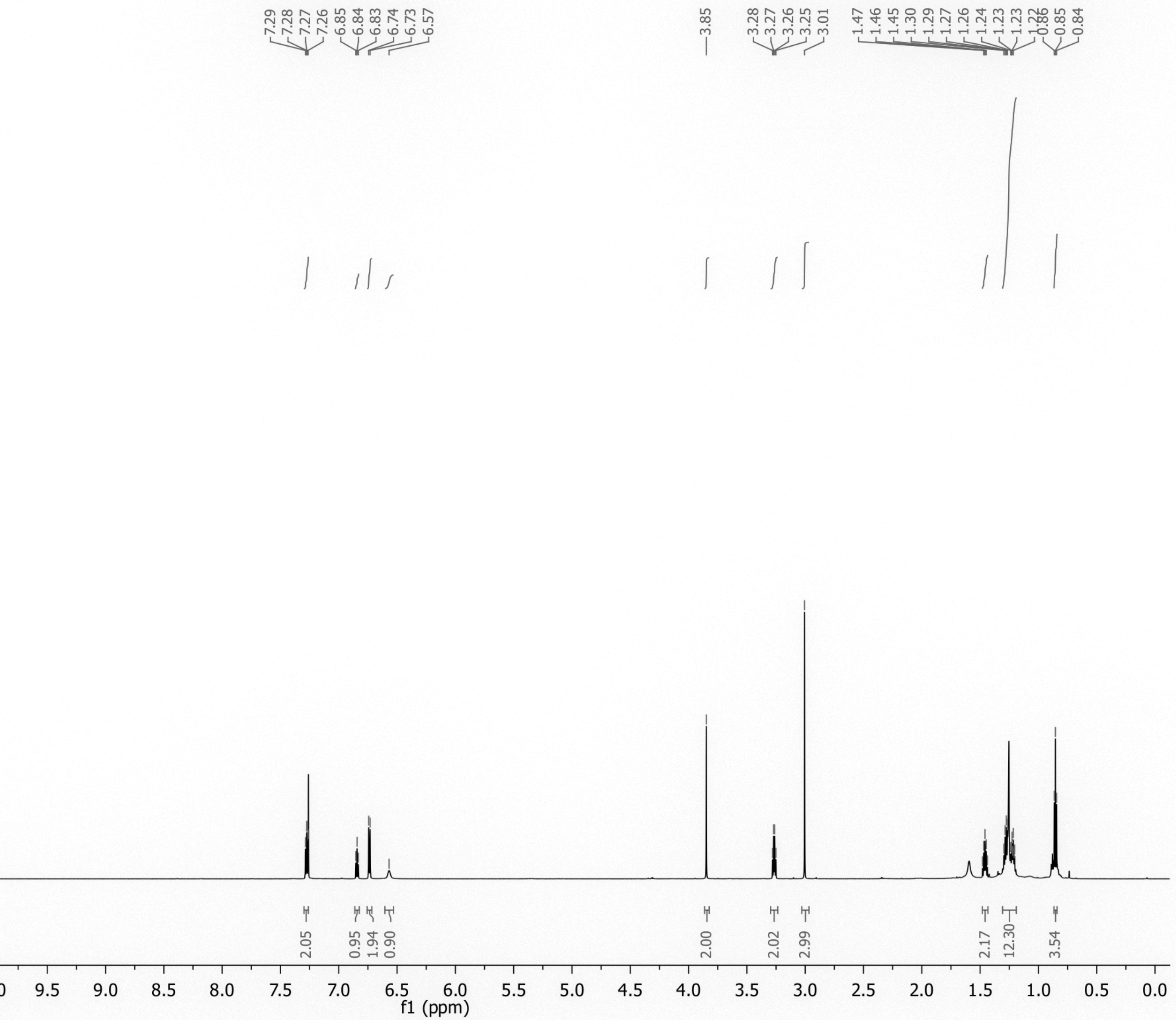

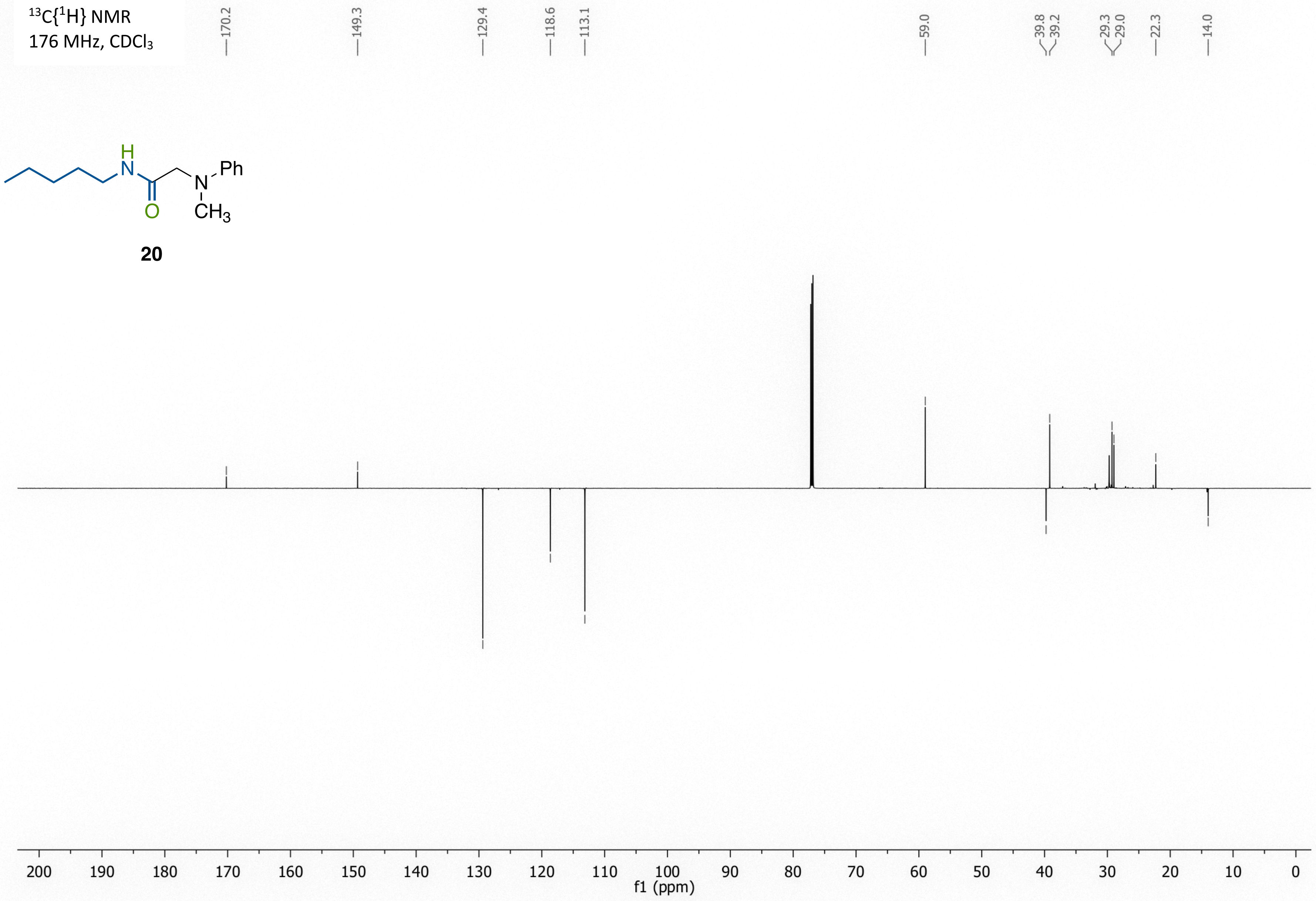

$^{13}C\{^1H\}$ NMR
176 MHz, $CDCl_3$
170.2
149.3
129.4
118.6
113.1
59.0
39.8
39.2
29.3
29.0
22.3
14.0
H
N
O
N
Ph
CH_3
200 190 180 170 160 150 140 130 120 110 100 90 80 70 60 50 40 30 20 10 0
f1 (ppm)

20

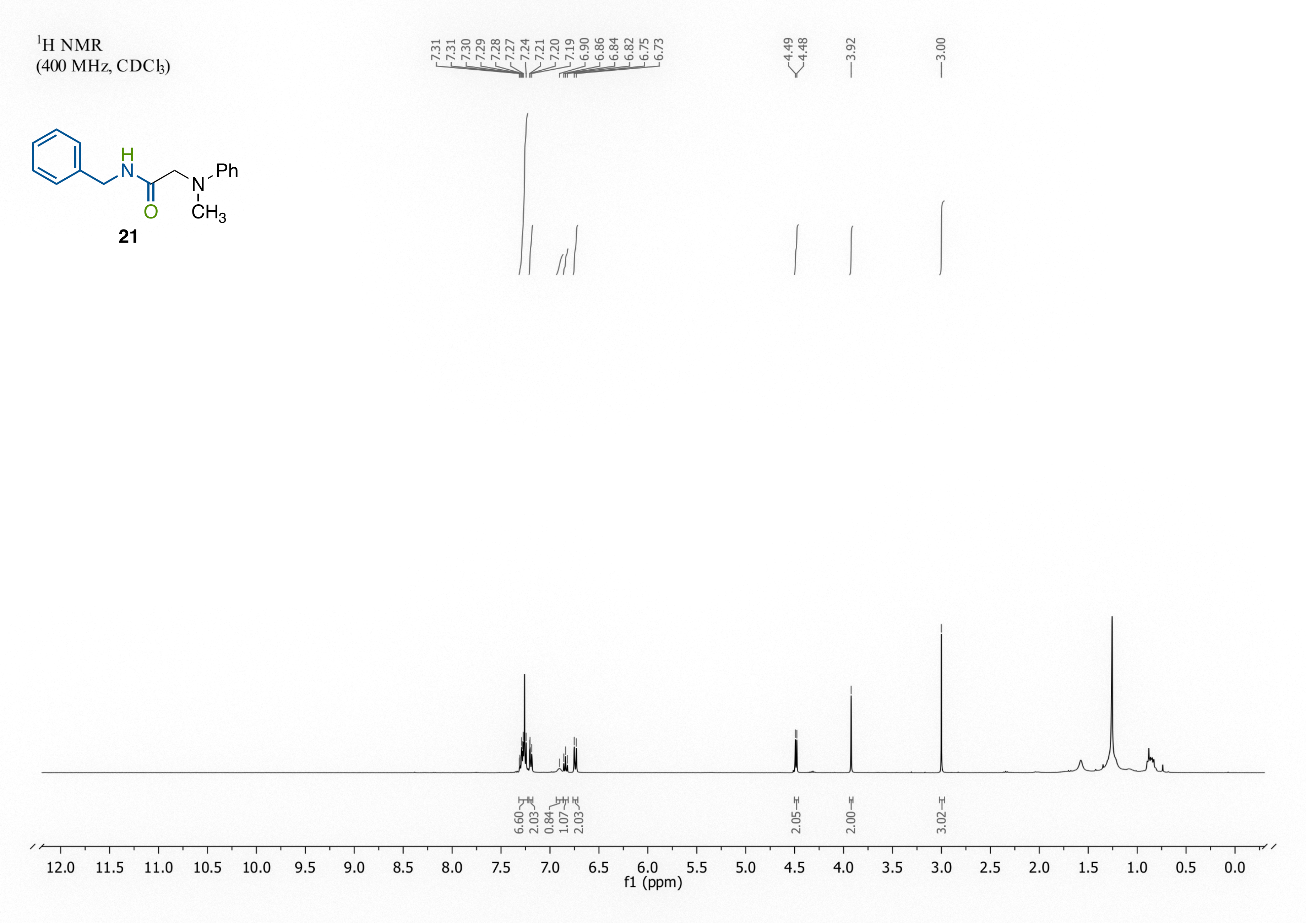

^{1}H NMR
(400 MHz, CDCl$_3$)
H
N
O
N
Ph
CH$_3$
21
7.31 7.31 7.30 7.29 7.28 7.27 7.24 7.21 7.20 7.19 6.90 6.86 6.84 6.82 6.75 6.73
4.49 4.48
3.92
3.00
6.60 2.03 0.84 1.07 2.03
2.05
2.00
3.02
12.0 11.5 11.0 10.5 10.0 9.5 9.0 8.5 8.0 7.5 7.0 6.5 6.0 5.5 5.0 4.5 4.0 3.5 3.0 2.5 2.0 1.5 1.0 0.5 0.0
f1 (ppm)

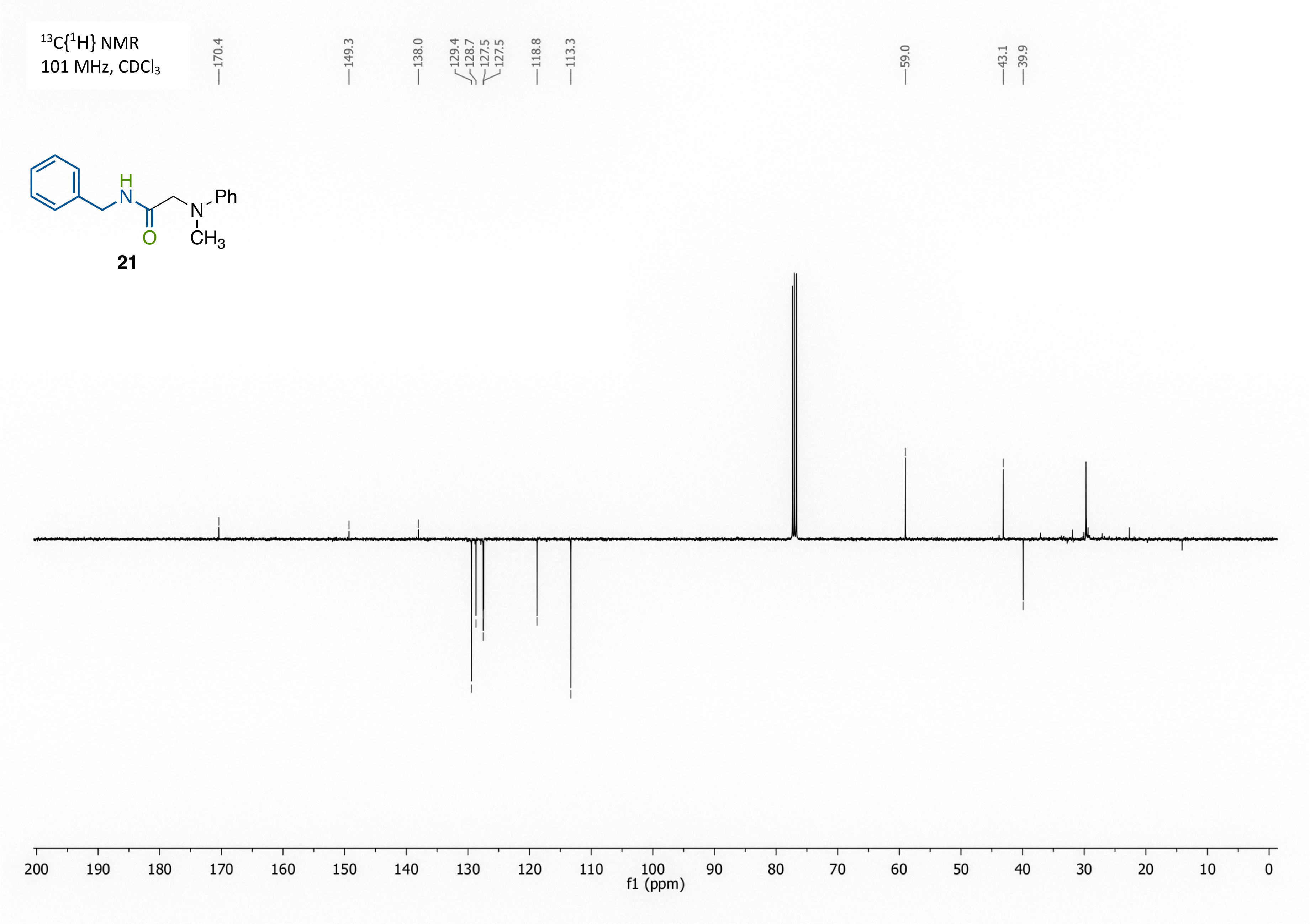

13C{1H} NMR
101 MHz, CDCl3
170.4
149.3
138.0
129.4
128.7
127.5
127.5
118.8
113.3
59.0
43.1
39.9
H
N
O
N
Ph
CH3
21
200 190 180 170 160 150 140 130 120 110 100 90 80 70 60 50 40 30 20 10 0
f1 (ppm)

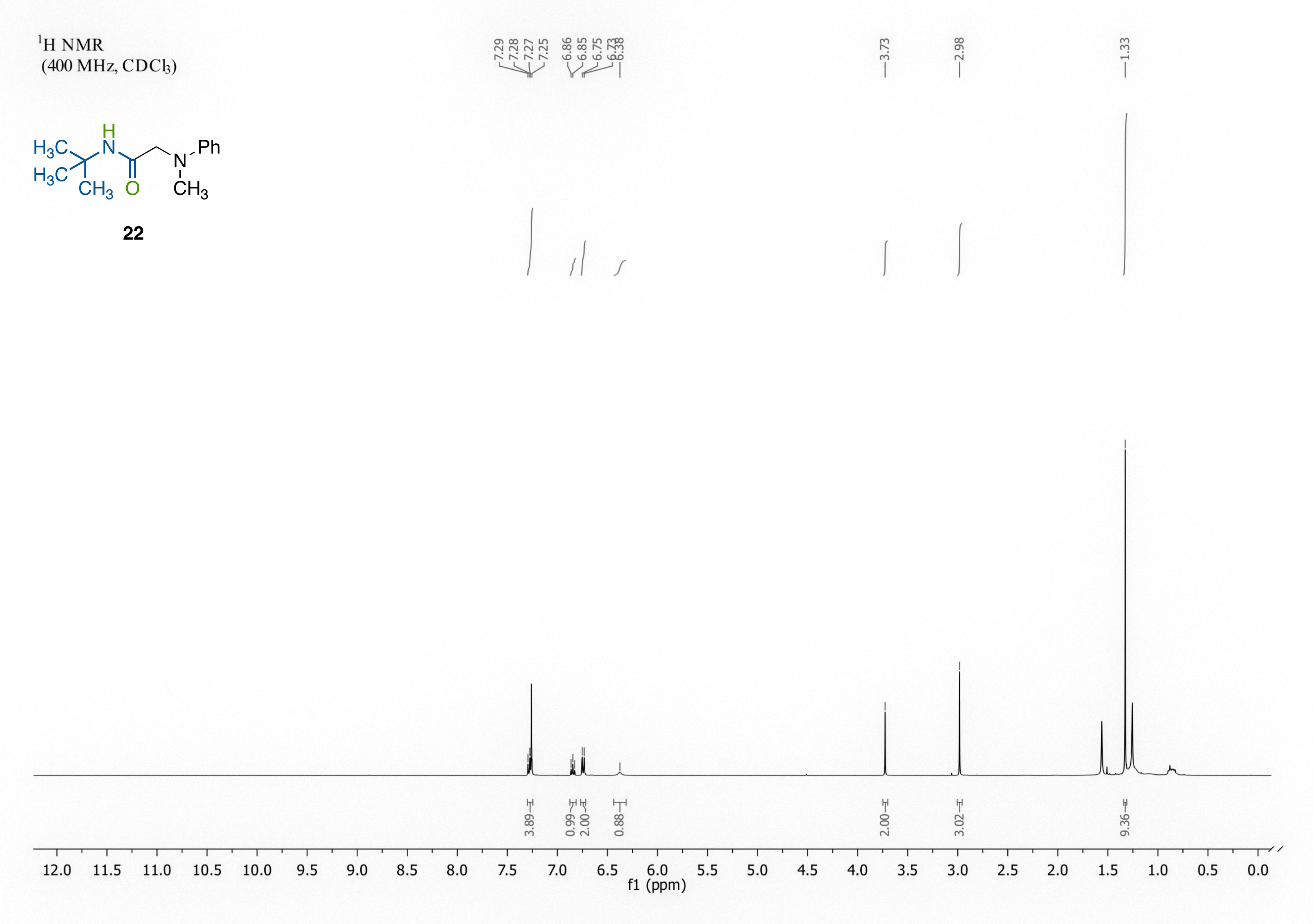

^{1}H NMR
(400 MHz, CDCl3)
H3C
H3C
CH3
H
N
O
N
Ph
CH3
22
7.29
7.28
7.27
7.25
6.86
6.85
6.75
6.73
6.38
3.73
2.98
1.33
3.89
0.99
2.00
0.88
2.00
3.02
9.36
12.0
11.5
11.0
10.5
10.0
9.5
9.0
8.5
8.0
7.5
7.0
6.5
6.0
5.5
5.0
4.5
4.0
3.5
3.0
2.5
2.0
1.5
1.0
0.5
0.0
f1 (ppm)

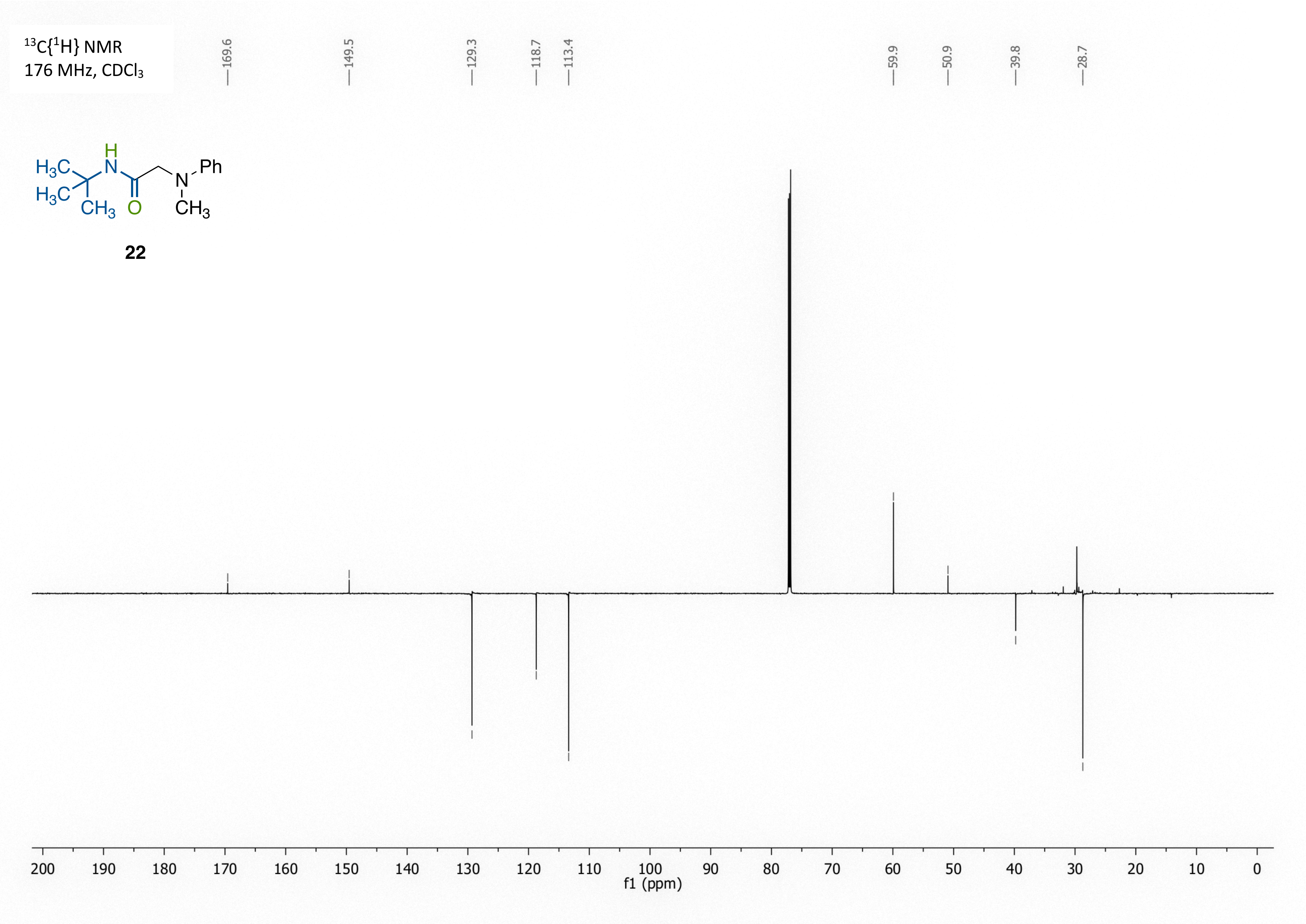

13C{1H} NMR
176 MHz, CDCl3
169.6
149.5
129.3
118.7
113.4
59.9
50.9
39.8
28.7
H3C
H
N
Ph
N
H3C
CH3
O
CH3
22
200
190
180
170
160
150
140
130
120
110
100
90
80
70
60
50
40
30
20
10
0
f1 (ppm)

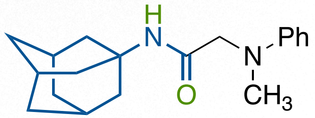

23

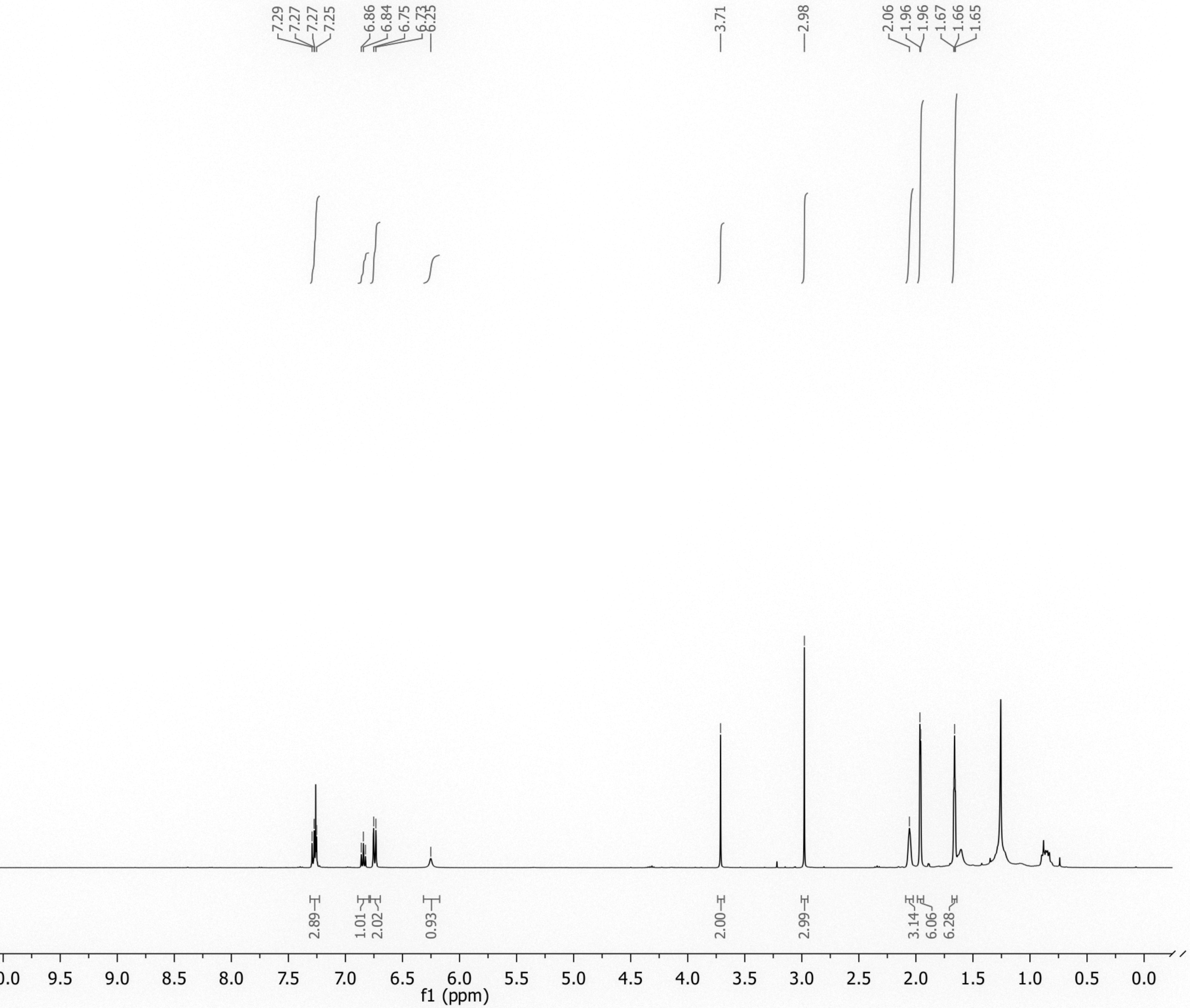

^{1}H NMR
(400 MHz, CDCl3)
7.29
7.27
7.27
7.25
6.86
6.84
6.75
6.73
6.25
3.71
2.98
2.06
1.96
1.96
1.67
1.66
1.65
2.89
1.01
2.02
0.93
2.00
2.99
3.14
6.06
6.28
12.0 11.5 11.0 10.5 10.0 9.5 9.0 8.5 8.0 7.5 7.0 6.5 6.0 5.5 5.0 4.5 4.0 3.5 3.0 2.5 2.0 1.5 1.0 0.5 0.0
f1 (ppm)

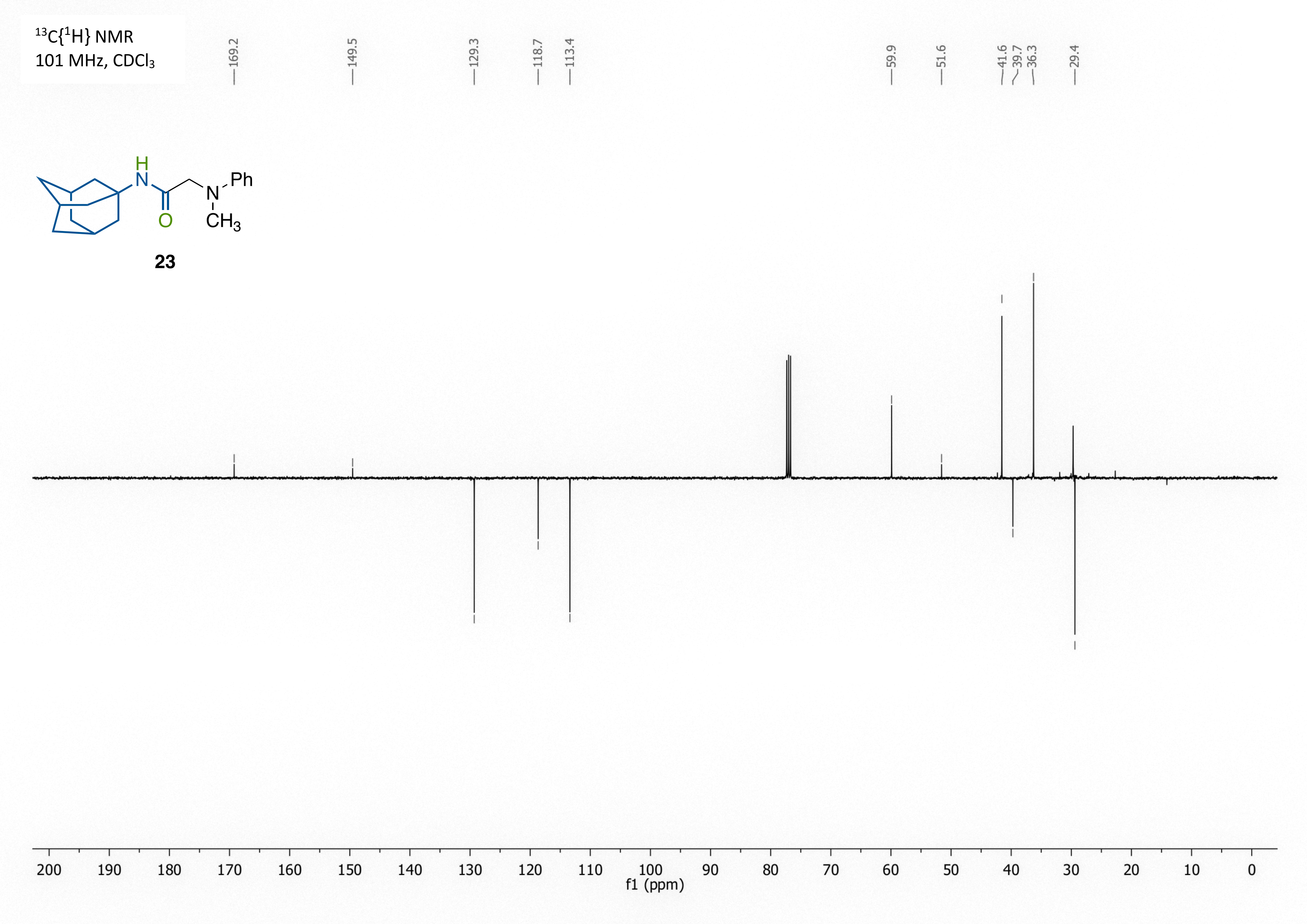
13C{1H} NMR
101 MHz, CDCl3
169.2
149.5
129.3
118.7
113.4
59.9
51.6
41.6
39.7
36.3
29.4
H
N
O
N
Ph
CH3
23
200 190 180 170 160 150 140 130 120 110 100 90 80 70 60 50 40 30 20 10 0
f1 (ppm)

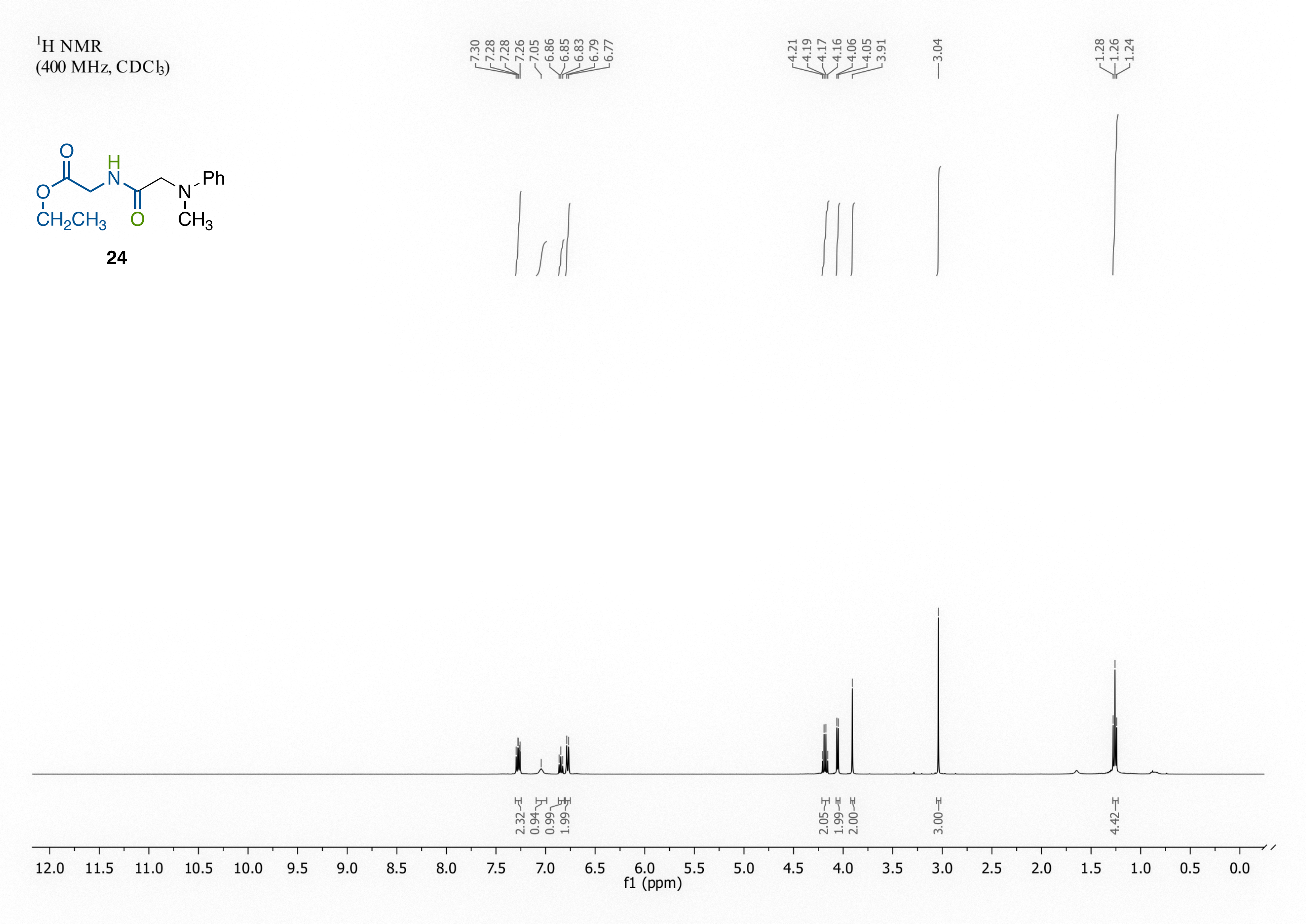

^{1}H NMR
(400 MHz, $CDCl_3$)
24
7.30
7.28
7.28
7.26
7.05
6.86
6.85
6.83
6.79
6.77
4.21
4.19
4.17
4.16
4.06
4.05
3.91
3.04
1.28
1.26
1.24
2.32
0.94
0.99
1.99
2.05
1.99
2.00
3.00
4.42
f1 (ppm)

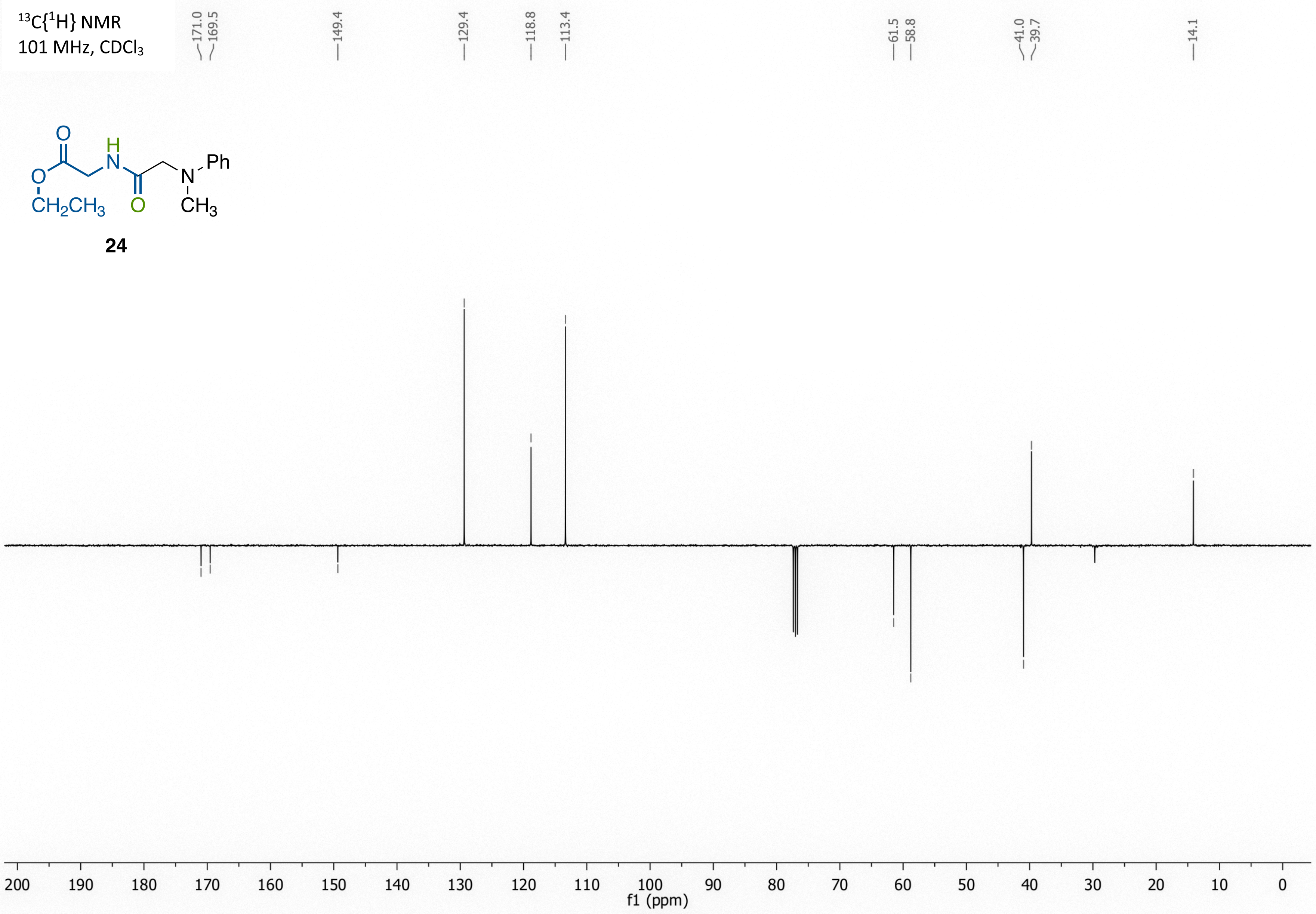

$^{13}C\{^1H\}$ NMR
101 MHz, $CDCl_3$
171.0
169.5
149.4
129.4
118.8
113.4
61.5
58.8
41.0
39.7
14.1
O
H
N
Ph
O
N
CH_2CH_3
O
CH_3
200 190 180 170 160 150 140 130 120 110 100 90 80 70 60 50 40 30 20 10 0
f1 (ppm)

24

^{1}H NMR
(400 MHz, $CDCl_3$)

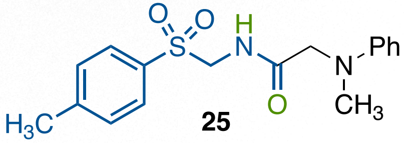

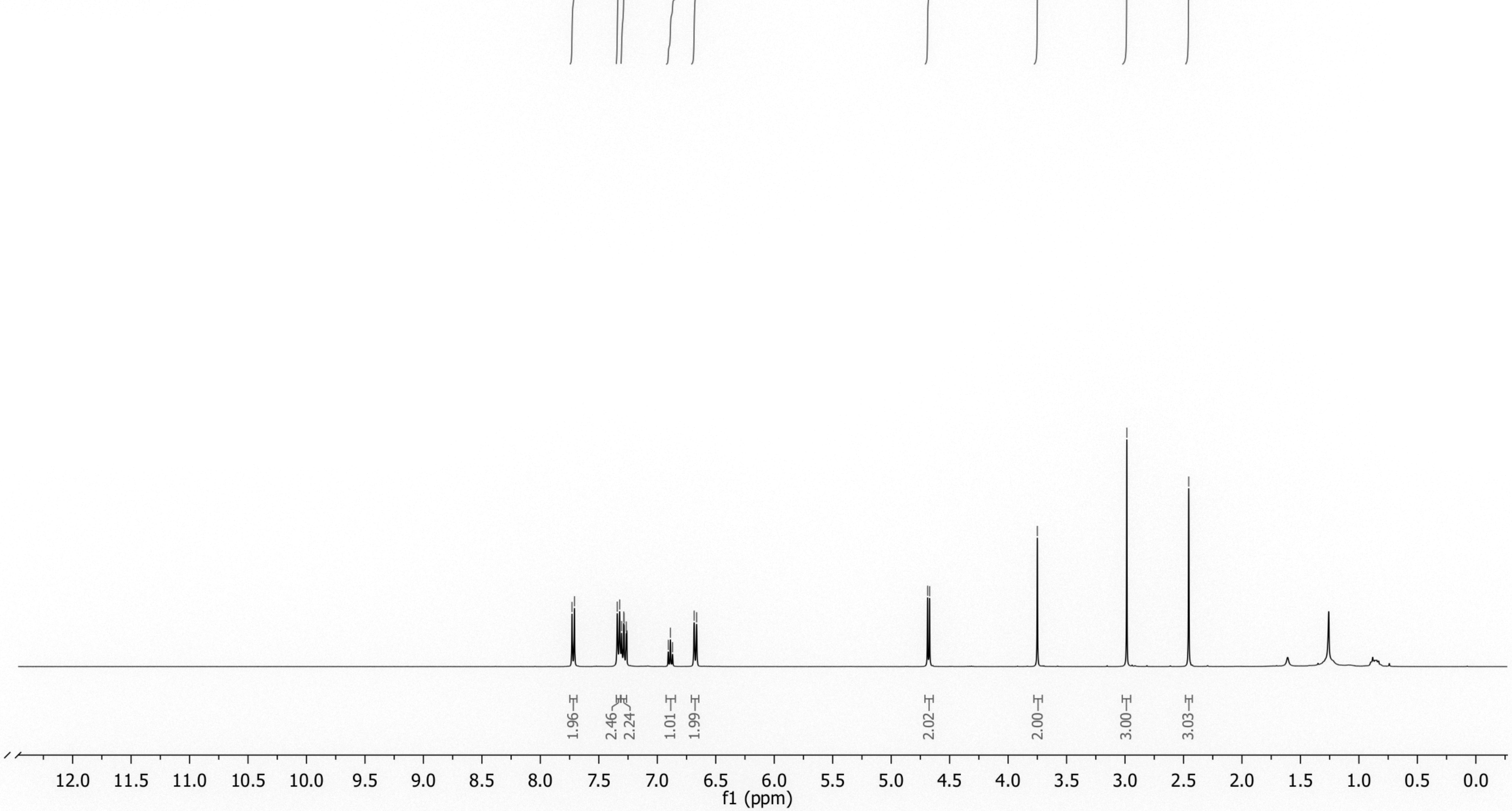

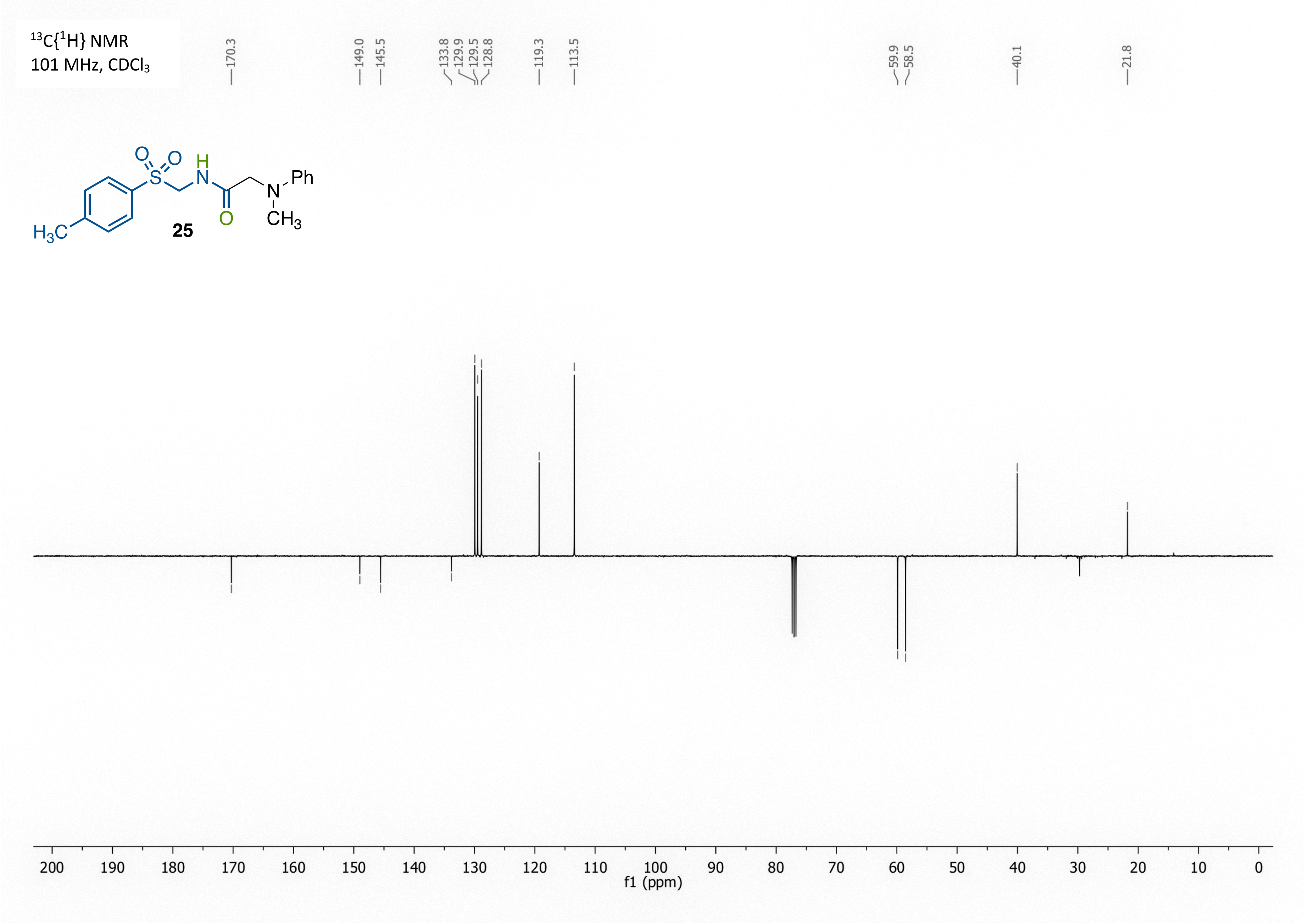
$^{13}C\{^1H\}$ NMR
101 MHz, $CDCl_3$
170.3
149.0
145.5
133.8
129.9
129.5
128.8
119.3
113.5
59.9
58.5
40.1
21.8
25
Ph
CH_3
H_3C
200 190 180 170 160 150 140 130 120 110 100 90 80 70 60 50 40 30 20 10 0
f1 (ppm)

^{1}H NMR
700 MHz, $CDCl_3$

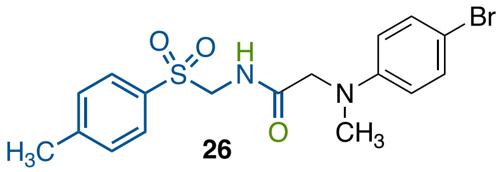

26

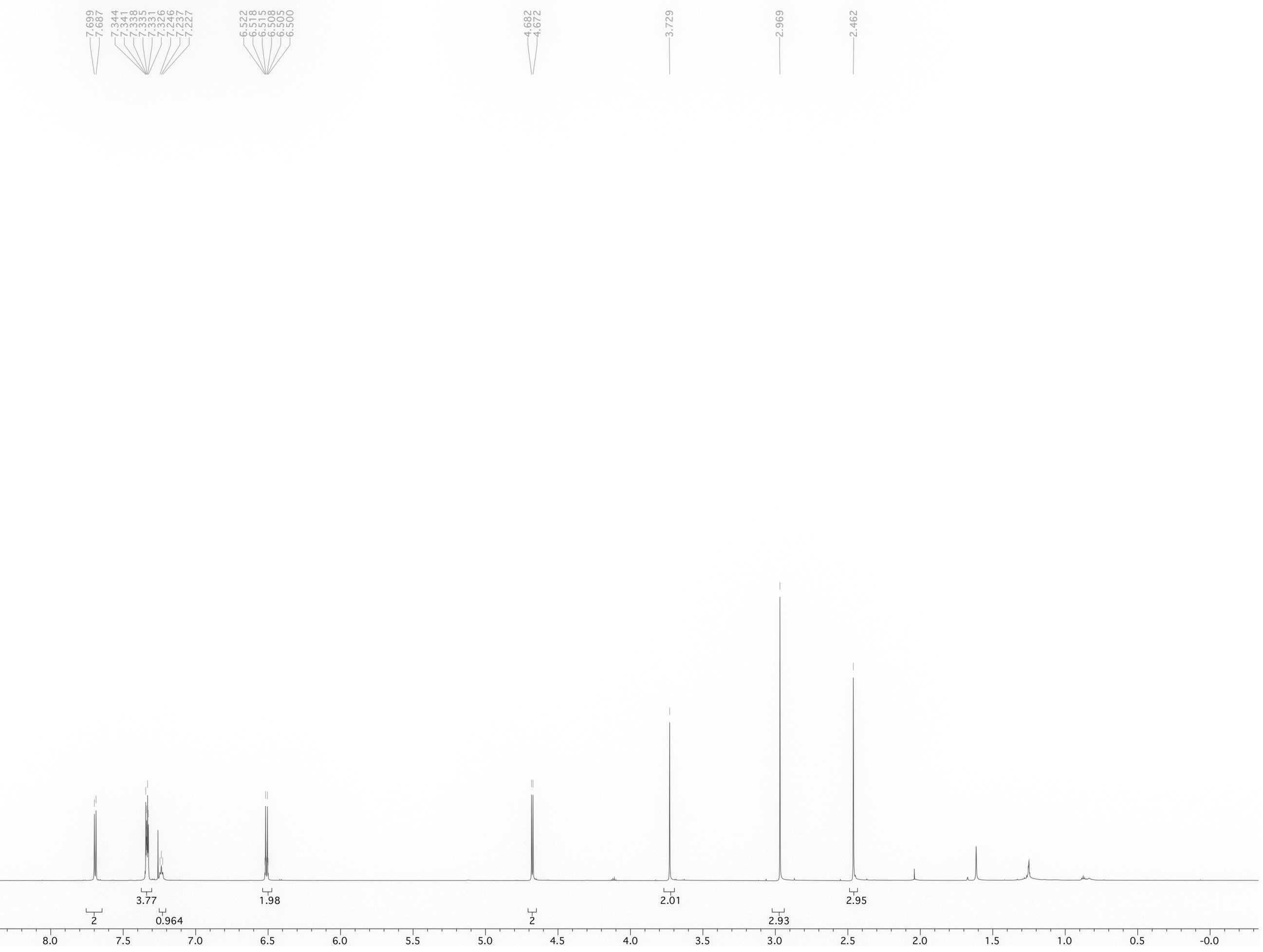

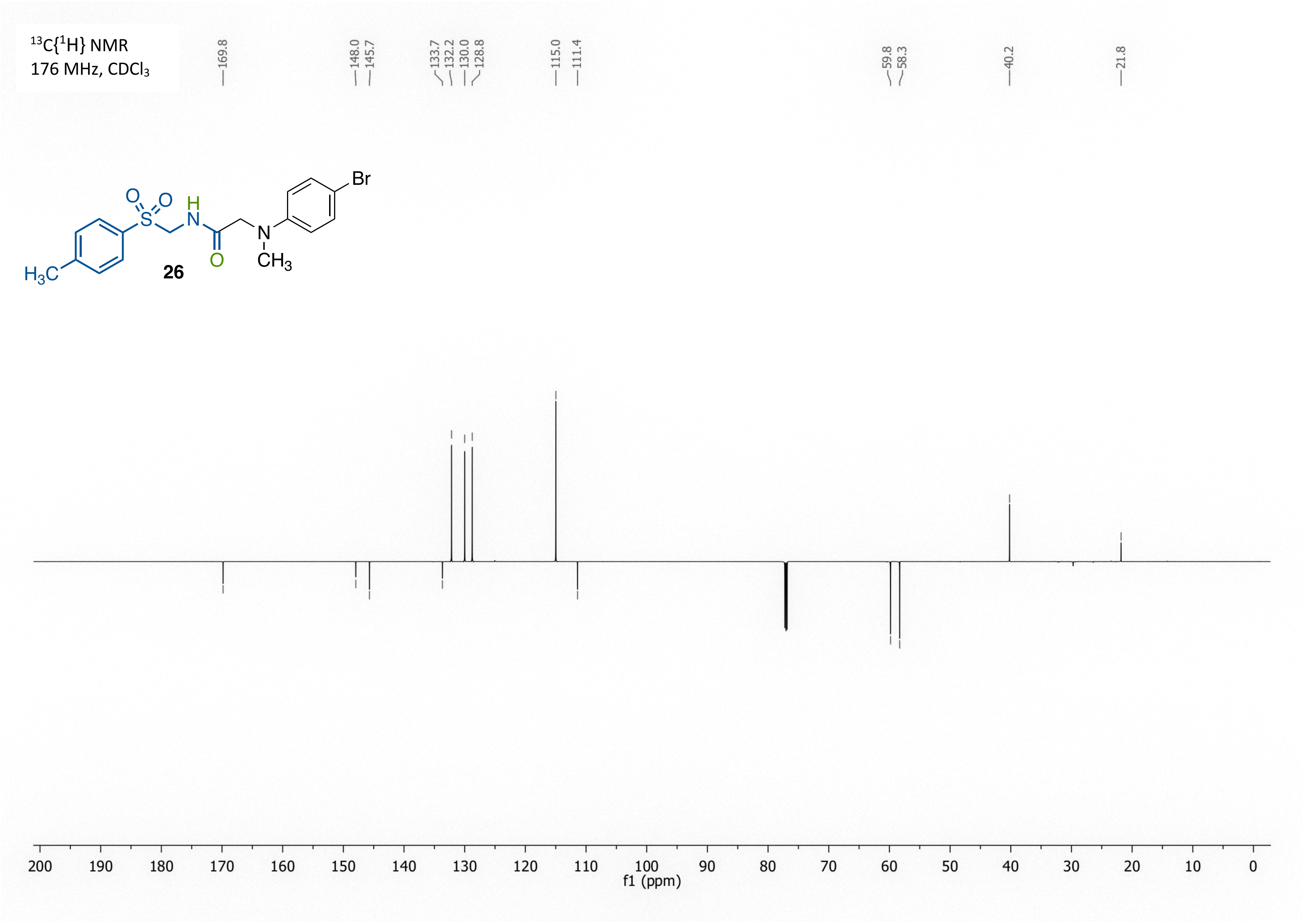
$^{13}C\{^1H\}$ NMR
176 MHz, $CDCl_3$
169.8
148.0
145.7
133.7
132.2
130.0
128.8
115.0
111.4
59.8
58.3
40.2
21.8
Br
O
O
S
H
N
N
O
CH_3
H_3C
26
200 190 180 170 160 150 140 130 120 110 100 90 80 70 60 50 40 30 20 10 0
f1 (ppm)

^{1}H NMR
700 MHz, $CDCl_3$

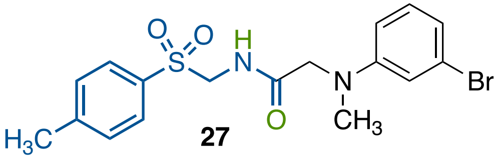

27

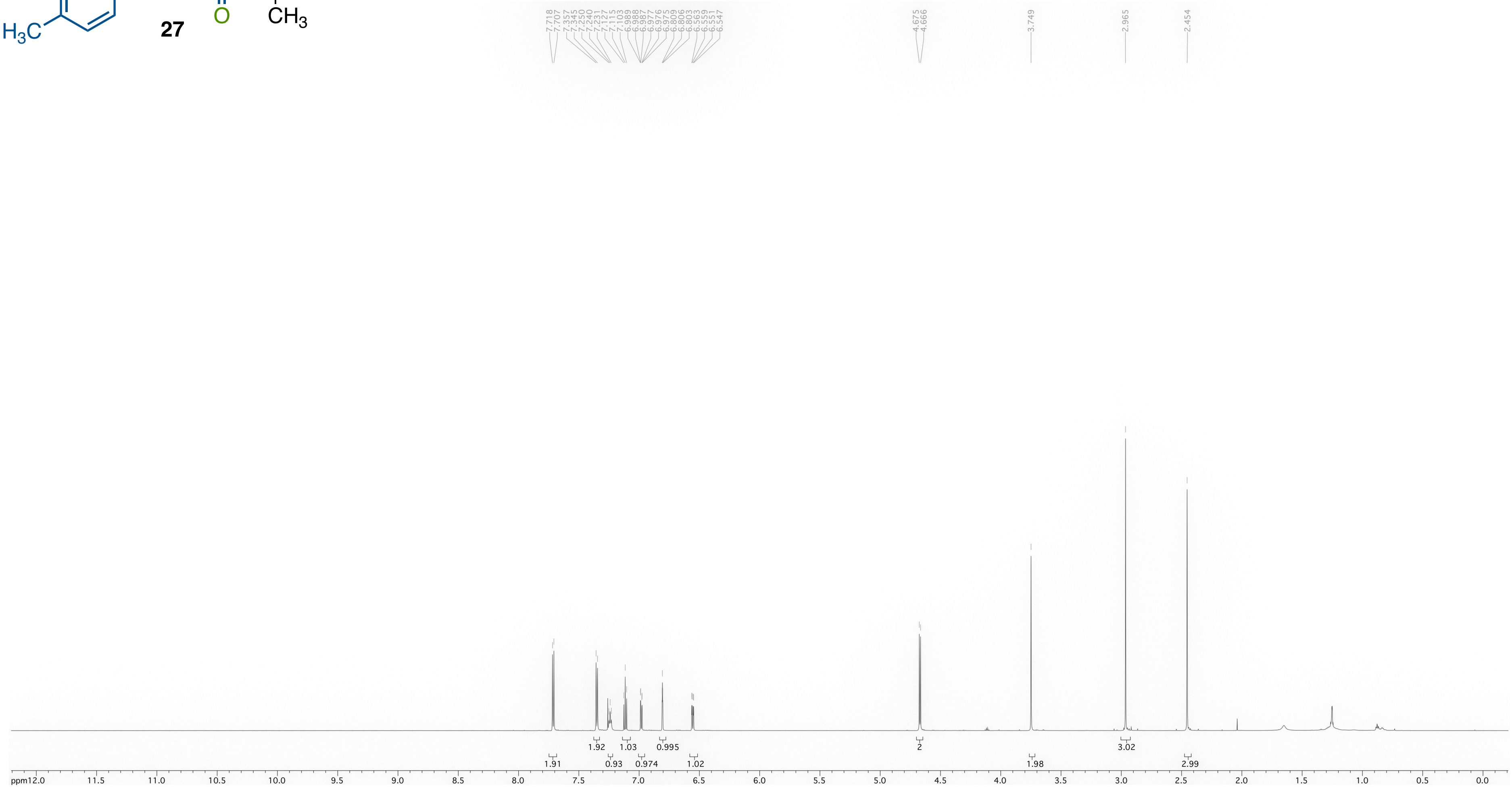

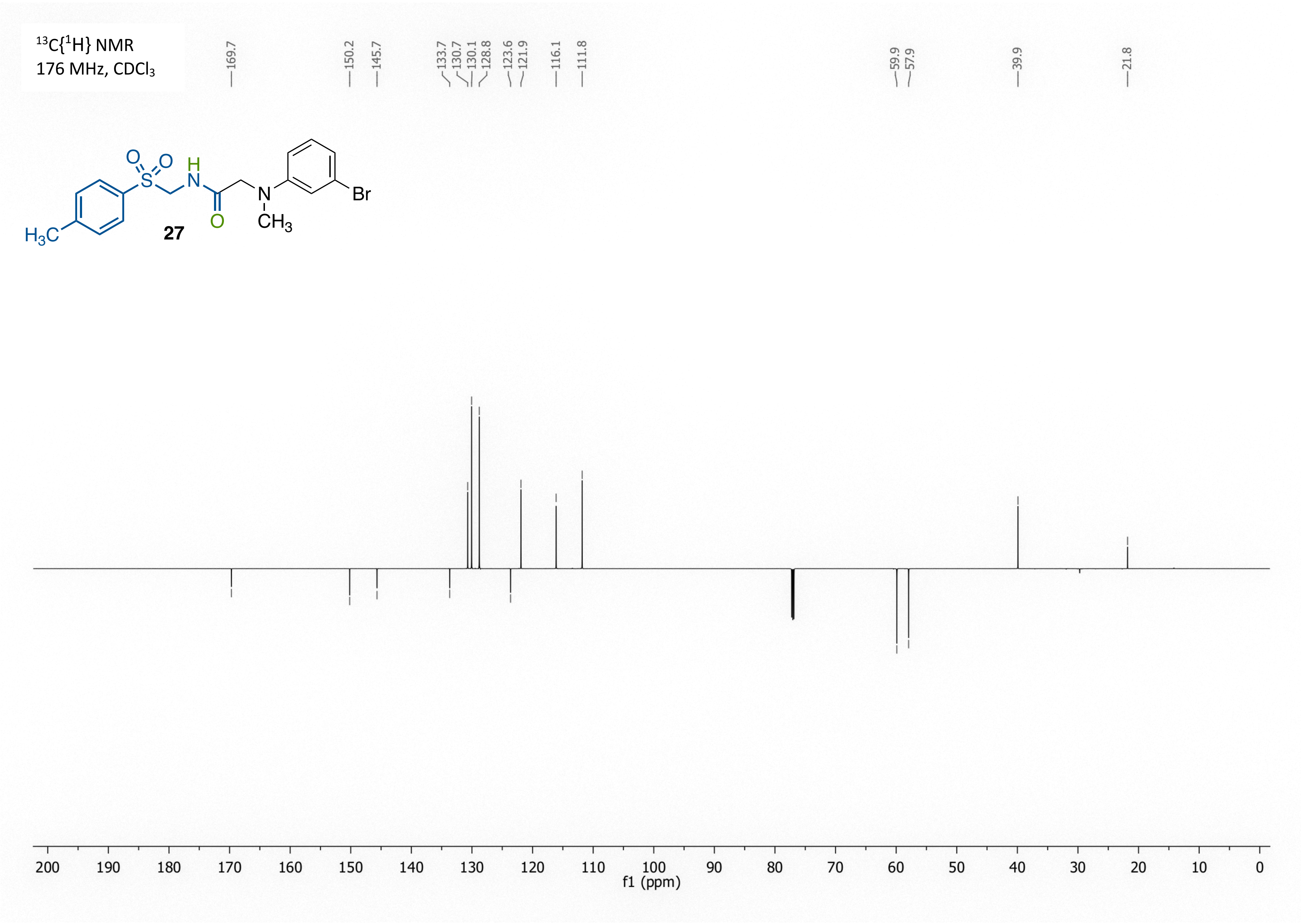

$^{13}C\{^1H\}$ NMR
176 MHz, $CDCl_3$
169.7
150.2
145.7
133.7
130.7
130.1
128.8
123.6
121.9
116.1
111.8
59.9
57.9
39.9
21.8
27
Br
CH_3
H_3C
f1 (ppm)

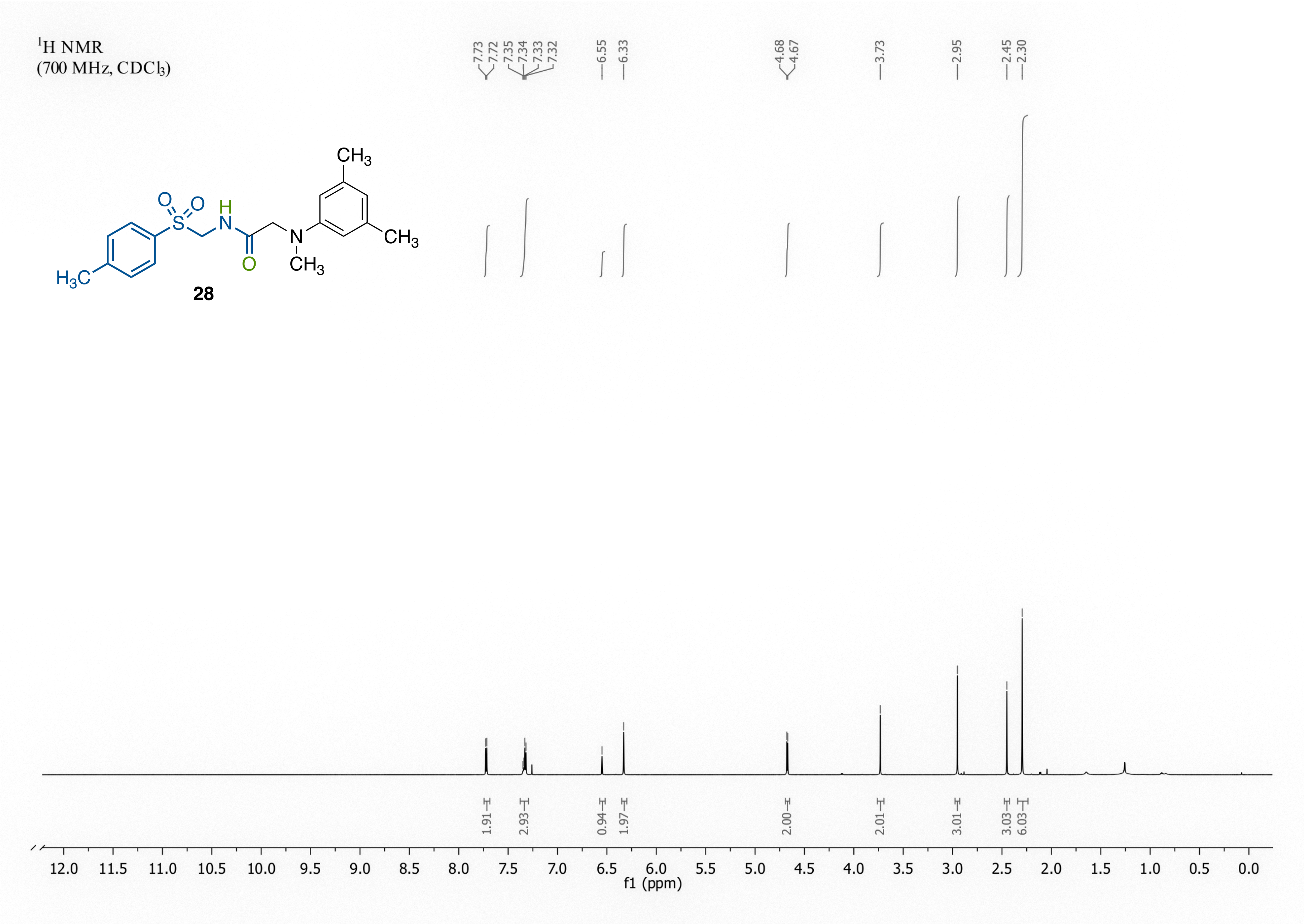

1H NMR
(700 MHz, CDCl3)
7.73
7.72
7.35
7.34
7.33
7.32
6.55
6.33
4.68
4.67
3.73
2.95
2.45
2.30
CH3
O
O
H
N
S
N
CH3
H3C
O
CH3
28
1.91
2.93
0.94
1.97
2.00
2.01
3.01
3.03
6.03
12.0
11.5
11.0
10.5
10.0
9.5
9.0
8.5
8.0
7.5
7.0
6.5
6.0
5.5
5.0
4.5
4.0
3.5
3.0
2.5
2.0
1.5
1.0
0.5
0.0
f1 (ppm)

$^{13}C\{^{1}H\}$ NMR
176 MHz, $CDCl_3$

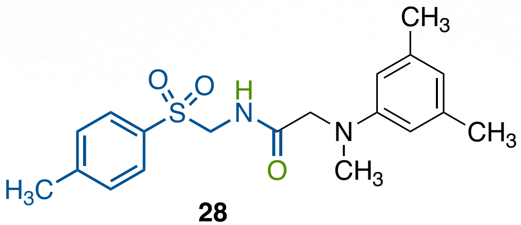

28

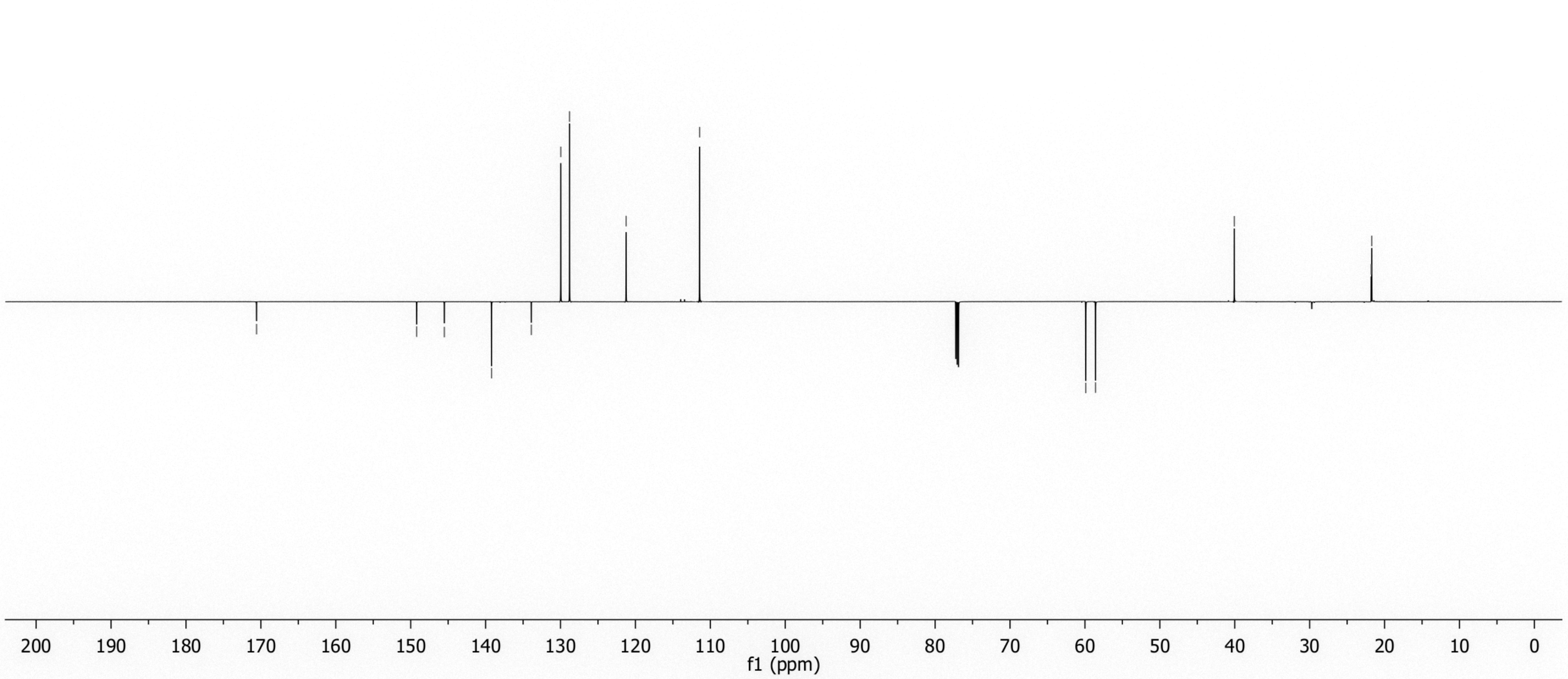

^{1}H NMR
(700 MHz, $CDCl_3$)

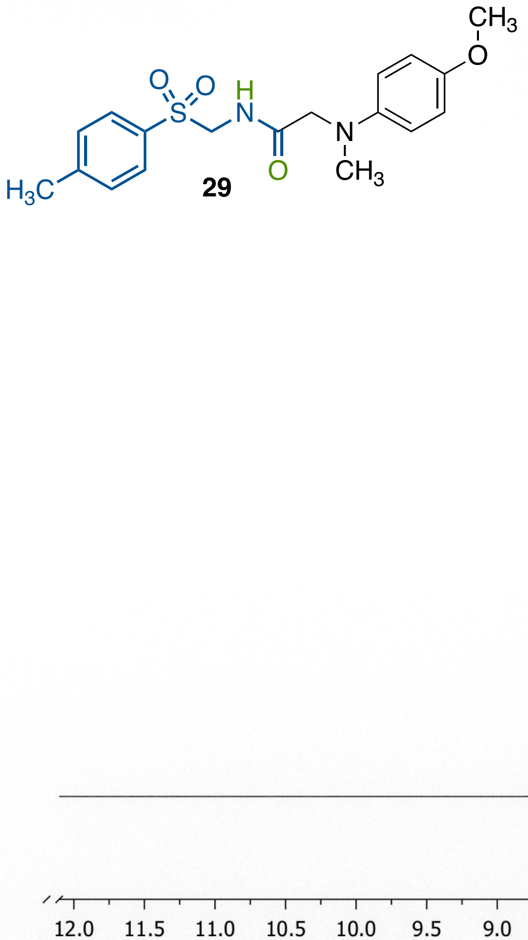

29

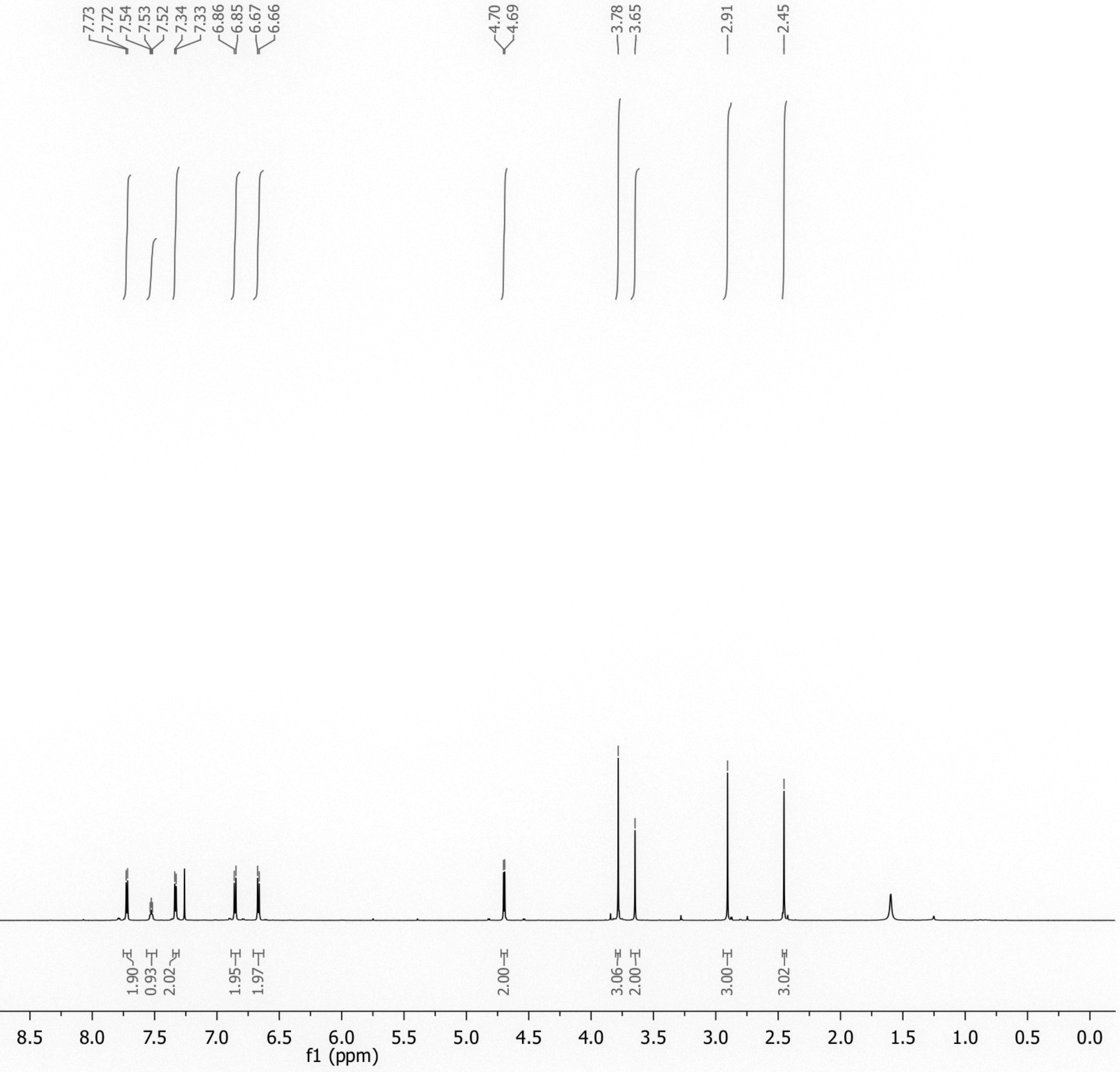

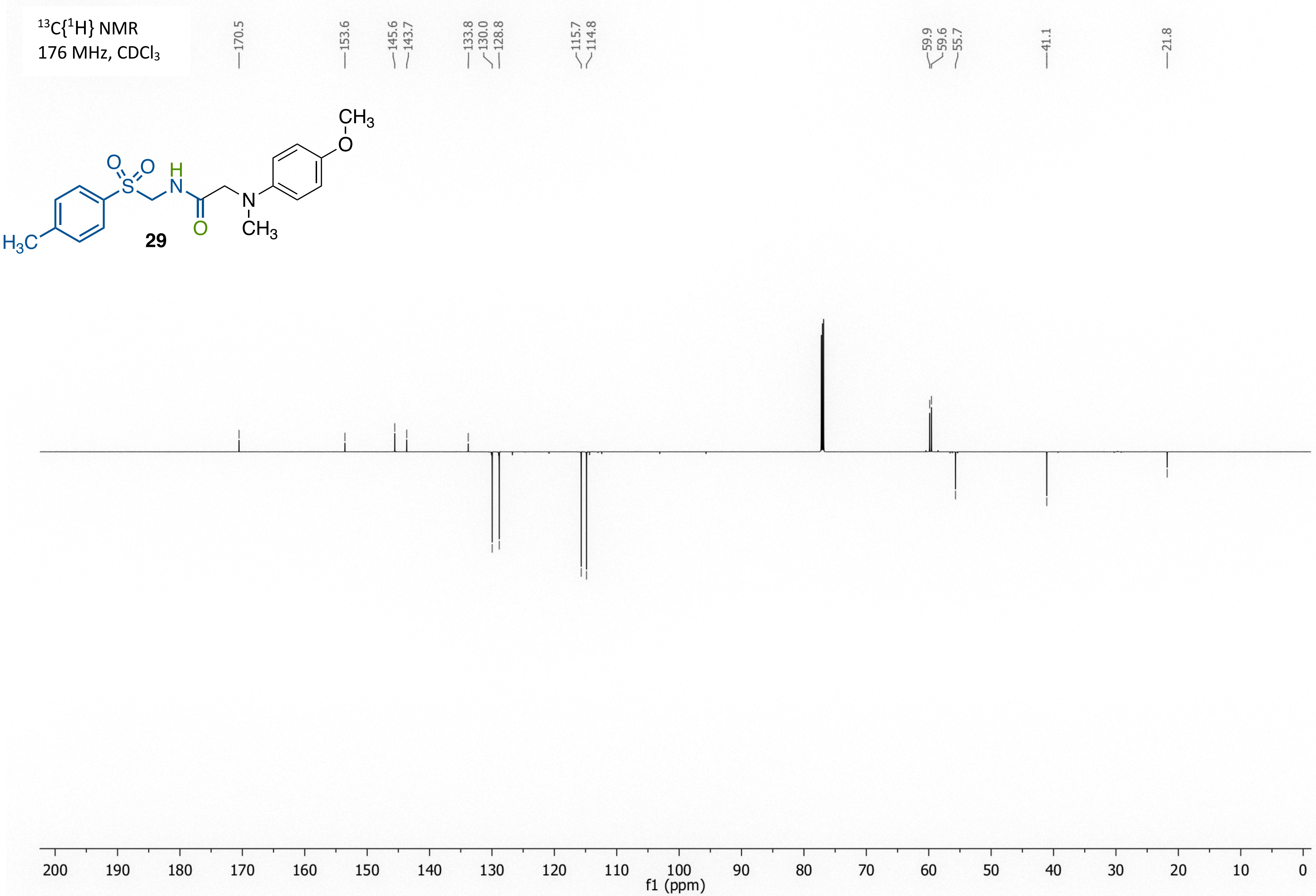

13C{1H} NMR
176 MHz, CDCl3
170.5
153.6
145.6
143.7
133.8
130.0
128.8
115.7
114.8
59.9
59.6
55.7
41.1
21.8
29
200 190 180 170 160 150 140 130 120 110 100 90 80 70 60 50 40 30 20 10 0
f1 (ppm)

^{1}H NMR
700 MHz, $CDCl_3$

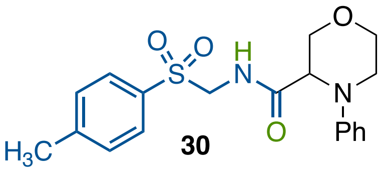

30

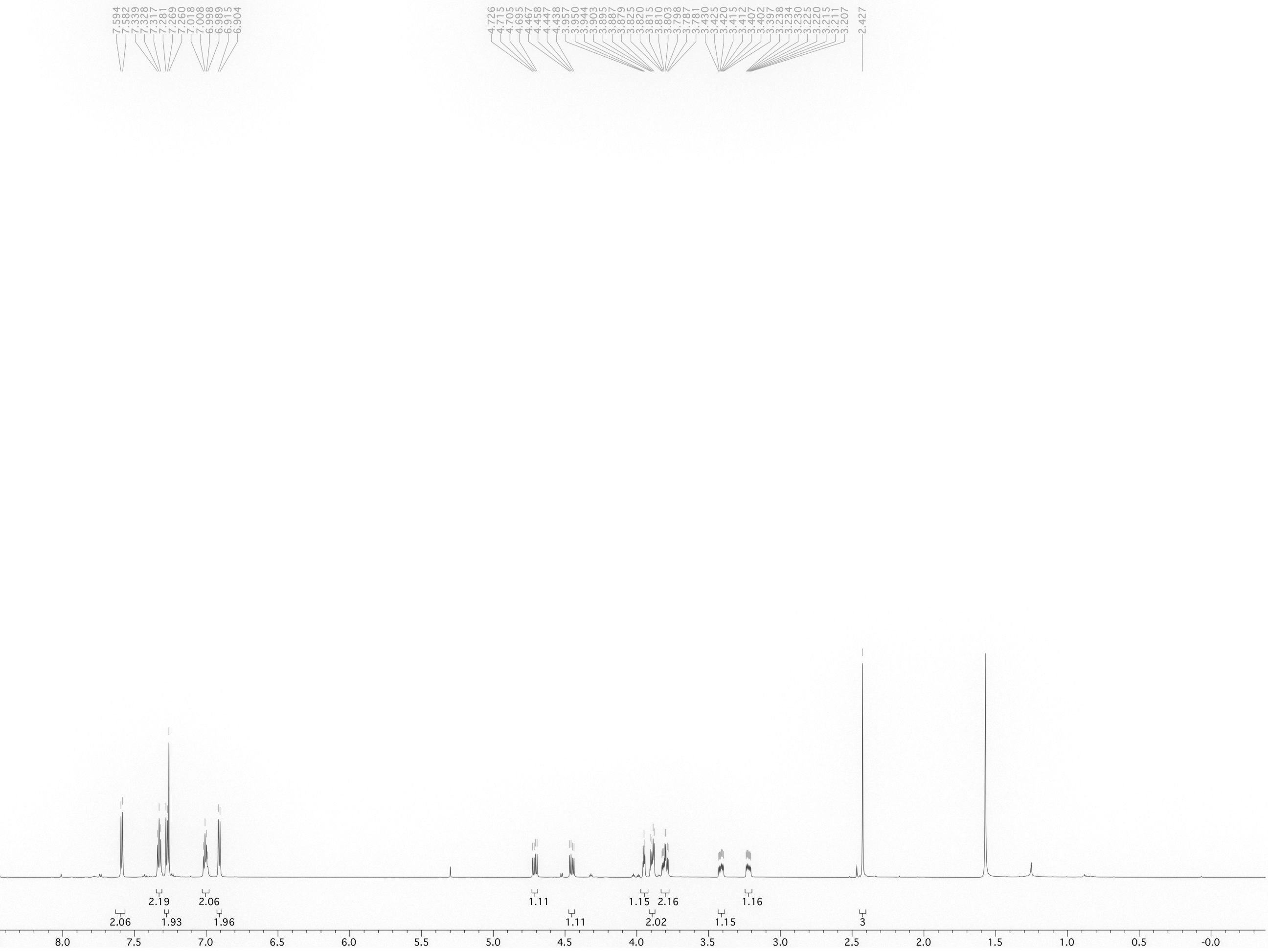

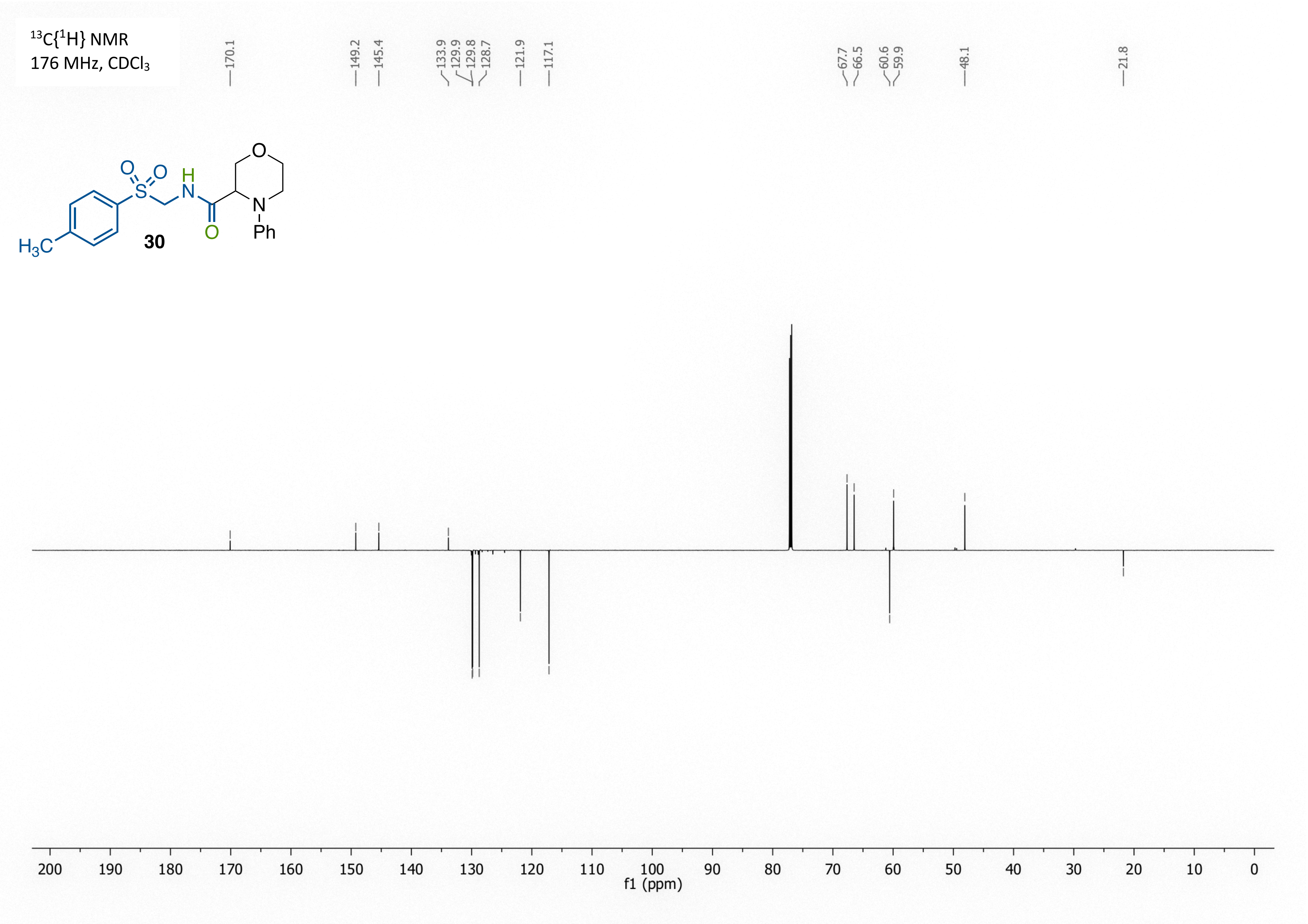

$^{13}C\{^1H\}$ NMR
176 MHz, $CDCl_3$
170.1
149.2
145.4
133.9
129.9
129.8
128.7
121.9
117.1
67.7
66.5
60.6
59.9
48.1
21.8
H3C
O
S
O
H
N
O
N
Ph
30
200
190
180
170
160
150
140
130
120
110
100
90
80
70
60
50
40
30
20
10
0
f1 (ppm)

^{1}H NMR
700 MHz, $CDCl_3$

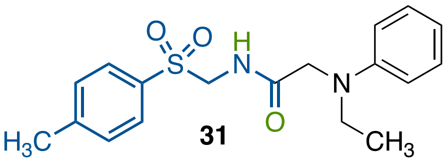

31

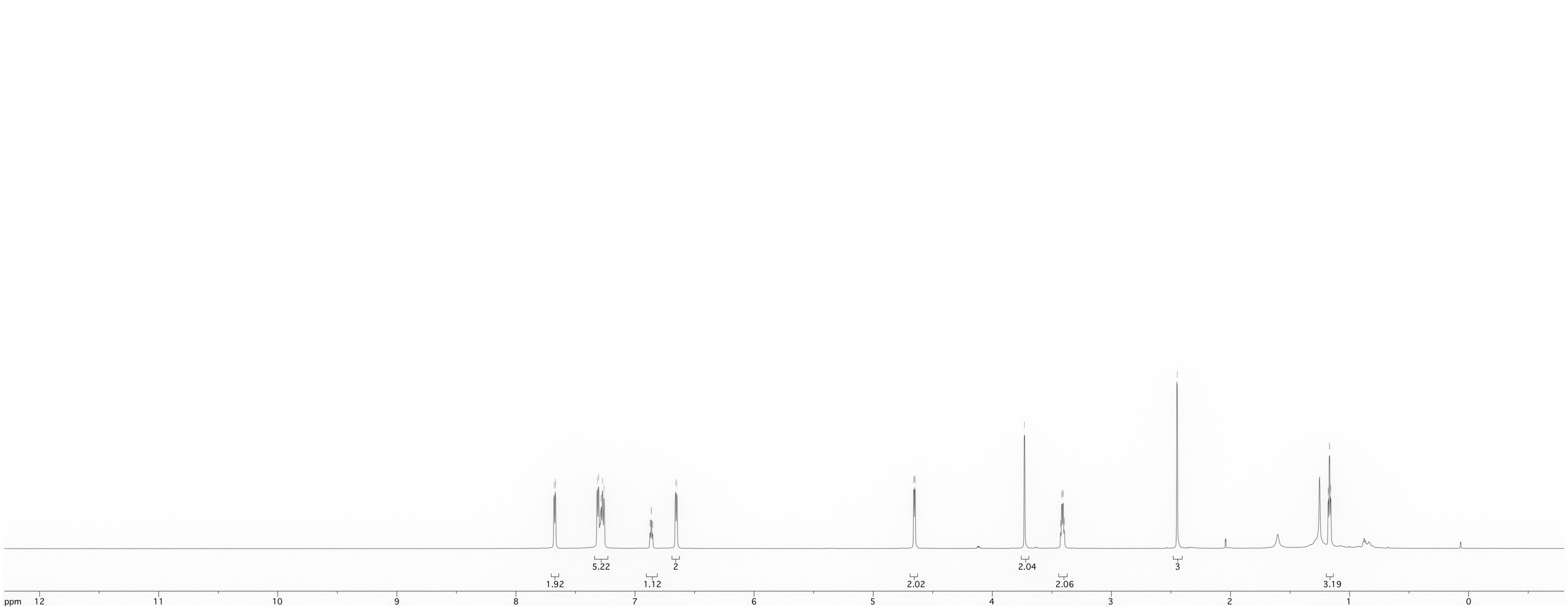

$^{13}C\{^{1}H\}$ NMR
176 MHz, $CDCl_3$

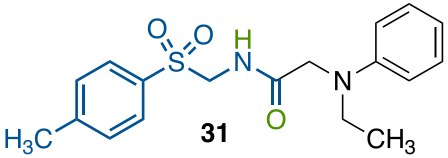

31

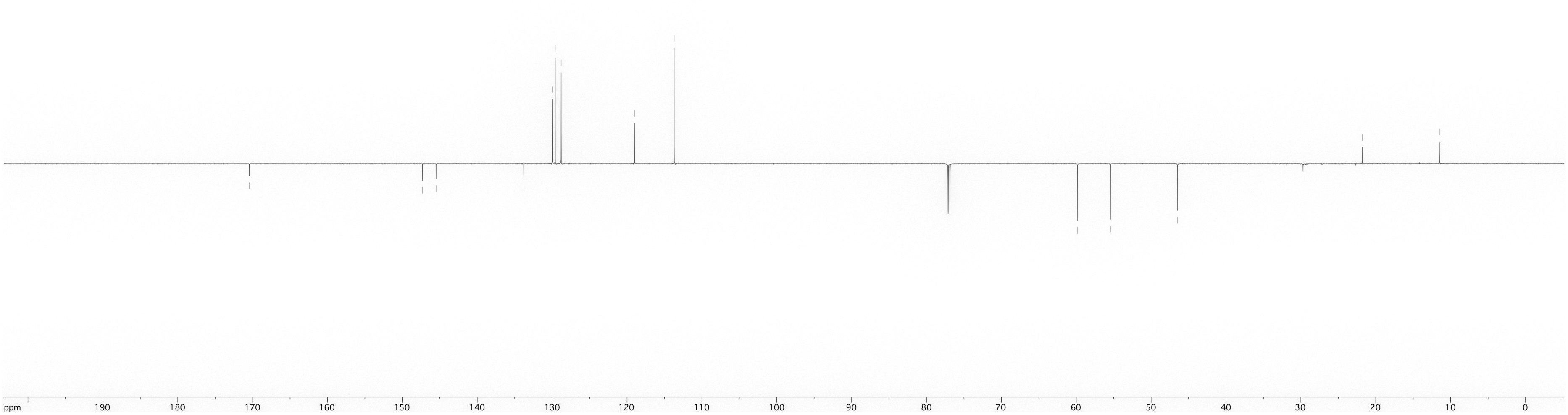

^{1}H NMR
400 MHz, $CDCl_3$

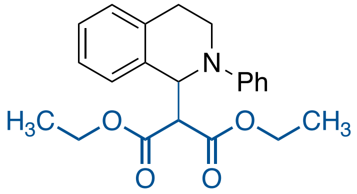

32

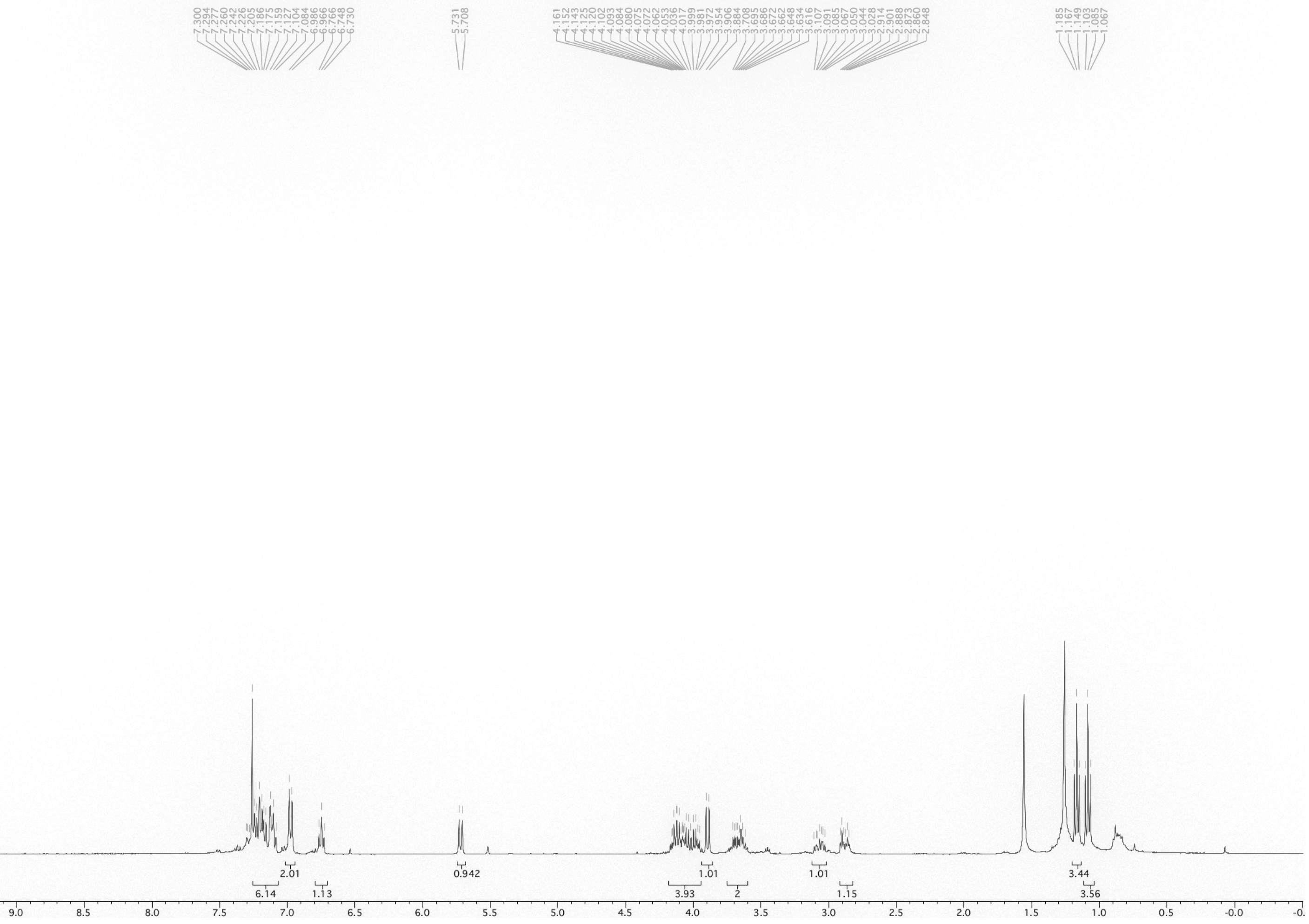

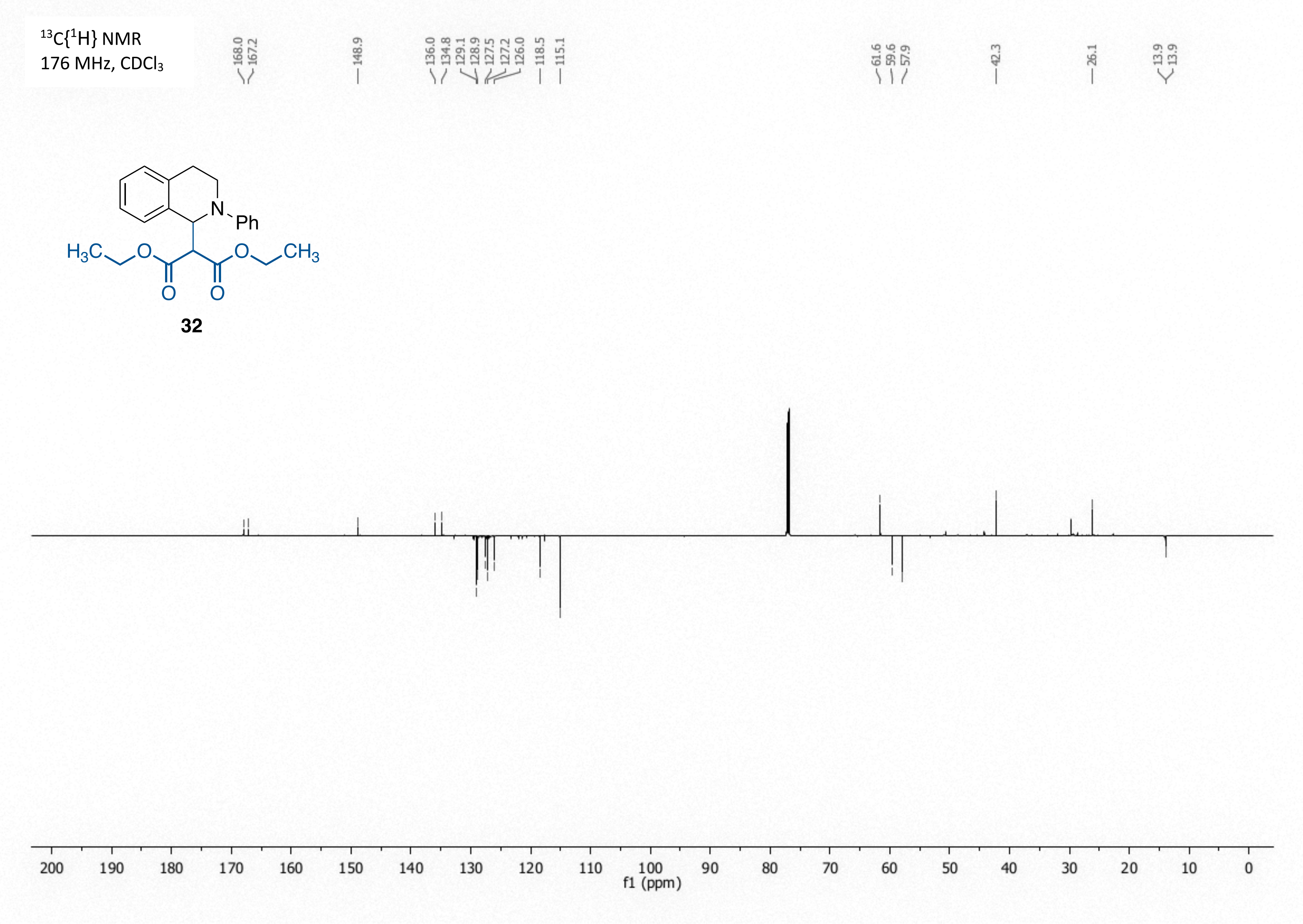
$^{13}C\{^{1}H\}$ NMR
176 MHz, $CDCl_3$
168.0
167.2
148.9
136.0
134.8
129.1
128.9
127.5
127.2
126.0
118.5
115.1
61.6
59.6
57.9
42.3
26.1
13.9
13.9
N
Ph
H_3C
O
O
CH_3
O
O
32
200 190 180 170 160 150 140 130 120 110 100 90 80 70 60 50 40 30 20 10 0
f1 (ppm)

^{1}H NMR
(700 MHz, $CDCl_3$)

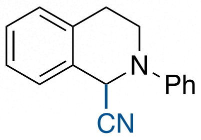

33

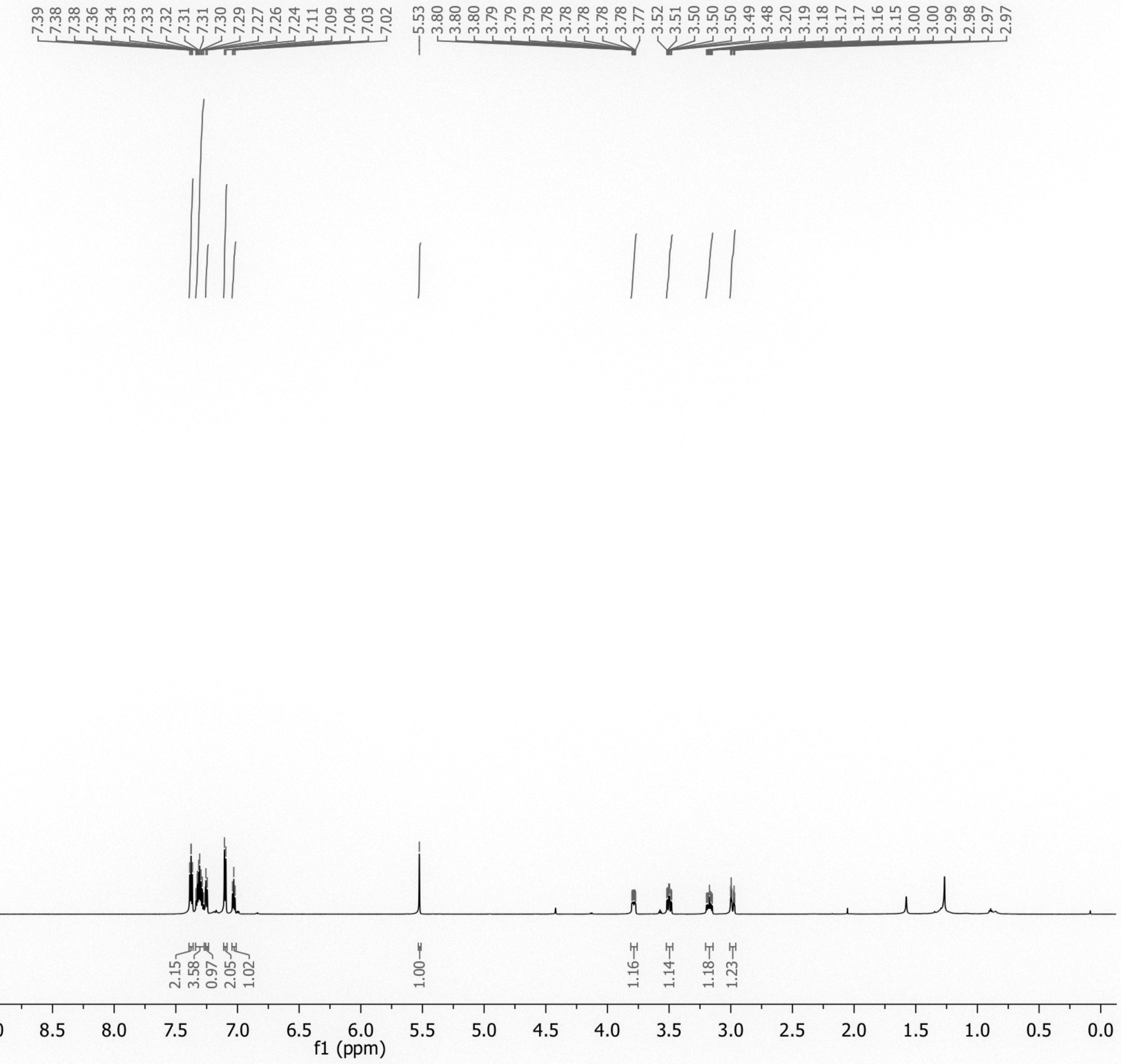

$^{13}C\{^{1}H\}$ NMR
176 MHz, $CDCl_3$

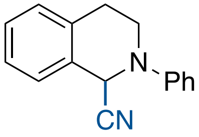

33

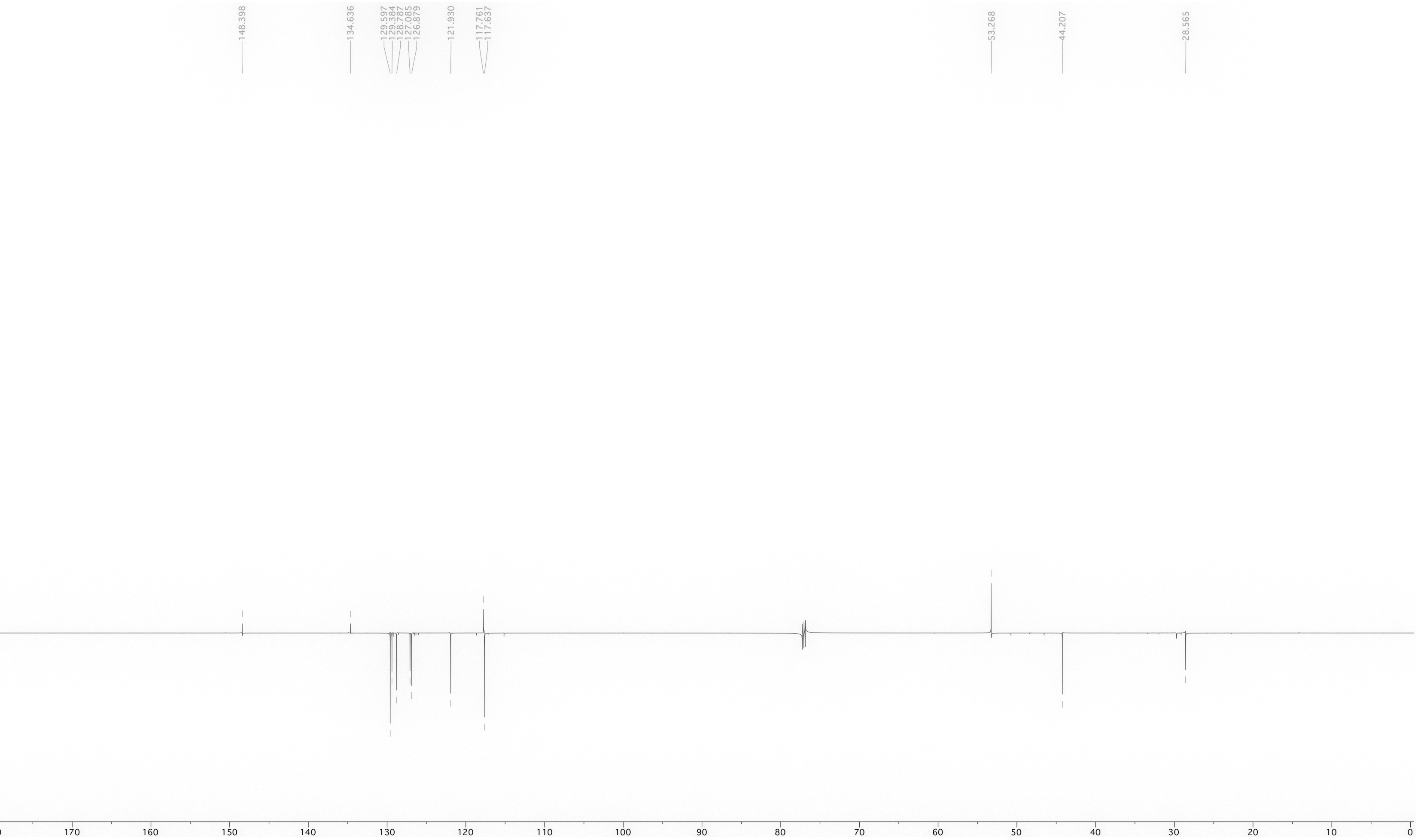

^1H NMR
700 MHz, $CDCl_3$

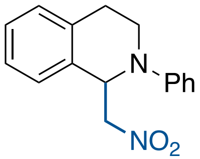

34

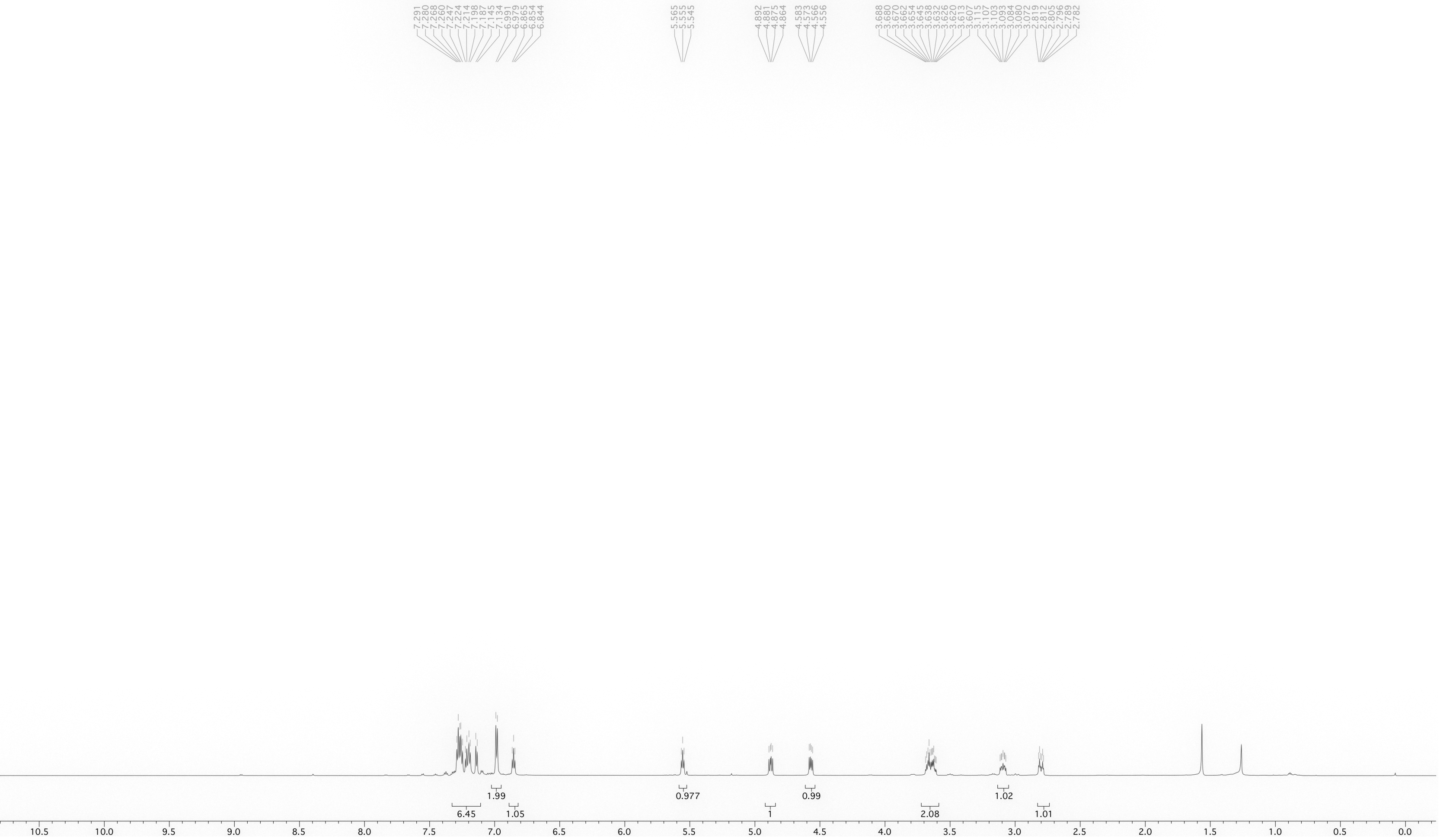

$^{13}C\{^1H\}$ NMR
176 MHz, $CDCl_3$

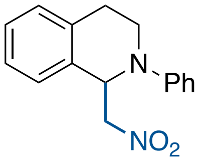

34

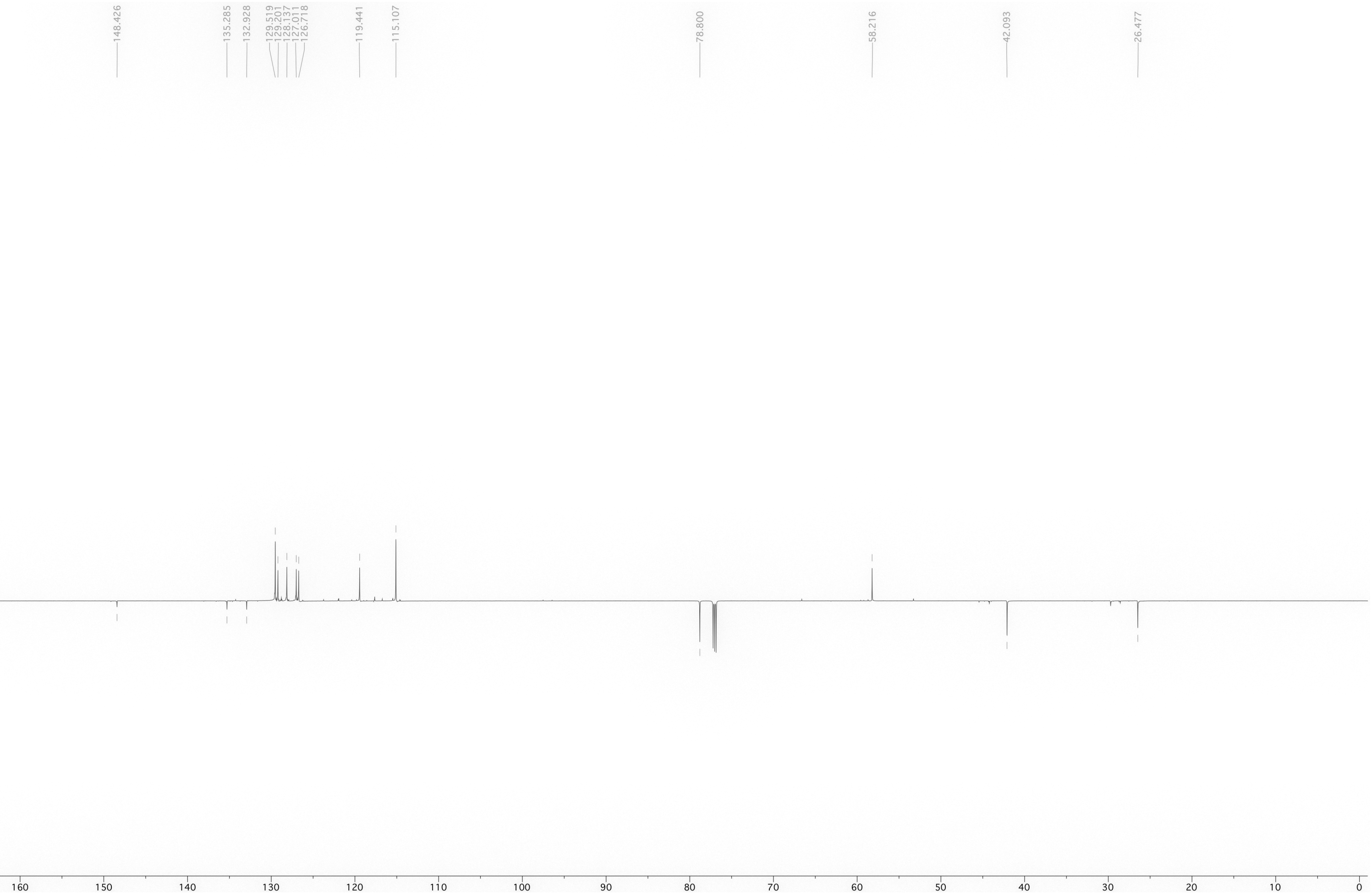

^1H NMR
700 MHz, $CDCl_3$

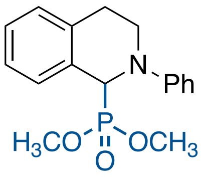

36

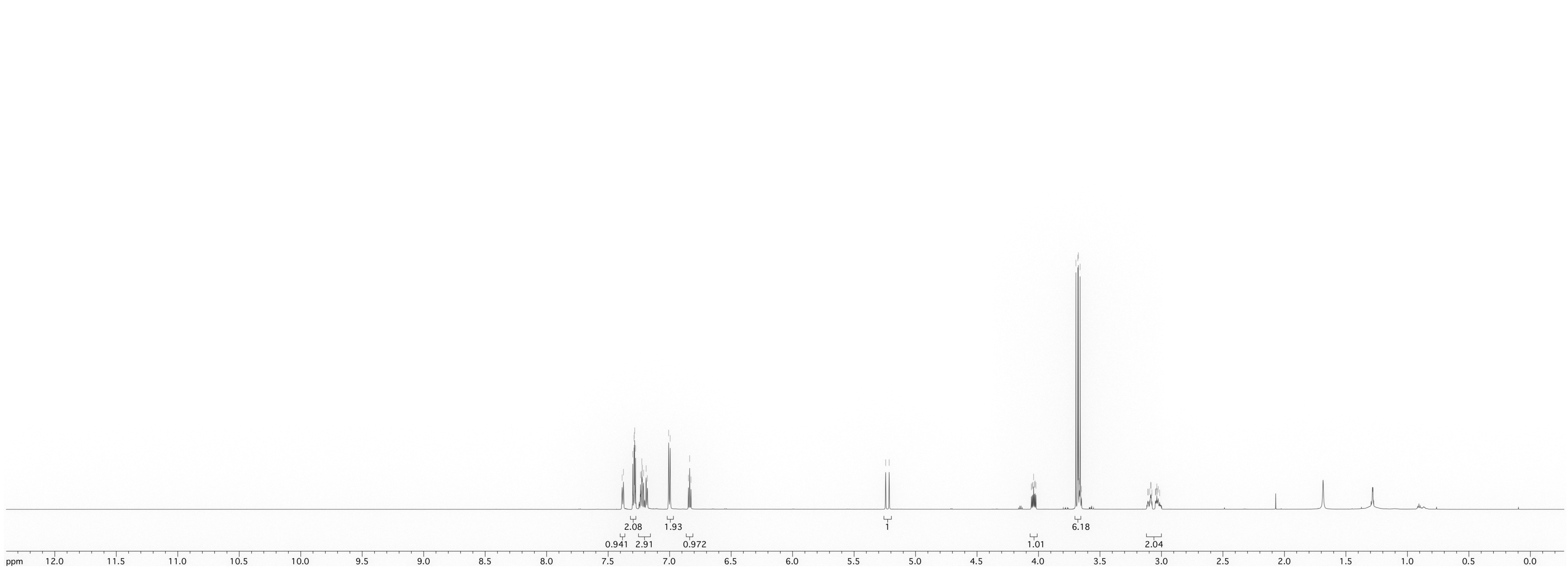

$^{13}C\{^{1}H\}$ NMR
176 MHz, $CDCl_3$

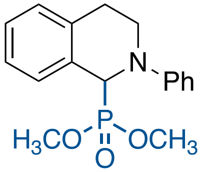

36

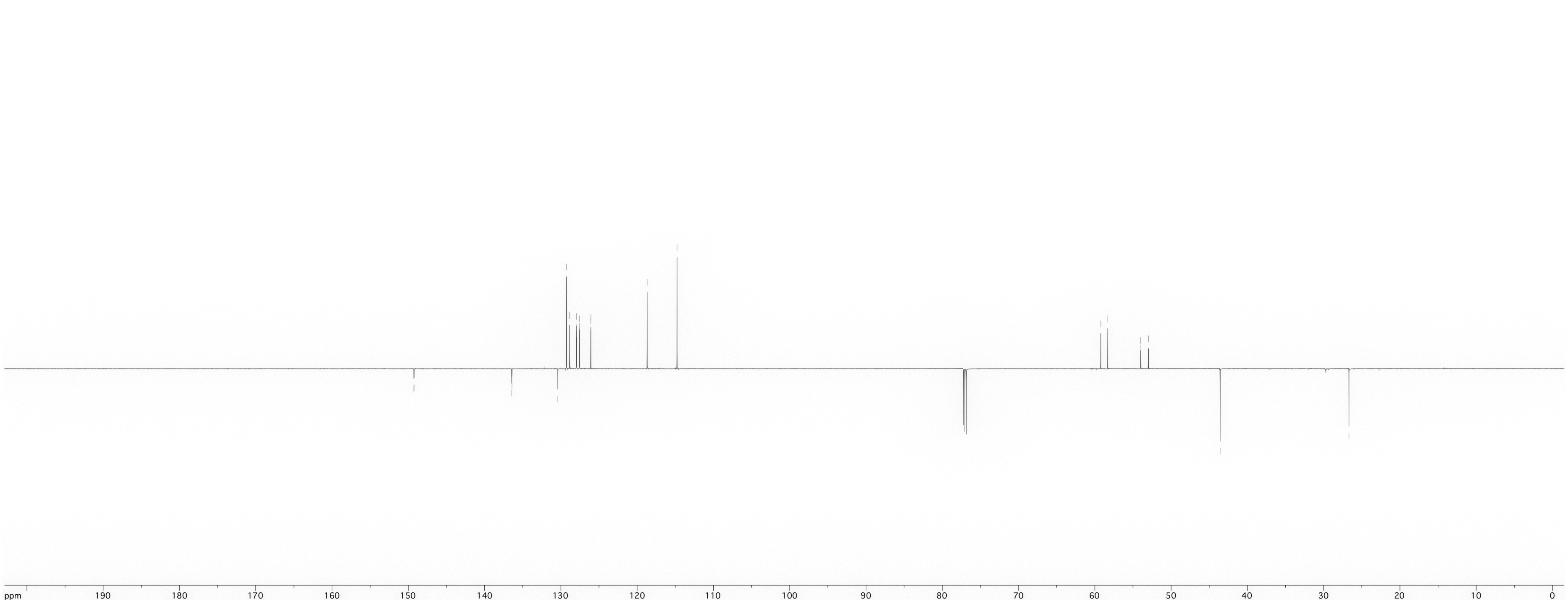

Supplement: Supplementary file 1 — jo1c02378_si_001.pdf [file jo1c02378_si_001.pdf]
